# Propofol Exposure Disturbs the Differentiation of Rodent Neural Stem Cells via an miR-124-3p/Sp1/Cdkn1b Axis

**DOI:** 10.3389/fcell.2020.00838

**Published:** 2020-08-27

**Authors:** Jun Cao, Yan Li, Fanning Zeng, Xiaolei Liu, Tao Tao, Zaisheng Qin

**Affiliations:** ^1^Department of Anesthesiology, Nanfang Hospital, Southern Medical University, Guangzhou, China; ^2^Department of Anesthesiology, Affiliated Shenzhen Maternity and Child Healthcare Hospital, Southern Medical University, Shenzhen, China; ^3^Department of Anesthesiology, Cangzhou Central Hospital, Cangzhou, China; ^4^Department of Anesthesiology, Affiliated Hospital of Guangdong Medical University, Zhanjiang, China; ^5^Department of Anesthesiology, The Central People’s Hospital of Zhanjiang, Zhanjiang, China

**Keywords:** propofol, neural stem cells, cell cycle, differentiation, miR-124-3p, Sp1, cdkn1b

## Abstract

Accumulating studies have indicated that propofol may lead to neurotoxicity and its effect on neural stem cells (NSCs) may play pivotal role in propofol-related neurotoxicity. Previously, we found that propofol could promote NSCs proliferation and could regulate several microRNA expressions. However, the underlying mechanism between microRNAs and NSCs development after propofol exposure is still unclear. Our data first observed that rat primary neural stem cells exposed to propofol exhibited a cell cycle arrest status and an inclination to differentiate into GFAP^+^ or S100β^+^ cells. This phenomenon was accompanying with a lower miR-124-3p expression and could be reversed via overexpression miR-124-3p in NSCs. Using bioinformatic predictions and luciferase assay we confirmed that Sp1 (Specificity Protein 1) is the target gene of miR-124-3p, indicating that miR-124-3p may regulate NSCs development through Sp1. Further, knockdown of Sp1 rescue the effect of propofol on NSCs differentiation. Finally, we demonstrated that Sp1 could bind cdkn1b promoter region through chromatin immunoprecipitation assay, indicating that Sp1 affect NSC’s cell cycle through cdkn1b directly. Overall, our study highlights the miR-124-3p/Sp1/cdkn1b axis to be important in propofol interfering the differentiation of NSCs.

## Introduction

Propofol is utilized worldwide as an intravenous anesthetic due to its rapid onset and minimal negative postoperative effects (Glen, 2018). However, propofol is still an off-label choice in most clinical pediatric practices (Chidambaran et al., 2015). The current dilemma is that there is a lack of evidence to support the safe use of propofol and there are a growing number of pre-clinical studies attributing neurotoxicity and neurogenic impairment to propofol (Krzisch et al., 2013; Bosnjak et al., 2016; McCann and Soriano, 2019).

It has been suggested that propofol can disrupt neurogenesis by modulating apoptosis, proliferation, or the differentiation of neural stem cells (NSCs) (Zou et al., 2013). The potential mechanisms underlying these effects include regulation of the caspase-3 cascade (Karen et al., 2013), calmodulin-dependent protein kinase II, or microRNAs (miRNAs) (Hebert and De Strooper, 2009; Liang et al., 2019). However, the roles of miRNAs in the dysfunction of NSCs following propofol exposure are not fully understood.

miRNAs are enriched in the nervous system and are key post-transcriptional regulators within neurodevelopment (Hebert and De Strooper, 2009; Liu and Xu, 2011). miR-124 is abundantly expressed in the brain where it participates in a complex relationship within central nervous system functions and disorders (Sun et al., 2015). During embryonic neurodevelopment, miR-124 is essential for cell survival in the cortex and loss of miR-124 results in neuronal apoptosis (Sanuki et al., 2011). Moreover, loss of miR-124 in the neural crest cells results in apoptosis of sympathetic ganglia and midbrain dopaminergic neurons (Huang et al., 2010). At the early postnatal stage, miR-124 triggers the outgrowth of mossy fibers in the dentate gyrus (Sanuki et al., 2011). While in the adult brain, miR-124 functions as an important regulator of the transition from transit amplifying cells to neuroblasts during neurogenesis in the subventricular zone (Cheng et al., 2009). These investigations suggest that the temporal and spatial equilibrium of miR-124 is crucial to the development of NSCs.

Previously, ourselves and others have demonstrated the ability of propofol to perturb the development of NSCs (Krzisch et al., 2013; Tao et al., 2013; Qiliang et al., 2016). Once lineage progression is initiated, NSCs acquire properties of differentiated cells, such as fate specification and specific morphologies. This switch requires potent regulators such as miRNAs, transcription factors, and RNA-binding proteins, in order to modulate the expression of multiple gene networks. Through bioinformatic analyses, Marcia et al., reported that miR-124 can regulate neurogenesis by targeting specificity protein 1 (Sp1) (Santos et al., 2016). Sp1 is a zinc finger structural transcription factor involved in cell cycle progression (Billon et al., 1999; Opitz and Rustgi, 2000; Cen et al., 2008), development, and differentiation (Palazuelos et al., 2014; Lee et al., 2020). Studies have identified that up-regulating Sp1 in mesenchymal stem cells could decrease neuronal differentiation (Mondanizadeh et al., 2015), whilst down-regulation could reduce the proliferation and neuronal production of NSCs during neurogenesis (Santos et al., 2016). However, there is still no direct evidence that miR-124 can target Sp1 in NSCs.

In the current study, we provide direct evidence that miR-124 can directly interact with Sp1 to regulate the differentiation of NSCs. Moreover, our study demonstrates that propofol exposure alter the differentiation of NSCs via a miR-124/Sp1/cdkn1b axis.

## Materials and Methods

### Culture of NSCs and Propofol Exposure

All experimental procedures were approved by the Southern Medical University Administrative Panel on Laboratory Animal Care, and experiments were conducted in accordance with the guidelines of Animal Use and Care of Southern Medical University. NSCs were harvested from both the cortices and hippocampi of Sprague–Dawley rat embryos on embryonic day 16–18 (E16-E18). Briefly, the brain tissue was collected and dissociated mechanically into single cells. To form neurospheres, cells were cultured in NSC basal medium (Millipore, United States) containing basic fibroblast growth factor 20 ng/mL (R&D, United States), then incubated at 37°C and 5% CO_2_. After 3–5 days in culture, neurospheres of 150–200 μm in diameter were digested into single cells using Accutase (Millipore, United States) and suspended to a density of 5 × 10^5^ cells/ml. Cells were then plated on poly-L-ornithine and laminin-coated plates (Sigma-Aldrich, United States) in NSC basal medium for 2–3 days. The culture medium was then replaced with fresh and Dulbecco’s Modified Eagle’s medium (DMEM)/F12 containing 2,6-diisopropylphenol (propofol; Sigma-Aldrich) at a final concentration of 50 μM in dimethyl sulfoxide (DMSO) (Sigma-Aldrich, United States) (Li et al., 2018). The same volume of DMSO was added to the control group. Cells were treated for 6 h prior to differentiation.

### NSCs’ Differentiation

To induce differentiation of NSCs, cells were grown for 3 days in DMEM/F12 and 10% FBS. NSCs were stained for neuronal and glial cell markers using mouse anti-β-tubulin III (1:300 dilution; Proteintech; China; Cat# 66375-1-Ig;RRID:AB_2814998) and rabbit anti-GFAP (1:300 dilution; Abclonal; China; Cat# A14673, AB_2761548), respectively.

### Immunocytochemistry

Fluorescent staining of nestin using rabbit anti-nestin (1:200 dilution; ABclonal; China; Cat# A0484; AB_2757216) was to identify NSCs. And fluorescent staining of anti-β-tubulin III and anti-GFAP (mentioned above) was performed to confirm NSC differentiation. Briefly, cells were washed once with phosphate-buffered saline (PBS), fixed for 30 min in 4% paraformaldehyde (Solarbio, China) at 37°C, and permeabilized with 0.5% Triton X-100 (Sigma-Aldrich) for 10 min. After three 5 min washes with PBS, the cells were blocked with 1% bovine serum albumin (BSA; Solarbio, China) for 1 h at room temperature. Cells were incubated overnight at 4°C with primary antibodies (diluted in 1% BSA). The cells were washed three times with PBS-Tween-20 (0.1% v/v) and were incubated for 1 h at room temperature with fluorescently labeled secondary antibodies including FITC-conjugated goat anti-rabbit IgG [(1:100 dilution; Bioss; China; Cat# bs-0295G-FITC; AB_10894349], Cy3 conjugated goat anti-mouse IgG ((1:100 dilution; Bioss; China; Cat# bs-0296G-Cy3; B_10892835), Cy3-conjugated goat anti-rabbit IgG (1:100 dilution; Bioss; China; Cat# bs-0295G-Cy3; AB_10892956) and DyLight 405 goat anti-mouse IgG antibody (1:100 dilution; Abbkine, United States; Cat# A23110; AB_2721248). After washing, cells were counterstained with DAPI and analyzed using laser-scanning confocal microscopy (Olympus, Japan). Cell numbers in culture were counted in 5 fields per well (center and at 3, 6, 9, and 12 o’clock positions) and summed for the entire well. The percentage values of each positive cells were calculated based on the sum of two positive cells. Four duplicated wells in each group from five independent experiments were analyzed. All results were confirmed by 2 researchers using double-blind method.

### MicroRNA Target Prediction and Screening

MiRWalk2.0^1^, a collection of predictions and experimental verifications of miRNA-targets (Dweep and Gretz, 2015) was used in the current study to predict the target of miR-124-3p. Target mRNA with predicted binding sites for miR-124-3p were identified using the following databases: miRWalk, miRanda, miRDB and TargetScan. The bioinformatics data was analyzed using the DAVID Bioinformatics Resources 6.8^2^ (Huang da et al., 2009) for Gene ontology enrichment. Venn diagrams were generated using online tools^3^.

### Luciferase Reporter Assay

HEK293T cells were seeded at 50% confluence 24 h prior to transfection. Wild-type (WT) or mutant (MUT) Sp1 3′-UTR reporter constructs were co-transfected along with an miR-124-mimic or negative control (NC) using Lipofectamine 2000 (Invitrogen). At 48 h post-transfection, luciferase assays were performed using a Dual-Luciferase Reporter assay system (Promega United States) according to manufacturer‘s instructions and analyzed on a multi-plate reader (BioTek, United States). Relative light units were calculated by the ratio of Renilla to firefly luciferase activity. The control psiCHECK-2 plasmid that carried the 3′-UTR region of Sp1 gene was used to normalize to and correct non-specific effects. Three technical replicates were performed for each condition.

### miR-124-3p Overexpression

To determine the effects of miR-124 on the cells, they were transfected with 50 nM of an miR-124-3p mimic (Genepharma) or/NC with Lipofectamine 2000 reagent (Invitrogen, United States) according to the manufacturer’s instructions.

### RNA Extraction and Quantitative Real-Time PCR (qRT-PCR)

Total RNA was isolated from primary NSCs using TRIzol reagent (Thermo Fisher Scientific, United States) according to the manufacturer’s instructions. Total RNA (1 μg) was used to synthesize cDNA using a PrimeScript RT reagent Kit with gDNA Eraser (TaKaRa, China). miRNAs were isolated using RNAiso (TaKaRa, China) according to manufacturer’s instructions. miRNA (5 μg) was polyadenylated and used to synthesize cDNA using a MirX miRNA First Strand Synthesis kit (Clontech, Japan). Expression of mRNA and miRNA was determined by quantitative real-time PCR (qRT-PCR) using the TB Green Premix Ex Taq II (TaKaRa, China) and MirX miRNA qRT-PCR SYBR Kit (Clontech, Japan), respectively. qRT-PCR was performed on the ABI 7500 system (Applied Biosystems, United States). β-actin and U6 expression was quantified as internal controls for mRNA and miRNA analysis, respectively. The primers sequences used in these analyses can be found in the (Supplementary Table 1). The results of the analyses were calculated and expressed according to an equation (2^–ΔΔCt^) which provides the amount of the target, normalized to an internal reference. Ct is a threshold cycle for target amplification. Each biological sample was tested in triplicate.

### Lentiviral Vector Transduction

NSCs were transduced with Sp1 short-hairpin RNA (shRNA) or NC lentivirus (Obio Technology). Virus-containing medium was replaced with the differentiation medium mentioned above. For lentiviral transduction, NSCs (4 × 105) were seeded in 6-well plates and the lentivirus was added at a multiplicity of infection (MOI) of 1:20. After 72 h, the transduction efficiency was evaluated via fluorescence microscopy. Three shRNAs which targeted different gene regions were explored to obtain the most effective silencing. Sense strands used in this study can be found in the (Supplementary Table 1).

### Western Blot

NSCs were harvested and digested in RIPA extraction buffer (Beyotime, China). Protein samples were separated by 10% SDS-PAGE and transferred onto PVDF (polyvinylidene difluoride) membranes (Millipore, United States) in tank transfer system (Bio-Rad, United States). Membranes were blocked with 5% non-fat milk in Tris-buffered saline containing 0.1% Tween-20 (TBST) for 1h, washed three times in TBST, and incubated overnight at 4°C with primary antibodies including rabbit anti-Sp1 (1:1000 dilution; Abcam; United States; Cat# ab13370, AB_300283), rabbit anti-cdkn1b (1:1000 dilution; Abcam; United States; Cat# ab32034, AB_2244732), rabbit anti-GAPDH (1:2500 dilution; Abcam; United States; Cat# ab9485; AB_307275), or rabbit anti-β-tubulin(1:1000 dilution; Abcam; United States; Cat# ab6046; AB_2210370). After incubation with the HRP conjugated goat anti-rabbit IgG secondary antibody (1:5000 dilution; Bioss; China; Cat# bs-0295G-HRP, AB_10923693), immunoreactive bands were detected by enhanced chemiluminescence (Millipore, United States). The protein bands were quantitatively analyzed using ImageJ software 1.52a.

### Chromatin Immunoprecipitation

Chromatin immunoprecipitation (ChIP) for the primary NSCs was performed using a Pierce Magnetic ChIP Kit (Thermo Fisher Scientific, United States) according to the manufacturer’s instructions. An anti-Sp1 antibody suitable for ChIP (1:100 dilution; Cell Signaling Technology; United States; Cat# 9389; AB_11220235) or rabbit IgG (1:250 dilution; Thermo Fisher Scientific; United States; Cat# 31887; AB_2532177) was use. qRT-PCR was performed to obtain quantitative data using 2 × Taq Plus Master Mix (Vazyme, China), and TB Green Premix Ex Taq II (TaKaRa, China). The enrichment at the cdkn1b promoter region was normalized to the amount of the total input. The Primers for the cdkn1b promoters can be found in the (Supplementary Table 1).

### Statistical Analysis

For data obtained via qRT-PCR or Western blot, two-way ANOVA with repeated measures was used to analyze the differences between propofol-treated and control groups at various time points. All other data were analyzed via one-way ANOVA. *p* < 0.05 was considered statistically significant.

## Results

### Propofol Exposure Promotes the Differentiation of NSCs to GFAP^+^ Cells

**FIGURE 1:**
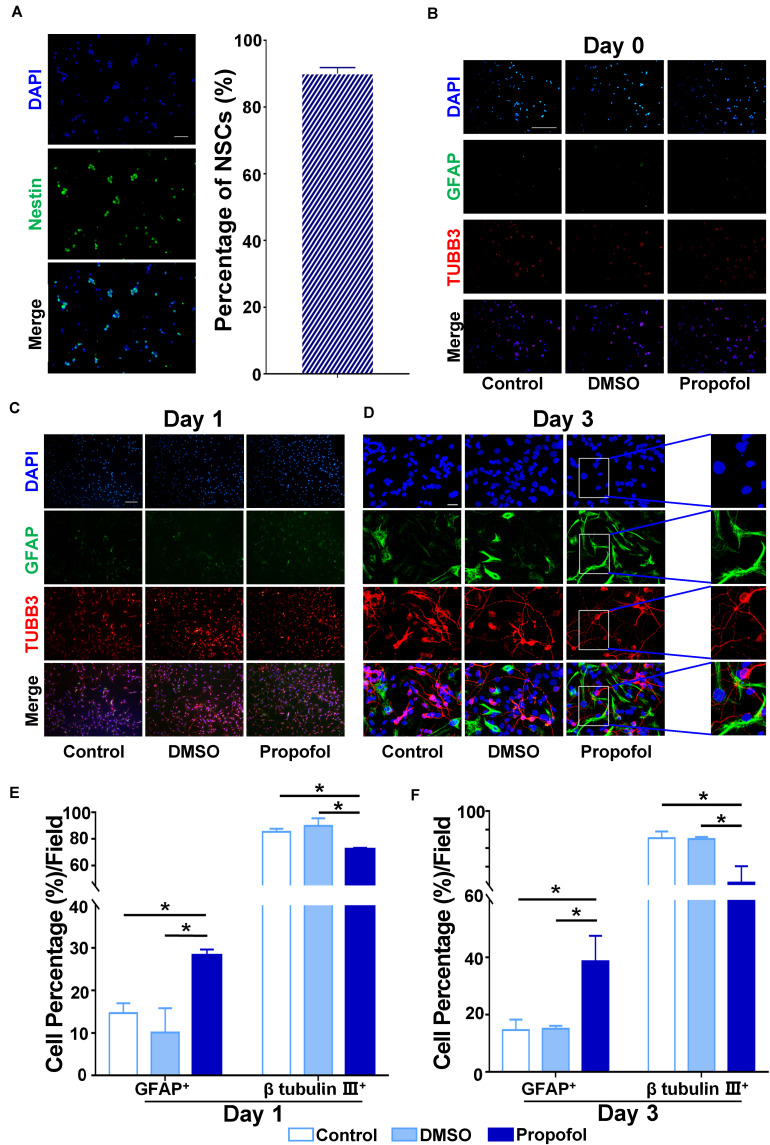
Propofol disturbs the differentiation of rat NSCs. **(A)** Identification of primary cultured NSCs immunostained with anti-nestin antibody (green) and counterstained with DAPI (blue). Purity of primary cultured NSCs was calculated by cell counting. **(B)** Immunofluorescent images displaying the influence of propofol exposure on the expression of β-tubulin III and GFAP on day 0 **(C)** day 1 **(D)** or day 3 after inducing differentiation of NSCs. **(E)** The percentage of β-tubulin III^+^ or GFAP^+^ cells were quantified on day 1 **(F)** and day 3 after NSC differentiation. Statistical significance of mean differences was determined with the Tukey’s multiple comparisons test. Error bars represent SD (*n* = 5). *P < 0.05. Scale bar in **(A)** represents 10 μm, 100 μm in **(B,D)** and 20 μm in **(D)**. TUBB3 = β tubulin III.

Immunocytochemistry identified that >90% of the cells isolated from the rat embryonic cortex and hippocampus were nestin positive (Figure 1A). In order to evaluate whether propofol exposure had an influence on the differentiation of NCSs, the cells were treated for 6h with 50 μM propofol before the induction of differentiation. Antibodies against the immature neuron marker β-tubulin III and glial marker GFAP were used for immunocytochemical staining on day 0, day 1, and day 3 after inducing differentiation. We found that NSCs had differentiated into both β-tubulin III+ and GFAP+ cells on day 1 and 3 (Figures 1B–D). However, following treatment with propofol, the proportion of GFAP+ cells compared to DMSO-treated or control cells significantly increased (Figures 1E,F). Correspondingly, the proportion of β-tubulin III+ cells decreased (Figures 1E,F). Further, the fluorescence intensity indicated that the expression of S100β, another astrocyte’s marker, was upregulated in propofol and reversed in Sp1 knockdown group in day3 (Supplementary Figure 2).

### Propofol Downregulates miR-124-3p in Rat NSCs

**FIGURE 2:**
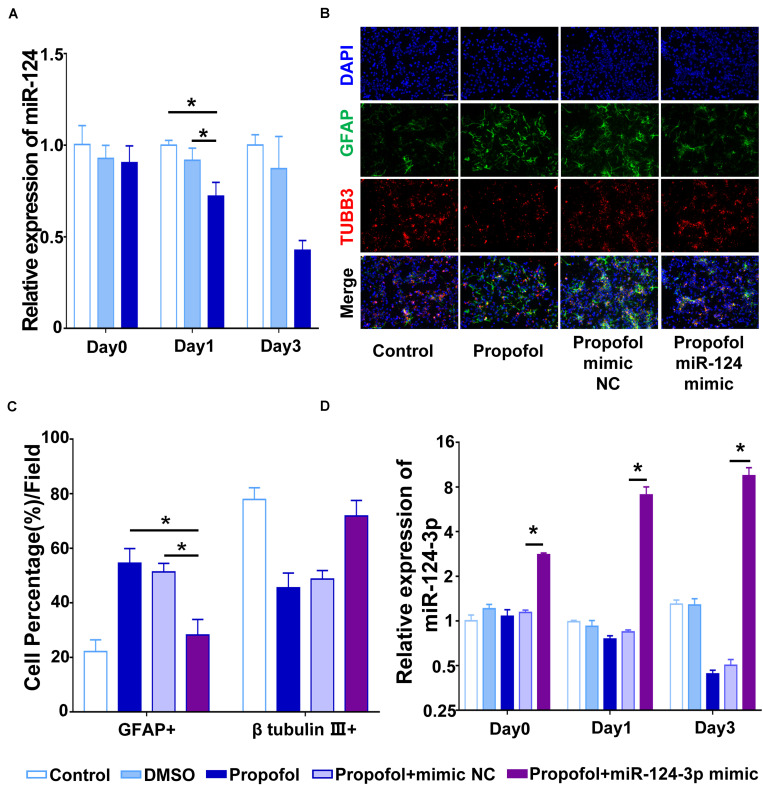
Propofol downregulates miR-124-3p in rat NSCs. **(A)** qRT-PCR showed that propofol exposure significantly downregulated miR-124-3p. **(B)** Immunofluorescent images indicated that miR-124-3p overexpression limited the number of GFAP^+^ cells. **(C)** The percentage of GFAP^+^ cells were quantified. **(D)** miR-124-3p expression was significantly upregulated following transfection of NSCs with an miR-124-3p mimic. Statistical significance of mean differences was determined with the Tukey’s multiple comparisons test. Error bars represent SD (*n* = 3). *P < 0.05. Scale bar represents 10 μm. TUBB3 = β tubulin III.

We then proceeded to investigate the mechanism by which propofol induced the differentiation of NSCs into GFAP+ cells. Based on our previous research, we selected several miRNAs involved in cell differentiation and assessed whether propofol could modulate their expression. Among the miRNAs investigated, only miR-124-3p was shown to be down-regulated on day 1 and 3 of differentiation following propofol exposure (Figure 2A). We then constructed an miR-124 mimic exogenously and transfected this into NSCs so that they overexpressed this miRNA. Our results showed that the miR-124 mimic could moderately reverse the effects of propofol and reduce the proportion of NSCs differentiating into GFAP+ cells (Figures 2B,C). The effect of miR-124-3p overexpression were confirmed at day 0, 1, and 3 (Figure 2D).

### miR-124 Binds to the 3′-UTR Regions of Sp1 mRNA

**FIGURE 3:**
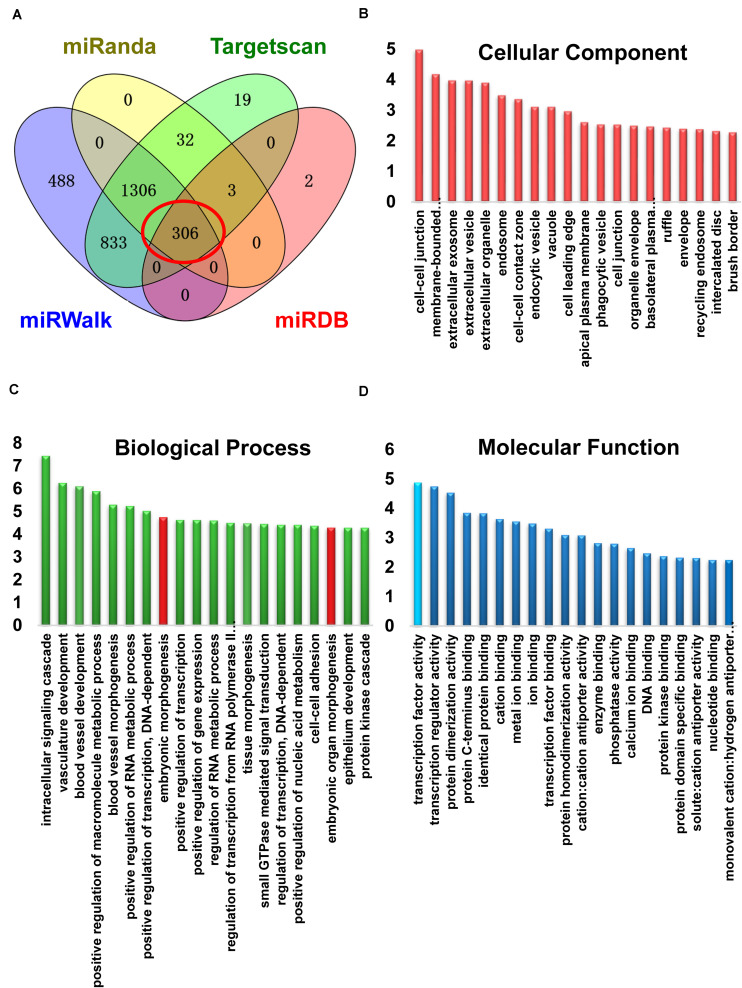
miR-124 targets Sp1 by binding to the 3′-UTR region of Sp1 mRNA. **(A)** Venn diagram showing target mRNAs for miR-124-3p from four databases. **(B)** Gene ontology enrichment analysis of target genes for miR-124-3p involved in cellular components **(C)** biological processes, or **(D)** in molecular functions. Development-related subsets containing Sp1 are marked in red. Transcription factors (sky blue) had the highest enrichment-score in molecular function.

miRNAs target the 3′-UTR regions of mRNA to induce post-transcriptional gene regulation. To predict the target mRNA of miR-124-3p, we utilized four online miRNA databases, miRanda, miRDB, miRWalk, and TargetScan (Supplementary Table 2). Here, we took the candidates that were predicted by all four databases and further analyzed these bioinformatically (Figure 3A). Gene ontology enrichment analysis showed that among targets within the molecular function, transcription factors had the highest enrichment-score (Figure 3D). Among the targets of top 20 enrichment-score involved in biological processes, we found two terms contained Sp1 and were related to development simultaneously (Figure 3C; Sp1 containing subsets shown in red). Finally, among the miR-124-3p targets involved in cellular component, targets involved in processes at the cell-cell junction were enriched (Figure 3B).

**FIGURE 4:**
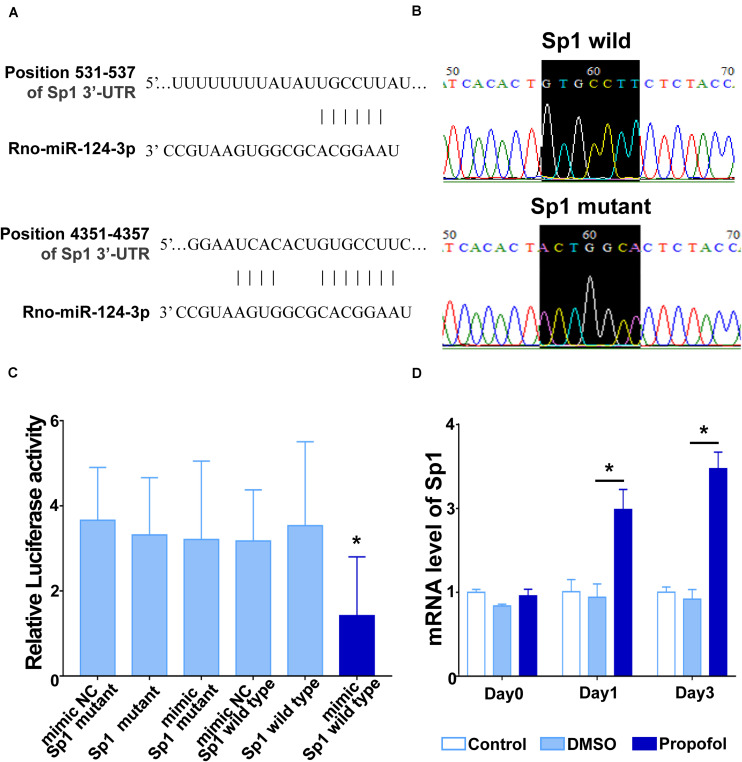
miR-124 targets Sp1 by binding to the 3′-UTR region of Sp1 mRNA. **(A)** A schematic diagram showing two complementary sites for miR-124-3p in the 3′-UTR regions of Sp1 mRNA. **(B)** Point mutation at the binding site for miR-124-3p in Sp1 mRNA. **(C)** Luciferase reporter assays in HEK293T cells after co-transfection of cells with the wild-type or mutant 3′-UTR of Sp1 and the miR-124-3p or NC mimics. **(D)** mRNA levels of Sp1 on day 1 and 3 of differentiation following exposure to propofol. Statistical significance of mean differences was determined with the Tukey’s multiple comparisons test (*n* = 3). Error bars represent SD. *P < 0.05.

We next used TargetScan and identified that the 3′-UTR regions of Sp1 mRNA contains two predicted miR-124-3p binding sites (Figure 4A). To verify these predictions, a dual luciferase reporter assay was applied. First, we generated a point mutation in the miR-124-3p binding site on Sp1 mRNA (Figure 4B). We then cloned the miR-124-3p binding regions from both the wild-type and mutated Sp1 into the Renilla luciferase coding sequence of the psiCHECK-2 vector. The miR-124-3p mimics or mimic NC were co-transfected with psiCHECK-2-Sp1-3′-UTR-WT or psiCHECK-2- Sp1-3′-UTR-MUT into HEK-293T cells. Compared with other groups, the luciferase activity in cells co-transfected with the miR-124-3p mimic and wild-type Sp1 was significantly reduced (Figure 4C). We also performed qRT-PCR and confirmed that propofol exposure significantly enhanced the expression of Sp1 mRNA on day 1 and 3 after inducing differentiation (Figure 4D).

### miR-124 Targets Sp1 to Differentiate NSCs Into GFAP^+^ Cells Following Propofol Exposure

**FIGURE 5:**
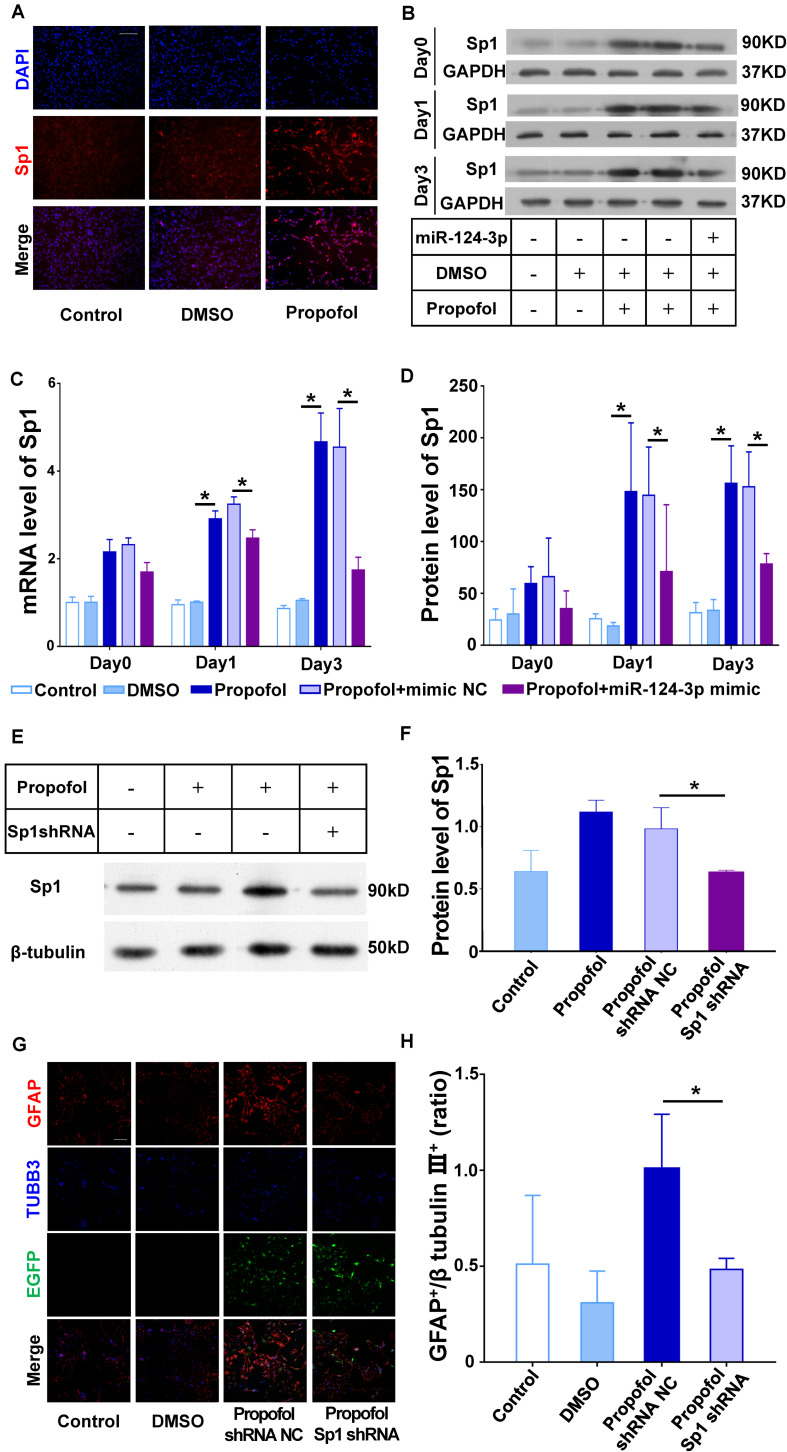
miR-124 targets Sp1 to induce differentiation of NSCs into GFAP^+^ cells following propofol exposure. **(A,B)** Protein and **(C)** mRNA expression of Sp1 could be inhibited in NSCs transfected with the miR-124-3p mimic. **(D)** Immunofluorescence images depicting Sp1 upregulation following propofol exposure. **(E,F)** Sp1 protein expression was significantly downregulated following transfection of NSCs with Sp1 shRNA lentivirus. **(G)** Sp1 knock-down inhibited the ability of propofol to induce differentiation of NSCs to GFAP^+^ cells. NSCs transfected successfully with lentivirus were marked with EGFP. **(H)** The ratio of GFAP^+^ to β tubulin III^+^ cells were quantified. Data are expressed as the mean ± SD. NSCs transfected successfully with lentivirus were marked with EGFP. Statistical significance of mean differences was determined with the Tukey’s multiple comparisons test. Error bars represent SD (*n* = 3). *P < 0.05. Scale bars represent 100 μm. TUBB3 = β tubulin III.

To further validate the effect of miR-124-3p on Sp1, we transfected the miR-124 mimic into NSCs and quantified Sp1 mRNA and protein expression. qRT-PCR, immunocytochemistry, and Western blots results showed that Sp1 mRNA and protein was significantly increased following propofol exposure (Figures 5A–D). And the increased mRNA and protein expression could be reversed by the miR-124-3p mimic (Figures 5B–D).

For further confirm the effect of Sp1 in NSCs’ differentiation, we knock-down the Sp1 expression in NSCs using shRNA (Figures 5E,F). And the results confirmed that knock-down of Sp1 could limit the increase of GFAP+ cells following propofol exposure (Figures 5G,H).

### Sp1 Binding to the cdkn1b Promoter Region Leads to Cell Cycle Arrest of NSCs

**FIGURE 6:**
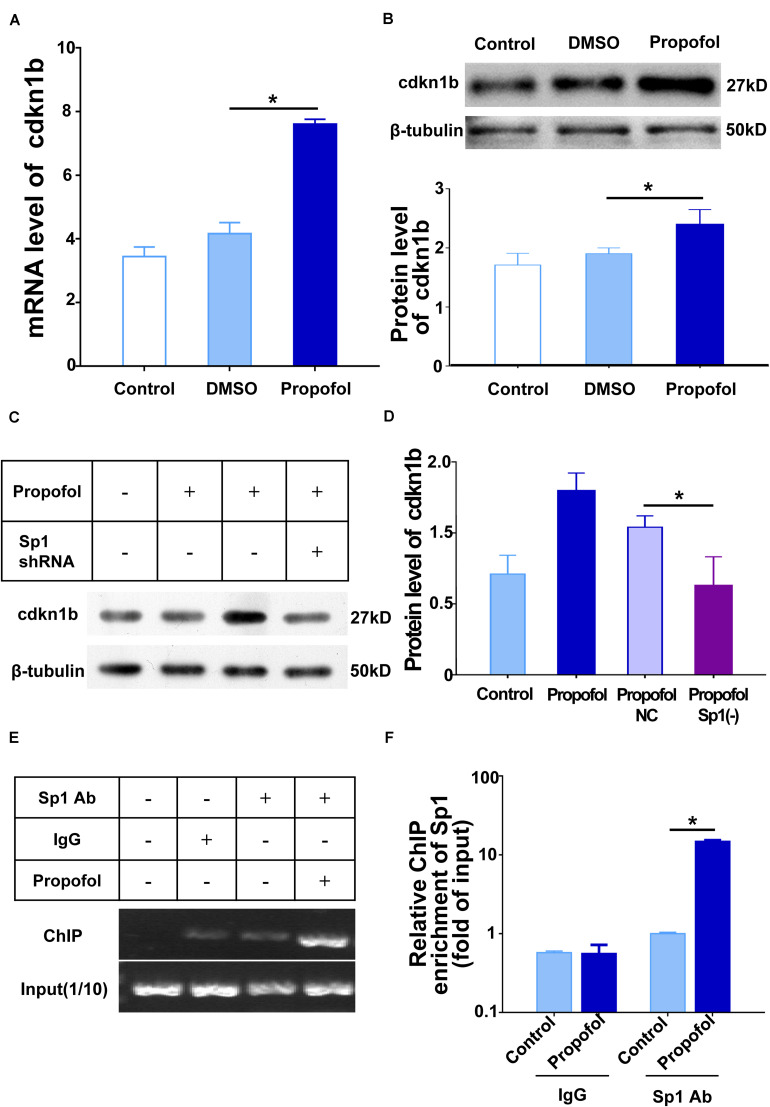
Sp1 binding to the cdkn1b promoter region induce cell cycle arrest of NSCs. **(A)** Propofol elevated the expression of cdkn1b mRNA and **(B)** protein during differentiation of NSCs. **(C)** Sp1 knock-down reduced cdkn1b mRNA and **(D)** protein expression. **(E,F)**. Sp1 binding to the cdkn1b promoter region in NSCs treated with propofol. IgG was used as a negative control. Statistical significance of mean differences was determined with the Tukey’s multiple comparisons test. Error bars represent the SD (*n* = 3). *P < 0.05. Sp1 Ab = Sp1 antibody.

Previous studies have shown that the cell fate of NSCs can be modulated by cyclin-dependent kinases (including CDK4 and CDK2) (Li et al., 2008), and cyclin-dependent kinase inhibitors (including cdkn1a and cdkn1b) (Andreu et al., 2015; Cheng et al., 2015). Accordingly, we performed qRT-PCR to explore the correlation between these cell-cycle proteins and propofol exposure (Supplementary Figure 1). Among those proteins, the mRNA expression of cdkn1b was markedly increased after propofol exposure (Figures 6A,B). Cell cycle detections showed the percentage of cells in G1 increased in propofol group compared to control group indicating a lengthening of the G1 phase in day1 and day3 (Supplementary Figure 3). Moreover, when exposed to propofol, knock-down Sp1 could significantly decreased cdkn1b protein expression level (Figures 6C,D). As a transcription factor, Sp1 may regulate transcriptional activity of several cell cycle regulatory proteins. Thus, we conducted chromatin immunoprecipitation (ChIP) to test whether Sp1 could directly regulate cdkn1b. Our results exhibited that cdkn1b promoter region was enhanced enrichment in NSCs after propofol treatment (Figures 6E,F).

## Discussion

In the current study, we investigated the effect of propofol on the fate of rat NSCs and the role of the miR-124/Sp1/cdkn1b axis in this process. Our data first highlighted that NSCs exposed to propofol exhibited a cell cycle arrest status and then an inclination to differentiate into GFAP^+^ or S100β^+^ cells. Moreover, propofol could decrease the expression of miR-124-3p. Using bioinformatic predictions and biological validation we demonstrated that miR-124-3p can interact with the 3′-UTR of Sp1. Further, interaction of Sp1/cdkn1b might induce cell cycle arrest which might be relative to tendency to differentiate into GFAP^+^ or S100β^+^ cells of NSCs. This inclination could be overturned by overexpression of miR-124 or knockdown of Sp1.

In previous studies, the data indicated that propofol would inhibit the proliferation of NSCs (Li et al., 2018; Liang et al., 2019). In our present study, propofol lengthened the G1 phase indicating a cell cycle arrest. Then we pay more attention to the effect of propofol on cell fate, such as changes in the direction of cell differentiation after cell cycle arrest. To better illustrate the two tendency of cell differentiation we calculated the percentage values of each positive cells based on the sum of two positive cells, GFAP^+^ and β-tubulin III^+^. Propofol treatment on NSCs, shows a tendency by differentiating into more GFAP^+^ cells, which could be a symbol of stemness or a symbol of astrocyte. In addition to GFAP, S100β, another specific marker for astrocytes differentiation, was up-regulated after propofol exposure in day 3 as well.

NSCs are critical within neurogenesis whereby they can self-renew and differentiate into neurons or glial cells (Kriegstein and Alvarez-Buylla, 2009; Wegleiter et al., 2019). Previous studies have shown that excessive gliogenesis during neural differentiation underlies the pathophysiology of several neural disease models (Bailey et al., 2013; Allen et al., 2017; Umezawa et al., 2018). In these diseases, excessive production of GFAP+ cells are correlated to synaptic dysfunction and brain perivascular abnormalities during neurodevelopment (Hussaini and Jang, 2018). Later in life, these pathophysiological changes will lead to learning and memory deficits and social behavioral disorders (Cai et al., 2019).

It has been well characterized that propofol affects neurogenesis via its actions on NSCs. Propofol induces autophagy in neural progenitor cells (NPCs) associated with endoplasmic reticulum Ca^2+^ release via InsP3Rs activation, though direct mechanics of how propofol modulate InsP3Rs is unknown. It is also known that propofol exposure regulates cell-fate by triggering differentiation of human NPCs into GFAP+ cells; which is similar to our observations here with rat NSCs (Qiao et al., 2017). However, evidence is still lacking for the link between propofol-induced differentiation of NSCs into GFAP+ cells and alterations in neural function.

Several studies have implicated the importance of miRNAs in the development of neural functions after propofol exposure. It is reported that repeated exposure to propofol results in down-regulation of miR-132 and significantly decreased numbers of dendritic spines in the hippocampus (Zhang et al., 2017). Furthermore, our previous study described the ability of propofol to modulate miRNAs in NSCs (Fan et al., 2016; Li et al., 2018). In this study we found that miR-124-3p was downregulated and was a crucial regulator of NSC differentiation following propofol exposure.

Given that miR-124-3p is the most abundant miRNAs in the developing and matured brain (Sun et al., 2015), the lack of miR-124-3p is related to the pathogenesis of several diseases. Similarly to our research, in Parkinson’s disease, deficiency of miR-124-3p delivery to the subventricular zone impairs neurogenesis and neural cell differentiation due to reduction of silencing the target cell-fate proteins Sox9 and Jagged1 (Saraiva et al., 2016). Besides silencing cell-fate relative mRNA, it is also reported that the lack of miR-124 will lead to the inability to precisely regulate the epigenetic regulatory factors in neuroblastoma cells to regulate the transition to neurons and astrocytes (Neo et al., 2014). During neurodevelopment, miR-124 temporally regulates the transition from transit amplifying cells to neuroblasts (Cheng et al., 2009). By repression of polypyrimidine tract-binding protein (PTBP), miR-124 can induce *trans-*differentiation of fibroblasts into functional neurons (Xue et al., 2013). Accordingly, it is rational to speculate that in our current study propofol led to cell arrest and alteration in differentiated tendency is closely relative to dysfunction of miR-124-3p and its target. Additionally, beside its effects on neuronal fate, miR-124 also contributes to promoting neurite outgrowth during neuronal differentiation (Gu et al., 2018). However, the duration of propofol on miR-124-3p in our study was within 3 days, which is not long enough for neurite development.

In order to better understand the mechanism by which propofol modulates differentiation of NSCs, we utilized bioinformatics and reporter assays to discover and validate Sp1 as the direct target of miR-124-3p. Moreover, Sp1 could be upregulated by propofol, while Sp1 knock-down reduced the number of GFAP+ cells following propofol exposure. Thus, highlighting Sp1 as an important factor in the differentiation of NSCs by miR-124-3p.

Sp1 is a DNA-binding protein, which activates and inhibits gene transcription in multiple physiological and pathological processes (Vizcaino et al., 2015; Wei et al., 2016). During gliogenesis, Sp1 was proven to binding to the promoter of GFAP, the expression of which was enhanced (Yeo et al., 2013; Johnson et al., 2016). The loss of Sp1 in astrocytes is linked to learning and memory impairment in mice by GFAP decrease (Hung et al., 2020). In our study, propofol increase the proportion of GFAP^+^ cells after induced differentiation. It is reasonable to believe that this alteration in differentiation tendency is modulate by Sp1. On the other hand, Sp1 mediates neurogenesis through the regulation of cell cycle-related proteins in multiple cell types (Billon et al., 1999; Opitz and Rustgi, 2000; Cen et al., 2008). Binding of Sp1 to Cyclin D orchestrates cell fate decisions in human stem cells, represses neuronal differentiation in mesenchymal stem cells (Mondanizadeh et al., 2015; Pauklin et al., 2016). Coincided with these researches, our presented study suggested that Sp1 modulated cell cycle through binding enhancement to cdkn1b promoter.

Previous studies suggest that cell fate can also be moderated by cyclin dependent kinases inhibitors (CDKI) (Cunningham et al., 2002; Andreu et al., 2015; Abbastabar et al., 2018), transcription of which can be regulated by Sp1 (Cen et al., 2008). CDKIs inhibit CDKs to delay or stop cell cycle progression (Besson et al., 2008). One function of CDKIs is to control cell differentiation and proliferation in tumorigenesis or neurogenesis (Besson et al., 2008). Our data identified that Sp1 binding to the promoter region of cdkn1b (a key CDKI) elevates the protein level of cdkn1b. In neurodevelopment, it has been well demonstrated that cdkn1b accumulates in quiescent adult hippocampal neural stem cells *in vitro* (Andreu et al., 2015). In our current study, NSCs prone to differentiation into more GFAP^+^ cells (also known as a neural precursor cell marker) could be a symbol of stemness due to quiescent stage accumulate of NSCs. Further, cdkn1b induces cell-cycle arrest and facilitates neuronal differentiation in the adult hippocampus (Andreu et al., 2015). Our results also suggested a cell-cycle arrest probably resulted of cdkn1b increase after propofol exposure which therefore was prone to differentiation into GFAP^+^ or S100β^+^ cells. These could also be a sign of astrocyte differentiation. In all, NSCs’ cell-cycle arrest mediated by cdkn1b could possibly lead to the alteration in differentiation inclination. But the in-depth mechanism needs further investigation in our further research.

**FIGURE 7:**
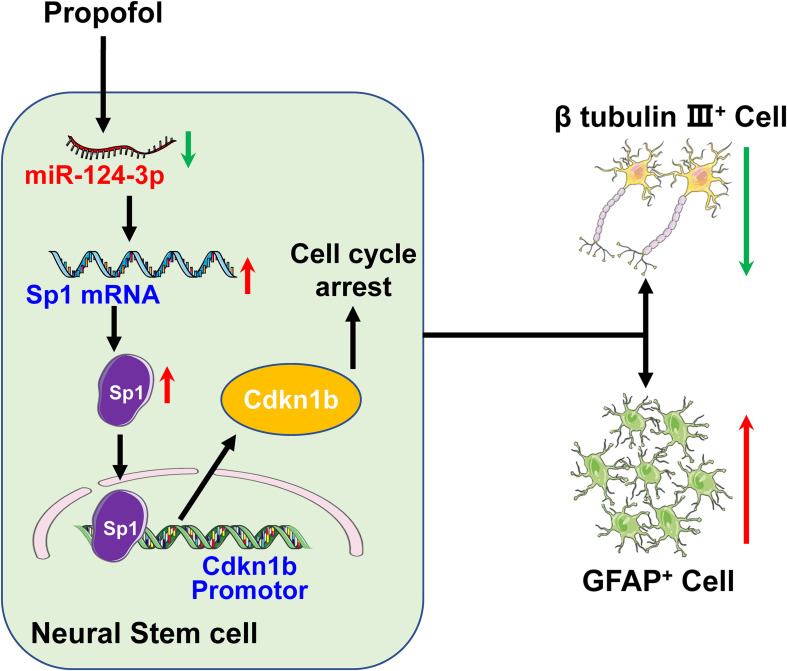
Propofol disturbs NSCs differentiation via a miR-124-3p/Sp1/cdkn1b axis.

In summary, the present study demonstrates that propofol exposure reduces miR-124-3p expression which results in upregulation of Sp1, increased cdkn1b transcription. As such, our research highlights the importance of the miR-124-3p/Sp1/cdkn1b axis in cell-fate modulation exhibiting a cell cycle arrest status and an inclination to differentiate into GFAP^+^ or S100β^+^ cells after propofol exposure (Figure 7).

Interpreting the data presented in our study, some limitations must be considered. Firstly, since multiple targets for propofol on cells existing, the pathway through which propofol enters the cells to perform its function is still a not clear. Therefore, it is difficult to involved the exact mechanism by which propofol may affect miRNAs in current study. Indeed, it will be more profound if we verified the results *in vivo*. In order to expound the most concern about whether propofol is toxic to developing brain, experiments of propofol exposure *in vivo* are required to operate in fetal or neonatal animals. However, the related animal models are still controversial, for the process of neurodevelopment *in vivo* is regulated by a complex network. Exposed to propofol, the phenotype *in vivo* is not necessarily clear. Therefore, the main purpose of this study is to clarify the effect and mechanism of propofol on neural stem cells to guide our further research *in vivo*.

## Data Availability Statement

The raw data supporting the conclusions of this article will be made available by the authors, without undue reservation, to any qualified researcher.

## Ethics Statement

The animal study was reviewed and approved by the Southern Medical University Administrative Panel on Laboratory Animal Care.

## Author Contributions

ZQ and TT conceived and coordinated the study. JC performed all experiments, data analysis, and wrote the manuscript. YL performed and analyzed the qPT-PCR experiments. XL performed the immunofluorescent image acquisition. YL and FZ performed the bioinformatics analysis. FZ performed the cell cycle detection in supplement. ZQ and TT performed a critical revision of the manuscript. All authors reviewed the results and contributed to writing of the manuscript.

## Conflict of Interest

The authors declare that the research was conducted in the absence of any commercial or financial relationships that could be construed as a potential conflict of interest.

## Footnotes

1 http://zmf.umm.uni-heidelberg.de/apps/zmf/mirwalk2/

2 https://david.ncifcrf.gov/

3 https://bioinfogp.cnb.csic.es/tools/venny/index.html

## References

[B1] Abbastabar M. Kheyrollah M. Azizian K. Bagherlou N. Tehrani S. S. Maniati M. (2018). Multiple functions of p27 in cell cycle, apoptosis, epigenetic modification and transcriptional regulation for the control of cell growth: a double-edged sword protein. *DNA Repair.* 69 63–72. 10.1016/j.dnarep.2018.07.008 30075372

[B2] Allen J. L. Oberdorster G. Morris-Schaffer K. Wong C. Klocke C. Sobolewski M. (2017). Developmental neurotoxicity of inhaled ambient ultrafine particle air pollution: parallels with neuropathological and behavioral features of autism and other neurodevelopmental disorders. *Neurotoxicology* 59 140–154. 10.1016/j.neuro.2015.12.014 26721665 PMC4917489

[B3] Andreu Z. Khan M. A. Gonzalez-Gomez P. Negueruela S. Hortiguela R. San Emeterio J. (2015). The cyclin-dependent kinase inhibitor p27 kip1 regulates radial stem cell quiescence and neurogenesis in the adult hippocampus. *Stem Cells* 33 219–229. 10.1002/stem.1832 25185890

[B4] Bailey A. R. Hou H. Song M. Obregon D. F. Portis S. Barger S. (2013). GFAP expression and social deficits in transgenic mice overexpressing human sAPPalpha. *Glia* 61 1556–1569. 10.1002/glia.22544 23840007 PMC3729742

[B5] Besson A. Dowdy S. F. Roberts J. M. (2008). CDK inhibitors: cell cycle regulators and beyond. *Dev. Cell* 14 159–169. 10.1016/j.devcel.2008.01.013 18267085

[B6] Billon N. Carlisi D. Datto M. B. van Grunsven L. A. Watt A. Wang X. F. (1999). Cooperation of Sp1 and p300 in the induction of the CDK inhibitor p21WAF1/CIP1 during NGF-mediated neuronal differentiation. *Oncogene* 18 2872–2882. 10.1038/sj.onc.1202712 10362258

[B7] Bosnjak Z. J. Logan S. Liu Y. Bai X. (2016). Recent insights into molecular mechanisms of propofol-induced developmental neurotoxicity: implications for the protective strategies. *Anesth. Analg.* 123 1286–1296. 10.1213/ANE.0000000000001544 27551735 PMC5073000

[B8] Cai S. Liu J. Shi X. Hu S. Zhao L. (2019). Allicin alleviated learning and memory deficits caused by lead exposure at developmental stage. *Life Sci.* 231:116532. 10.1016/j.lfs.2019.06.007 31170417

[B9] Cen B. Deguchi A. Weinstein I. B. (2008). Activation of protein kinase G increases the expression of p21CIP1, p27KIP1, and histidine triad protein 1 through Sp1. *Cancer Res.* 68 5355–5362. 10.1158/0008-5472.CAN-07-6869 18593937

[B10] Cheng L. C. Pastrana E. Tavazoie M. Doetsch F. (2009). miR-124 regulates adult neurogenesis in the subventricular zone stem cell niche. *Nat. Neurosci.* 12 399–408. 10.1038/nn.2294 19287386 PMC2766245

[B11] Cheng Y. C. Chiang M. C. Shih H. Y. Ma T. L. Yeh T. H. Huang Y. C. (2015). The transcription factor hairy/E(spl)-related 2 induces proliferation of neural progenitors and regulates neurogenesis and gliogenesis. *Dev. Biol.* 397 116–128. 10.1016/j.ydbio.2014.10.018 25446033

[B12] Chidambaran V. Costandi A. D’Mello A. (2015). Propofol: a review of its role in pediatric anesthesia and sedation. *CNS Drugs* 29 543–563. 10.1007/s40263-015-0259-6 26290263 PMC4554966

[B13] Cunningham J. J. Levine E. M. Zindy F. Goloubeva O. Roussel M. F. Smeyne R. J. (2002). The cyclin-dependent kinase inhibitors p19(Ink4d) and p27(Kip1) are coexpressed in select retinal cells and act cooperatively to control cell cycle exit. *Mol. Cell Neurosci.* 19 359–374. 10.1006/mcne.2001.1090 11906209

[B14] Dweep H. Gretz N. (2015). miRWalk2.0: a comprehensive atlas of microRNA-target interactions. *Nat. Methods* 12:697. 10.1038/nmeth.3485 26226356

[B15] Fan J. Zhou Q. Qin Z. Tao T. (2016). Effect of propofol on microRNA expression in rat primary embryonic neural stem cells. *BMC Anesthesiol.* 16:95 10.1186/s12871-016-0259-251 PMC5064799 27737635

[B16] Glen J. B. I. (2018). The discovery and development of propofol anesthesia: the 2018 Lasker-DeBakey clinical medical research award. *JAMA* 320 1235–1236. 10.1001/jama.2018.12756 30208399

[B17] Gu X. Fu C. Lin L. Liu S. Su X. Li A. (2018). miR-124 and miR-9 mediated downregulation of HDAC5 promotes neurite development through activating MEF2C-GPM6A pathway. *J. Cell Physiol.* 233 673–687. 10.1002/jcp.25927 28332716

[B18] Hebert S. S. De Strooper B. (2009). Alterations of the microRNA network cause neurodegenerative disease. *Trends Neurosci.* 32 199–206. 10.1016/j.tins.2008.12.003 19268374

[B19] Huang T. Liu Y. Huang M. Zhao X. Cheng L. (2010). Wnt1-cre-mediated conditional loss of Dicer results in malformation of the midbrain and cerebellum and failure of neural crest and dopaminergic differentiation in mice. *J. Mol. Cell Biol.* 2 152–163. 10.1093/jmcb/mjq008 20457670

[B20] Huang da W. Sherman B. T. Lempicki R. A. (2009). Systematic and integrative analysis of large gene lists using DAVID bioinformatics resources. *Nat. Protoc.* 4 44–57. 10.1038/nprot.2008.211 19131956

[B21] Hung C. Y. Hsu T. I. Chuang J. Y. Su T. P. Chang W. C. Hung J. J. (2020). Sp1 in astrocyte is important for neurite outgrowth and synaptogenesis. *Mol. Neurobiol.* 57 261–277. 10.1007/s12035-019-01694-7 31317491 PMC7269153

[B22] Hussaini S. M. Q. Jang M. H. (2018). New roles for old glue: astrocyte function in synaptic plasticity and neurological disorders. *Int. Neurourol. J.* 22(Suppl. 3), S106–S114. 10.5213/inj.1836214.107 30396259 PMC6234728

[B23] Johnson K. Barragan J. Bashiruddin S. Smith C. J. Tyrrell C. Parsons M. J. (2016). Gfap-positive radial glial cells are an essential progenitor population for later-born neurons and glia in the zebrafish spinal cord. *Glia* 64 1170–1189. 10.1002/glia.22990 27100776 PMC4918407

[B24] Karen T. Schlager G. W. Bendix I. Sifringer M. Herrmann R. Pantazis C. (2013). Effect of propofol in the immature rat brain on short- and long-term neurodevelopmental outcome. *PLoS One* 8:e64480. 10.1371/journal.pone.0064480 23737984 PMC3667818

[B25] Kriegstein A. Alvarez-Buylla A. (2009). The glial nature of embryonic and adult neural stem cells. *Annu. Rev. Neurosci.* 32 149–184. 10.1146/annurev.neuro.051508.135600 19555289 PMC3086722

[B26] Krzisch M. Sultan S. Sandell J. Demeter K. Vutskits L. Toni N. (2013). Propofol anesthesia impairs the maturation and survival of adult-born hippocampal neurons. *Anesthesiology* 118 602–610. 10.1097/ALN.0b013e3182815948 23314165

[B27] Lee S. Y. Yang J. Park J. H. Shin H. K. Kim W. J. Kim S. Y. (2020). The MicroRNA-92a/Sp1/MyoD axis regulates hypoxic stimulation of myogenic lineage differentiation in mouse embryonic stem cells. *Mol. Ther.* 28 142–156. 10.1016/j.ymthe.2019.08.014 31606324 PMC6951826

[B28] Li C. Xing T. Tang M. Yong W. Yan D. Deng H. (2008). Involvement of cyclin D1/CDK4 and pRb mediated by PI3K/AKT pathway activation in Pb2+ -induced neuronal death in cultured hippocampal neurons. *Toxicol. Appl. Pharmacol.* 229 351–361. 10.1016/j.taap.2008.01.039 18353414

[B29] Li Y. Liu Y. Fan J. Zhou Q. Song X. Peng Z. (2018). Validation and bioinformatic analysis of propofol-induced differentially expressed microRNAs in primary cultured neural stem cells. *Gene* 664 90–100. 10.1016/j.gene.2018.04.046 29679758

[B30] Liang C. Du F. Wang J. Cang J. Xue Z. (2019). Propofol regulates neural stem cell proliferation and differentiation via calmodulin-dependent protein kinase II/AMPK/ATF5 signaling axis. *Anesth. Analg.* 129 608–617. 10.1213/ANE.0000000000003844 30303867

[B31] Liu N. K. Xu X. M. (2011). MicroRNA in central nervous system trauma and degenerative disorders. *Physiol. Genomics* 43 571–580. 10.1152/physiolgenomics.00168.2010 21385946 PMC3110891

[B32] McCann M. E. Soriano S. G. (2019). Does general anesthesia affect neurodevelopment in infants and children? *BMJ* 367:l6459. 10.1136/bmj.l6459 31818811

[B33] Mondanizadeh M. Arefian E. Mosayebi G. Saidijam M. Khansarinejad B. Hashemi S. M. (2015). MicroRNA-124 regulates neuronal differentiation of mesenchymal stem cells by targeting Sp1 mRNA. *J. Cell Biochem.* 116 943–953. 10.1002/jcb.25045 25559917

[B34] Neo W. H. Yap K. Lee S. H. Looi L. S. Khandelia P. Neo S. X. (2014). MicroRNA miR-124 controls the choice between neuronal and astrocyte differentiation by fine-tuning Ezh2 expression. *J. Biol. Chem.* 289 20788–20801. 10.1074/jbc.M113.525493 24878960 PMC4110287

[B35] Opitz O. G. Rustgi A. K. (2000). Interaction between Sp1 and cell cycle regulatory proteins is important in transactivation of a differentiation-related gene. *Cancer Res.* 60 2825–2830. 10850422

[B36] Palazuelos J. Klingener M. Aguirre A. (2014). TGF signaling regulates the timing of CNS myelination by modulating oligodendrocyte progenitor cell cycle exit through SMAD3/4/FoxO1/Sp1. *J. Neurosci.* 34 7917–7930. 10.1523/jneurosci.0363-14.2014 24899714 PMC4044250

[B37] Pauklin S. Madrigal P. Bertero A. Vallier L. (2016). Initiation of stem cell differentiation involves cell cycle-dependent regulation of developmental genes by Cyclin D. *Genes Dev.* 30 421–433. 10.1101/gad.271452.115 26883361 PMC4762427

[B38] Qiao H. Li Y. Xu Z. Li W. Fu Z. Wang Y. (2017). Propofol affects neurodegeneration and neurogenesis by regulation of autophagy via effects on intracellular calcium homeostasis. *Anesthesiology* 127 490–501. 10.1097/ALN.0000000000001730 28614084 PMC5561483

[B39] Qiliang J. Wang Y. Shia X. (2016). Propofol inhibits neurogenesis of rat neural stem cells by upregulating microRNA-141-3p. *Stem Cells Dev.* 26 189–196. 10.1089/scd.2016.0257) 27796156

[B40] Santos M. C. Tegge A. N. Correa B. R. Mahesula S. Kohnke L. Q. Qiao M. (2016). miR-124, -128, and -137 orchestrate neural differentiation by acting on overlapping gene sets containing a highly connected transcription factor network. *Stem Cells* 34 220–232. 10.1002/stem.2204 26369286

[B41] Sanuki R. Onishi A. Koike C. Muramatsu R. Watanabe S. Muranishi Y. (2011). miR-124a is required for hippocampal axogenesis and retinal cone survival through Lhx2 suppression. *Nat. Neurosci.* 14 1125–1134. 10.1038/nn.2897 21857657

[B42] Saraiva C. Paiva J. Santos T. Ferreira L. Bernardino L. (2016). MicroRNA-124 loaded nanoparticles enhance brain repair in Parkinson’s disease. *J. Contr. Release* 235 291–305. 10.1016/j.jconrel.2016.06.005 27269730

[B43] Sun Y. Luo Z. M. Guo X. M. Su D. F. Liu X. (2015). An updated role of microRNA-124 in central nervous system disorders: a review. *Front. Cell Neurosci.* 9:193. 10.3389/fncel.2015.00193 26041995 PMC4438253

[B44] Tao T. Zhao Z. Hao L. Gu M. Chen L. Tang J. (2013). Propofol promotes proliferation of cultured adult rat hippocampal neural stem cells. *J. Neurosurg. Anesthesiol.* 25 299–305. 10.1097/ANA.0b013e31828baa93 23519370

[B45] Umezawa M. Onoda A. Korshunova I. Jensen A. C. O. Koponen I. K. Jensen K. A. (2018). Maternal inhalation of carbon black nanoparticles induces neurodevelopmental changes in mouse offspring. *Part Fibre Toxicol.* 15:36. 10.1186/s12989-018-0272-2 30201004 PMC6131790

[B46] Vizcaino C. Mansilla S. Portugal J. (2015). Sp1 transcription factor: a long-standing target in cancer chemotherapy. *Pharmacol. Ther.* 152 111–124. 10.1016/j.pharmthera.2015.05.008 25960131

[B47] Wegleiter T. Buthey K. Gonzalez-Bohorquez D. Hruzova M. Bin Imtiaz M. K. Abegg A. (2019). Palmitoylation of BMPR1a regulates neural stem cell fate. *Proc. Natl. Acad. Sci. U.S.A.* 116 25688–25696. 10.1073/pnas.1912671116 31772009 PMC6926058

[B48] Wei C. Zhang W. Zhou Q. Zhao C. Du Y. Yan Q. (2016). Mithramycin A alleviates cognitive deficits and reduces neuropathology in a transgenic mouse model of Alzheimer’s disease. *Neurochem. Res.* 41 1924–1938. 10.1007/s11064-016-1903-3 27072684

[B49] Xue Y. Ouyang K. Huang J. Zhou Y. Ouyang H. Li H. (2013). Direct conversion of fibroblasts to neurons by reprogramming PTB-regulated microRNA circuits. *Cell* 152 82–96. 10.1016/j.cell.2012.11.045 23313552 PMC3552026

[B50] Yeo S. Bandyopadhyay S. Messing A. Brenner M. (2013). Transgenic analysis of GFAP promoter elements. *Glia* 61 1488–1499. 10.1002/glia.22536 23832770 PMC4319660

[B51] Zhang S. Liang Z. Sun W. Pei L. (2017). Repeated propofol anesthesia induced downregulation of hippocampal miR-132 and learning and memory impairment of rats. *Brain Res.* 1670 156–164. 10.1016/j.brainres.2017.04.011 28465226

[B52] Zou W. W. Xiao H. P. Gu M. N. Liu K. X. Liu Z. Q. (2013). Propofol induces rat embryonic neural stem cell apoptosis by activating both extrinsic and intrinsic pathways. *Mol. Med. Rep.* 7 1123–1128. 10.3892/mmr.2013.1298 23443133

