# Supplementary material for: Propofol Exposure Disturbs the Differentiation of Rodent Neural Stem Cells via an miR-124-3p/Sp1/Cdkn1b Axis
Source: Front Cell Dev Biol. 2020 Aug 27;8:838. doi: 10.3389/fcell.2020.00838 (PMC7481336; doi:10.3389/fcell.2020.00838)
Supplement: Supplementary file 1 [file Image_1.PDF]

Supplementary Material

1 Supplementary Figure 1

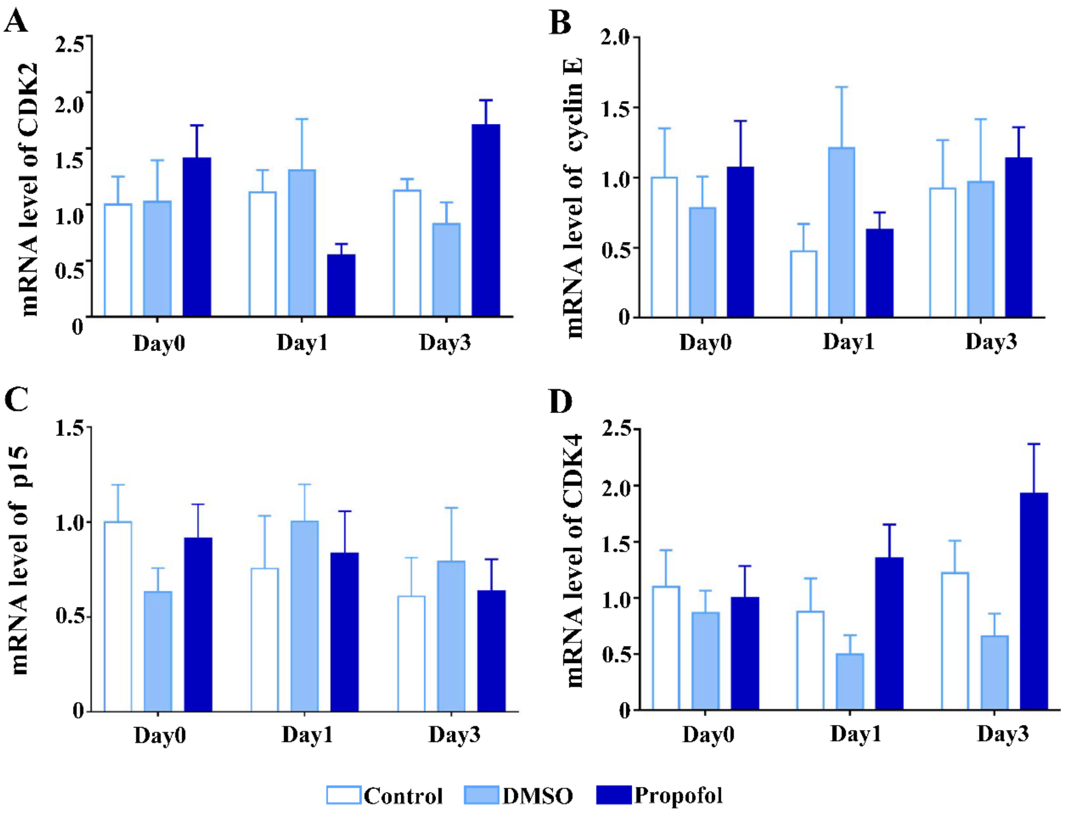

Supplementary Figure 1. qRT-PCR analysis of the mRNA levels of cell-cycle related proteins. (A-D). The results showed that the transcription levels of CDK2, cyclin E, p15 and CDK4 did not show significant differences following propofol exposure.

2 Supplementary Figure 2

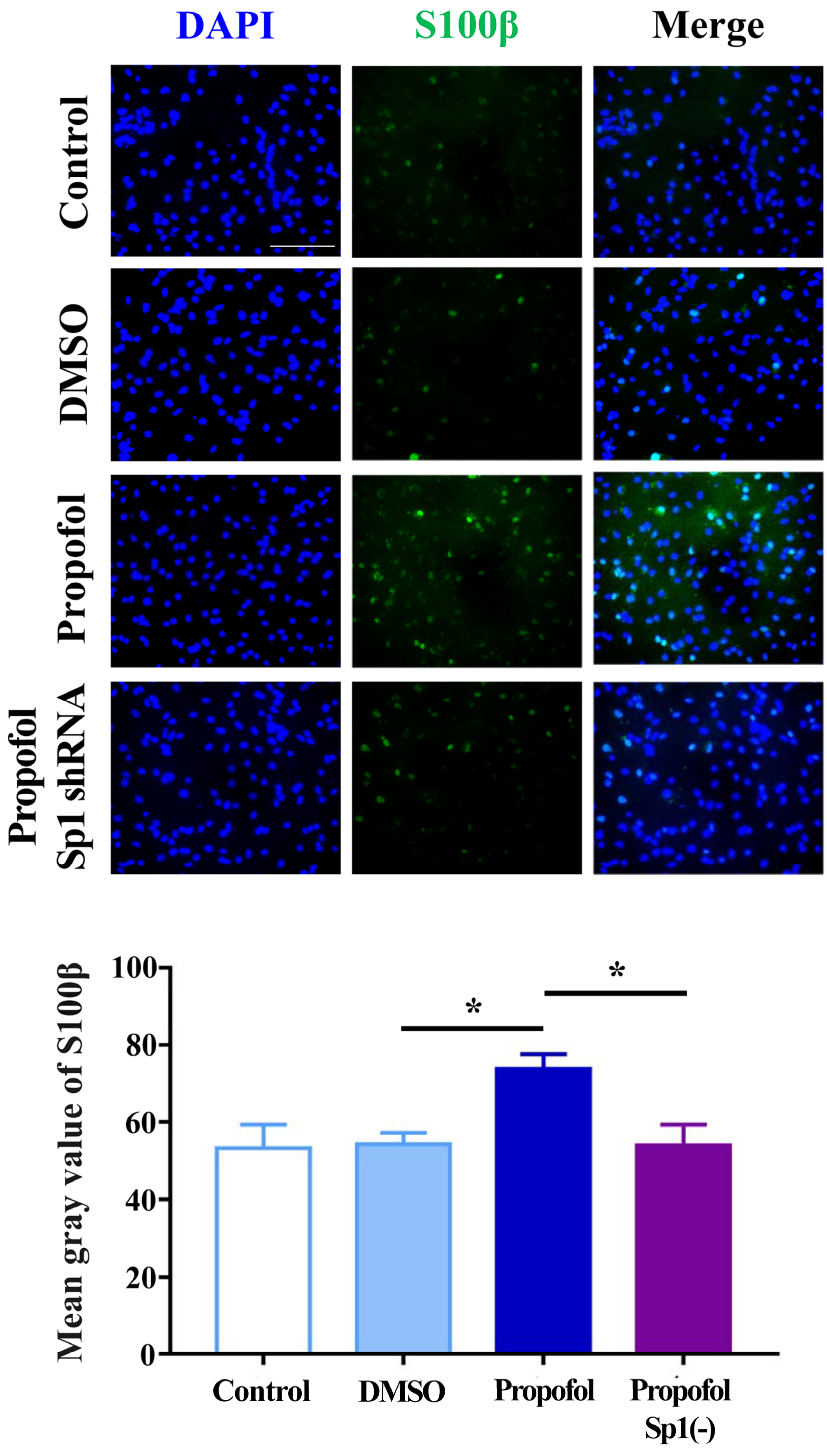

Supplementary Figure 2. Immunofluorescence of S100β in day3. The results showed that the expression of S100β increased in propofol group. * P < 0.05. Scale bar represents 100 µm.

3 Supplementary Figure 3

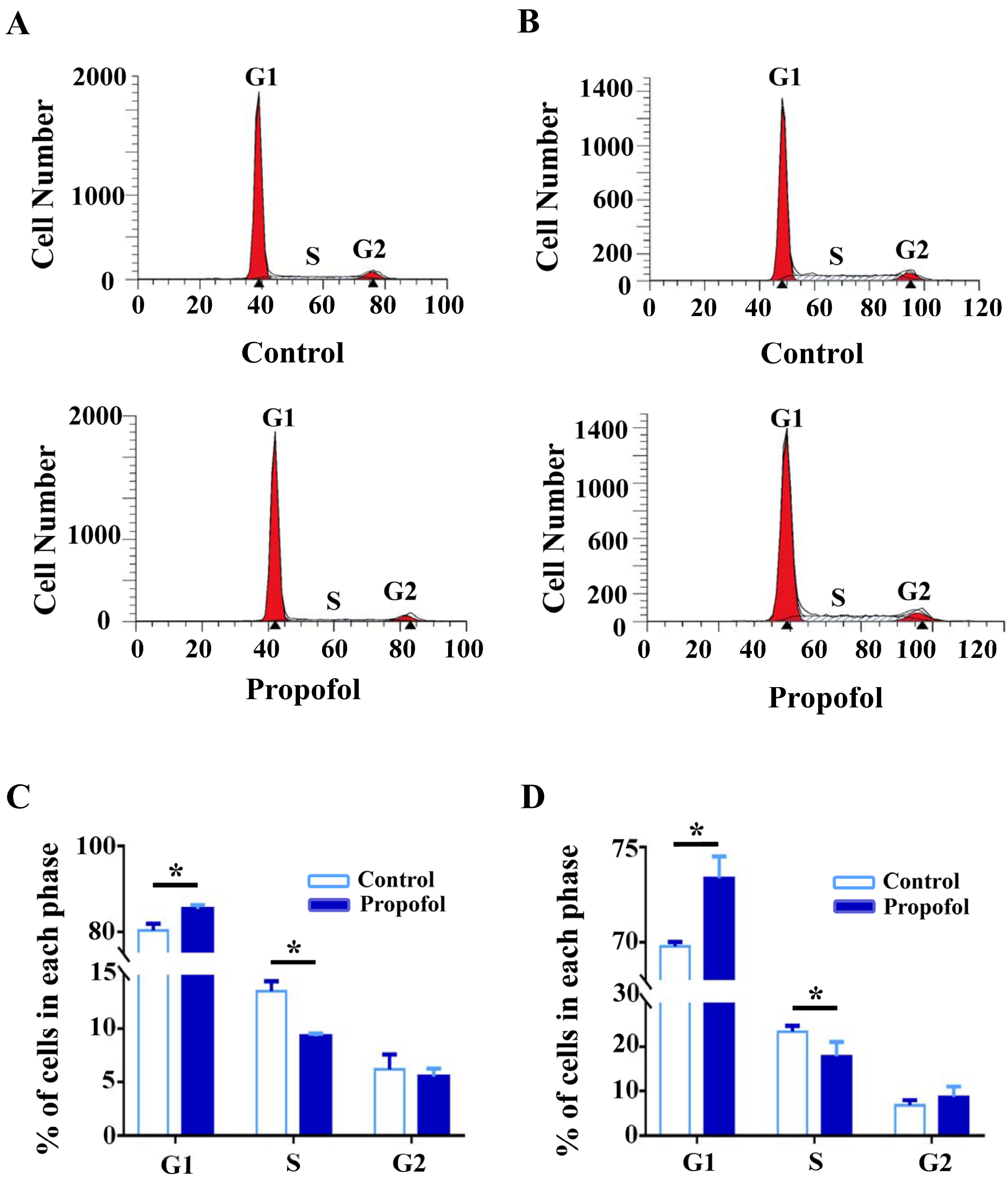

Supplementary Figure 2. Cell cycle analysis. (A, C). The percentage of cells in each phase in day1. (B, D) The percentage of cells in each phase in day3. * $P < 0.05$.

4 Supplementary Figure 4

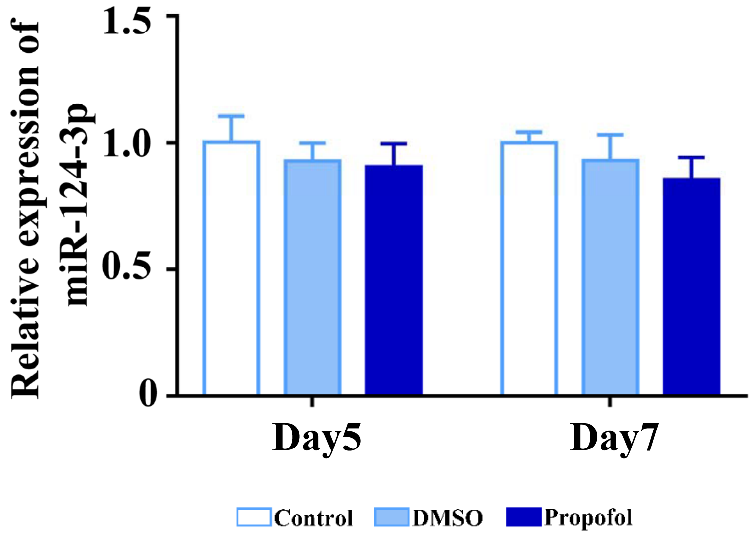

Supplementary Figure 4. qRT-PCR analysis of the relative expression of miR-124-3p. The results showed no significant differences in miR-124-3p level among groups following propofol exposure in day5 and day7.

5 Supplementary Figure 5

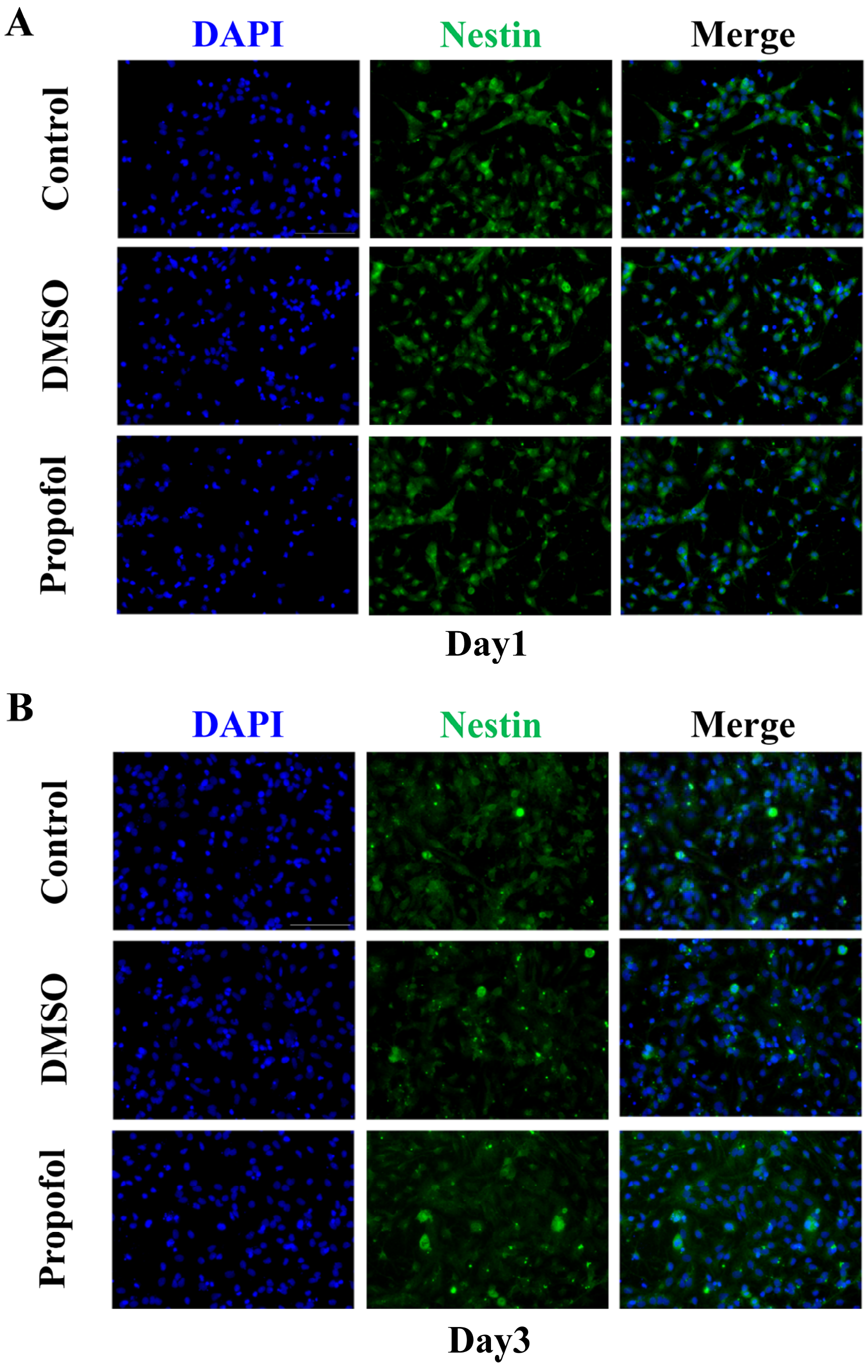

Supplementary Figure 5. Immunofluorescence of nestin in day1 and day3. (A). The percentage of nestin+ cells in day1 was over 90%. (B). The fluorescence intensity of nestin in day3 has decreased compared with day1. Scale bar represents 100 µm.
