# Supplementary material for: Propofol Exposure Disturbs the Differentiation of Rodent Neural Stem Cells via an miR-124-3p/Sp1/Cdkn1b Axis
Source: Front Cell Dev Biol. 2020 Aug 27;8:838. doi: 10.3389/fcell.2020.00838 (PMC7481336; doi:10.3389/fcell.2020.00838)
Supplement: Supplementary file 2 [file Table_1.pdf]

Supplementary Material

Genes	Sequence	Usage
miR-124-3p mimic	TAAGGCACGCGGTGAATGCC	Overexpression
miR-124-3p Forward	ACACTCCAGCTGGGTAAGGCACG	qRT-PCR
miR-124-3p Reversed	CTCAACTGGTGTCGTGGA	
Sp1 Forward	CAATACCACCCTTACACCCA	qRT-PCR
Sp1 Reversed	GCTGTTCTCTCCTTCTTCTCCA	
SP1- XhoI F	CCGCTCGAGCAGGAATCACACTGTGCCT	Luciferase
SP1- NotI R	ATAAGAATGCGGCCGCGGTTATTCCCAG TATCAAG	
Sp1 shRNA F	CCACCAATATGGTAGCCATTTCAAGAGA ATGGCTACCATATTGGTGG	Sp1 silencing
Sp1 shRNA R	CCACCAATATGGTAGCCATTCTCTTGAAA TGGCTACCATATTGGTGG	
Cdkn1b F	TTGGAGAAGCACTGCCGAGA	qRT-PCR
Cdkn1b R	CTCTCCACCTCCTGCCACTC	
Cdkn1b promoter F	TACAGCGCCCGGACCTAG	ChIP
Cdkn1b promoter R	GGTGTACGACTGCCAACAAACC	

Supplementary Table 1 Primers sequences used in current study
