# Supplementary material for: Propofol Exposure Disturbs the Differentiation of Rodent Neural Stem Cells via an miR-124-3p/Sp1/Cdkn1b Axis
Source: Front Cell Dev Biol. 2020 Aug 27;8:838. doi: 10.3389/fcell.2020.00838 (PMC7481336; doi:10.3389/fcell.2020.00838)
Supplement: Supplementary file 3 [file Table_2.pdf]

Supplementary Table 2. Prediction the target genes of miR-124-3p

miRNA	MIMATid	Gene	EntrezID	RefseqID	miRWalk	miRanda	miRDB	Targetsca	SUM
rno-miR-124-3p	MIMAT0000828	Xkr8	313033	NM_001012099	1	1	1	1	4
rno-miR-124-3p	MIMAT0000828	Ctdsp1	363249	NM_001128079	1	1	1	1	4
rno-miR-124-3p	MIMAT0000828	Wipf2	360620	NM_001191825	1	1	1	1	4
rno-miR-124-3p	MIMAT0000828	Ptpn4	246116	NM_001100479	1	1	1	1	4
rno-miR-124-3p	MIMAT0000828	Shroom4	317391	NM_001191730	1	1	1	1	4
rno-miR-124-3p	MIMAT0000828	Zfp9	100158232	NM_001127635	1	1	1	1	4
rno-miR-124-3p	MIMAT0000828	Axin1	79257	NM_024405	1	1	1	1	4
rno-miR-124-3p	MIMAT0000828	Lrrc57	311346	NM_001012354	1	1	1	1	4
rno-miR-124-3p	MIMAT0000828	9-Sep	83788	NM_001113497	1	1	1	1	4
rno-miR-124-3p	MIMAT0000828	Ptprj	29645	NM_017269	1	1	1	1	4
rno-miR-124-3p	MIMAT0000828	Arhgef1	60323	NM_021694	1	1	1	1	4
rno-miR-124-3p	MIMAT0000828	Slitrk6	290467	NM_001106057	1	1	1	1	4
rno-miR-124-3p	MIMAT0000828	Phf17	310352	NM_001107670	1	1	1	1	4
rno-miR-124-3p	MIMAT0000828	Cybrd1	295669	NM_001011954	1	1	1	1	4
rno-miR-124-3p	MIMAT0000828	Tpst2	288719	NM_001008508	1	1	1	1	4
rno-miR-124-3p	MIMAT0000828	Chic1	363484	XM_001055438	1	1	1	1	4
rno-miR-124-3p	MIMAT0000828	Prr16	361327	NM_001108432	1	1	1	1	4
rno-miR-124-3p	MIMAT0000828	Pdcd6	308061	NM_001107452	1	1	1	1	4
rno-miR-124-3p	MIMAT0000828	Aldh4a1	641316	NM_001134698	1	1	1	1	4
rno-miR-124-3p	MIMAT0000828	LOC6837	683753	XM_002728150	1	1	1	1	4
rno-miR-124-3p	MIMAT0000828	Hebp2	308632	NM_001107515	1	1	1	1	4
rno-miR-124-3p	MIMAT0000828	Fam76b	367021	NM_001108994	1	1	1	1	4
rno-miR-124-3p	MIMAT0000828	Ifit1lb	294090	XM_001079971	1	1	1	1	4
rno-miR-124-3p	MIMAT0000828	Cc2d1b	313478	XM_001067280	1	1	1	1	4
rno-miR-124-3p	MIMAT0000828	Gfpt2	360518	NM_001002819	1	1	1	1	4
rno-miR-124-3p	MIMAT0000828	Adamts12	294809	NM_001106420	1	1	1	1	4
rno-miR-124-3p	MIMAT0000828	Gpt2	307759	NM_001012057	1	1	1	1	4
rno-miR-124-3p	MIMAT0000828	Itsn2	313934	XM_233945	1	1	1	1	4
rno-miR-124-3p	MIMAT0000828	Chuk	309361	NM_001107588	1	1	1	1	4
rno-miR-124-3p	MIMAT0000828	Ccdc89	293107	NM_001134833	1	1	1	1	4
rno-miR-124-3p	MIMAT0000828	Hipk2	362342	NM_001108622	1	1	1	1	4
rno-miR-124-3p	MIMAT0000828	Cav1	25404	NM_031556	1	1	1	1	4
rno-miR-124-3p	MIMAT0000828	Sft2d2	360868	NM_001034011	1	1	1	1	4
rno-miR-124-3p	MIMAT0000828	Ube3a	361585	NM_001191837	1	1	1	1	4
rno-miR-124-3p	MIMAT0000828	Fam171a1	680259	XM_001056358	1	1	1	1	4
rno-miR-124-3p	MIMAT0000828	Mylk2	117558	NM_057209	1	1	1	1	4
rno-miR-124-3p	MIMAT0000828	Prps1	29562	NM_017243	1	1	1	1	4
rno-miR-124-3p	MIMAT0000828	Lck	313050	NM_001100709	1	1	1	1	4
rno-miR-124-3p	MIMAT0000828	Daw1	363267	NM_001025025	1	1	1	1	4
rno-miR-124-3p	MIMAT0000828	Ctdspl	301056	NM_001106865	1	1	1	1	4
rno-miR-124-3p	MIMAT0000828	Cdh2	83501	NM_031333	1	1	1	1	4
rno-miR-124-3p	MIMAT0000828	Usp30	304579	NM_001107153	1	1	1	1	4
rno-miR-124-3p	MIMAT0000828	Fmr1	24948	NM_052804	1	1	1	1	4
rno-miR-124-3p	MIMAT0000828	Tmod1	25566	NM_013044	1	1	1	1	4
rno-miR-124-3p	MIMAT0000828	Vat1	287721	NM_001033683	1	1	1	1	4
rno-miR-124-3p	MIMAT0000828	9-Sep	83788	NM_031837	1	1	1	1	4
rno-miR-124-3p	MIMAT0000828	Rela	309165	NM_199267	1	1	1	1	4
rno-miR-124-3p	MIMAT0000828	RGD1306	316406	NM_001108219	1	1	1	1	4
rno-miR-124-3p	MIMAT0000828	Chic1	363484	XM_001056014	1	1	1	1	4
rno-miR-124-3p	MIMAT0000828	Smox	308652	NM_001134854	1	1	1	1	4
rno-miR-124-3p	MIMAT0000828	Slitrk4	302473	NM_001106947	1	1	1	1	4
rno-miR-124-3p	MIMAT0000828	Ppfibp2	308918	NM_001100582	1	1	1	1	4
rno-miR-124-3p	MIMAT0000828	Ascc2	498402	NM_001109091	1	1	1	1	4
rno-miR-124-3p	MIMAT0000828	Hivep1	117140	NM_001105751	1	1	1	1	4
rno-miR-124-3p	MIMAT0000828	Eps8	312812	XM_001072957	1	1	1	1	4
rno-miR-124-3p	MIMAT0000828	Manbal	499934	NM_001173380	1	1	1	1	4
rno-miR-124-3p	MIMAT0000828	Cav1	25404	NM_133651	1	1	1	1	4
rno-miR-124-3p	MIMAT0000828	Ifit1lb	294090	XM_220058	1	1	1	1	4
rno-miR-124-3p	MIMAT0000828	Gmcl1	312516	NM_001033931	1	1	1	1	4
rno-miR-124-3p	MIMAT0000828	Iqgap1	361598	NM_001108489	1	1	1	1	4
rno-miR-124-3p	MIMAT0000828	Irf1	24508	NM_012591	1	1	1	1	4
rno-miR-124-3p	MIMAT0000828	Itpr3	25679	NM_013138	1	1	1	1	4
rno-miR-124-3p	MIMAT0000828	Dapk1	306722	NM_001107335	1	1	1	1	4
rno-miR-124-3p	MIMAT0000828	Myd88	301059	NM_198130	1	1	1	1	4
rno-miR-124-3p	MIMAT0000828	Rnf135	303350	NM_001012010	1	1	1	1	4
rno-miR-124-3p	MIMAT0000828	Lca5l	498065	NM_001017484	1	1	1	1	4

rno-miR-124-3p	MIMAT0000828	Ogfod3	303749	NM_001013976	1	1	1	1	4
rno-miR-124-3p	MIMAT0000828	9-Sep	83788	NM_176856	1	1	1	1	4
rno-miR-124-3p	MIMAT0000828	Arrdc2	306344	NM_001107303	1	1	1	1	4
rno-miR-124-3p	MIMAT0000828	Anxa11	290527	NM_001011918	1	1	1	1	4
rno-miR-124-3p	MIMAT0000828	Tst	25274	NM_012808	1	1	1	1	4
rno-miR-124-3p	MIMAT0000828	Rnf114	362277	NM_001001517	1	1	1	1	4
rno-miR-124-3p	MIMAT0000828	Plxnb2	315217	NM_001108106	1	1	1	1	4
rno-miR-124-3p	MIMAT0000828	Wtap	499020	NM_001113543	1	1	1	1	4
rno-miR-124-3p	MIMAT0000828	Zfp192	306974	NM_001100574	1	1	1	1	4
rno-miR-124-3p	MIMAT0000828	Rassf5	54355	NM_019365	1	1	1	1	4
rno-miR-124-3p	MIMAT0000828	Tex30	301381	NM_001106909	1	1	1	1	4
rno-miR-124-3p	MIMAT0000828	LOC6544	654482	NM_001039174	1	1	1	1	4
rno-miR-124-3p	MIMAT0000828	Cbs	24250	NM_012522	1	1	1	1	4
rno-miR-124-3p	MIMAT0000828	Nipa1	308668	NM_001107519	1	1	1	1	4
rno-miR-124-3p	MIMAT0000828	Plp2	302562	NM_207601	1	1	1	1	4
rno-miR-124-3p	MIMAT0000828	Rufy1	360521	NM_001100727	1	1	1	1	4
rno-miR-124-3p	MIMAT0000828	Sgk1	29517	NM_001193568	1	1	1	1	4
rno-miR-124-3p	MIMAT0000828	Eps8	312812	XM_232499	1	1	1	1	4
rno-miR-124-3p	MIMAT0000828	Zcchc24	361104	NM_001108394	1	1	1	1	4
rno-miR-124-3p	MIMAT0000828	Serinc2	313057	NM_001031656	1	1	1	1	4
rno-miR-124-3p	MIMAT0000828	Vim	81818	NM_031140	1	1	1	1	4
rno-miR-124-3p	MIMAT0000828	Mybl2	296344	NM_001106536	1	1	1	1	4
rno-miR-124-3p	MIMAT0000828	Jag1	29146	NM_019147	1	1	1	1	4
rno-miR-124-3p	MIMAT0000828	Fxr2	287433	NM_001100647	1	1	1	1	4
rno-miR-124-3p	MIMAT0000828	Papss2	294103	NM_001106375	1	1	1	1	4
rno-miR-124-3p	MIMAT0000828	Ssfa2	311146	NM_001107738	1	1	1	1	4
rno-miR-124-3p	MIMAT0000828	Itgb1	24511	NM_017022	1	1	1	1	4
rno-miR-124-3p	MIMAT0000828	Pcsk1	25204	NM_017091	1	1	1	1	4
rno-miR-124-3p	MIMAT0000828	Cyyr1	304138	NM_001013980	1	1	1	1	4
rno-miR-124-3p	MIMAT0000828	Slc31a2	298091	NM_001033693	1	1	1	1	4
rno-miR-124-3p	MIMAT0000828	Ror2	306782	NM_001107339	1	1	1	1	4
rno-miR-124-3p	MIMAT0000828	Srek1ip1	361888	NM_001008373	1	1	1	1	4
rno-miR-124-3p	MIMAT0000828	G3bp1	171092	NM_133565	1	1	1	1	4
rno-miR-124-3p	MIMAT0000828	Tor1aip2	304881	NM_199100	1	1	1	1	4
rno-miR-124-3p	MIMAT0000828	Dgat2	252900	NM_001012345	1	1	1	1	4
rno-miR-124-3p	MIMAT0000828	Hivep2	29721	NM_024137	1	1	1	1	4
rno-miR-124-3p	MIMAT0000828	Sdf2l1	680945	NM_001109433	1	1	1	1	4
rno-miR-124-3p	MIMAT0000828	Chdh	290551	NM_198731	1	1	1	1	4
rno-miR-124-3p	MIMAT0000828	Frmd8	309172	NM_001008348	1	1	1	1	4
rno-miR-124-3p	MIMAT0000828	Naa15	310399	NM_001107674	1	1	1	1	4
rno-miR-124-3p	MIMAT0000828	Tmem109	361732	NM_001007736	1	1	1	1	4
rno-miR-124-3p	MIMAT0000828	Tnrc6b	192178	NM_138845	1	1	1	1	4
rno-miR-124-3p	MIMAT0000828	Ubxn2a	685859	NM_001109482	1	1	1	1	4
rno-miR-124-3p	MIMAT0000828	Wtap	499020	NM_001113544	1	1	1	1	4
rno-miR-124-3p	MIMAT0000828	Slc27a1	94172	NM_053580	1	1	1	1	4
rno-miR-124-3p	MIMAT0000828	Zkscan3	306977	NM_001012053	1	1	1	1	4
rno-miR-124-3p	MIMAT0000828	Ginm1	361448	XM_001059157	1	1	1	1	4
rno-miR-124-3p	MIMAT0000828	RGD1311	289399	NM_001134502	1	1	1	1	4
rno-miR-124-3p	MIMAT0000828	Ptpn12	117255	NM_057115	1	1	1	1	4
rno-miR-124-3p	MIMAT0000828	Slc5a12	362188	NM_001108588	1	1	1	1	4
rno-miR-124-3p	MIMAT0000828	Mtr	81522	NM_030864	1	1	1	1	4
rno-miR-124-3p	MIMAT0000828	Sgk1	29517	NM_001193569	1	1	1	1	4
rno-miR-124-3p	MIMAT0000828	Tjp2	115769	NM_053773	1	1	1	1	4
rno-miR-124-3p	MIMAT0000828	Tmco3	306607	NM_001135857	1	1	1	1	4
rno-miR-124-3p	MIMAT0000828	Slc9a2	24783	NM_001113335	1	1	1	1	4
rno-miR-124-3p	MIMAT0000828	Pkn2	207122	NM_001105755	1	1	1	1	4
rno-miR-124-3p	MIMAT0000828	Mpp6	362359	NM_001134982	1	1	1	1	4
rno-miR-124-3p	MIMAT0000828	Oaf	315594	NM_001014090	1	1	1	1	4
rno-miR-124-3p	MIMAT0000828	Dlx5	25431	NM_012943	1	1	1	1	4
rno-miR-124-3p	MIMAT0000828	Slc30a7	310801	NM_001191715	1	1	1	1	4
rno-miR-124-3p	MIMAT0000828	Ptbp3	83515	NM_031346	1	1	1	1	4
rno-miR-124-3p	MIMAT0000828	Clic2	294141	NM_001009651	1	1	1	1	4
rno-miR-124-3p	MIMAT0000828	Crcp	114205	NM_053670	1	1	1	1	4
rno-miR-124-3p	MIMAT0000828	Flnb	306204	NM_001107288	1	1	1	1	4
rno-miR-124-3p	MIMAT0000828	Gria2	29627	NM_001083811	1	1	1	1	4
rno-miR-124-3p	MIMAT0000828	Il6st	25205	NM_001008725	1	1	1	1	4
rno-miR-124-3p	MIMAT0000828	Alg2	313231	NM_001100710	1	1	1	1	4
rno-miR-124-3p	MIMAT0000828	Cdcp1	301082	NM_001106869	1	1	1	1	4
rno-miR-124-3p	MIMAT0000828	Myrf	293736	NM_001170487	1	1	1	1	4

rno-miR-124-3p	MIMAT0000828	Lrrc1	367113	NM_001014268	1	1	1	1	4
rno-miR-124-3p	MIMAT0000828	Myh10	79433	NM_031520	1	1	1	1	4
rno-miR-124-3p	MIMAT0000828	Tor3a	304884	NM_001009683	1	1	1	1	4
rno-miR-124-3p	MIMAT0000828	Rab38	252916	NM_145774	1	1	1	1	4
rno-miR-124-3p	MIMAT0000828	Csgalnact	306375	NM_001107309	1	1	1	1	4
rno-miR-124-3p	MIMAT0000828	Itsn2	313934	XM_001067254	1	1	1	1	4
rno-miR-124-3p	MIMAT0000828	Chsy1	292999	NM_001106268	1	1	1	1	4
rno-miR-124-3p	MIMAT0000828	RGD1359	361740	NM_001007737	1	1	1	1	4
rno-miR-124-3p	MIMAT0000828	Rbm24	690139	NM_001191100	1	1	1	1	4
rno-miR-124-3p	MIMAT0000828	Hadh	113965	NM_057186	1	1	1	1	4
rno-miR-124-3p	MIMAT0000828	Ginm1	361448	XM_341726	1	1	1	1	4
rno-miR-124-3p	MIMAT0000828	Apln	58812	NM_031612	1	1	1	1	4
rno-miR-124-3p	MIMAT0000828	Clmn	299285	NM_001106755	1	1	1	1	4
rno-miR-124-3p	MIMAT0000828	Gnai3	25643	NM_013106	1	1	1	1	4
rno-miR-124-3p	MIMAT0000828	Sgk1	29517	NM_019232	1	1	1	1	4
rno-miR-124-3p	MIMAT0000828	Vps37c	308178	NM_001107463	1	1	1	1	4
rno-miR-124-3p	MIMAT0000828	Slc9a2	24783	NM_012653	1	1	1	1	4
rno-miR-124-3p	MIMAT0000828	Steap3	170824	NM_133314	1	1	1	1	4
rno-miR-124-3p	MIMAT0000828	Rsph1	361818	NM_001012176	1	1	1	1	4
rno-miR-124-3p	MIMAT0000828	Kcnk10	65272	NM_023096	1	1	1	1	4
rno-miR-124-3p	MIMAT0000828	Ccdc130	304656	NM_001037644	1	1	1	1	4
rno-miR-124-3p	MIMAT0000828	Gria2	29627	NM_017261	1	1	1	1	4
rno-miR-124-3p	MIMAT0000828	RGD1305	313699	NM_001135781	1	1	1	1	4
rno-miR-124-3p	MIMAT0000828	Rsu1	680419	NM_001109404	1	1	1	1	4
rno-miR-124-3p	MIMAT0000828	Rinl	292760	NM_001106241	1	1	1	1	4
rno-miR-124-3p	MIMAT0000828	Nme4	685679	NM_001109478	1	1	1	1	4
rno-miR-124-3p	MIMAT0000828	Twsg1	363294	NM_001108811	1	1	1	1	4
rno-miR-124-3p	MIMAT0000828	Galnt12	313233	XM_001066416	1	1	1	1	4
rno-miR-124-3p	MIMAT0000828	Chp1	64152	NM_024139	1	1	1	1	4
rno-miR-124-3p	MIMAT0000828	L2hgdh	314196	NM_001108028	1	1	1	1	4
rno-miR-124-3p	MIMAT0000828	Ythdf3	361920	NM_001108546	1	1	1	1	4
rno-miR-124-3p	MIMAT0000828	Sema6d	311384	NM_001107768	1	1	1	1	4
rno-miR-124-3p	MIMAT0000828	Chic2	83835	NM_001105736	1	1	1	1	4
rno-miR-124-3p	MIMAT0000828	Tdg	114521	NM_053729	1	1	1	1	4
rno-miR-124-3p	MIMAT0000828	Tnfrsf26	361685	NM_001108511	1	1	1	1	4
rno-miR-124-3p	MIMAT0000828	Itsn2	313934	XM_002730131	1	1	1	1	4
rno-miR-124-3p	MIMAT0000828	Pde4b	24626	NM_017031	1	1	1	1	4
rno-miR-124-3p	MIMAT0000828	LOC1003	100361585	XM_002726532	1	1	1	1	4
rno-miR-124-3p	MIMAT0000828	Ppil3	301432	NM_175707	1	1	1	1	4
rno-miR-124-3p	MIMAT0000828	Pabpc6	292295	NM_001106208	1	1	1	1	4
rno-miR-124-3p	MIMAT0000828	Sertad2	498423	NM_001024903	1	1	1	1	4
rno-miR-124-3p	MIMAT0000828	Mgst1	171341	NM_134349	1	1	1	1	4
rno-miR-124-3p	MIMAT0000828	Sgpp1	81536	XM_001080791	1	1	1	1	4
rno-miR-124-3p	MIMAT0000828	Trpv5	116469	NM_053787	1	1	1	1	4
rno-miR-124-3p	MIMAT0000828	Slc9a3	24784	NM_012654	1	1	1	1	4
rno-miR-124-3p	MIMAT0000828	Gga2	293455	NM_001100519	1	1	1	1	4
rno-miR-124-3p	MIMAT0000828	Cdh9	29163	NM_001168630	1	1	1	1	4
rno-miR-124-3p	MIMAT0000828	Pskh1	364993	NM_001108897	1	1	1	1	4
rno-miR-124-3p	MIMAT0000828	Gria3	29628	NM_001112742	1	1	1	1	4
rno-miR-124-3p	MIMAT0000828	Cml1	59300	NM_021668	1	1	1	1	4
rno-miR-124-3p	MIMAT0000828	Pip4k2c	140607	NM_080480	1	1	1	1	4
rno-miR-124-3p	MIMAT0000828	Mapk14	81649	NM_031020	1	1	1	1	4
rno-miR-124-3p	MIMAT0000828	Ppp1r3d	689995	NM_001109564	1	1	1	1	4
rno-miR-124-3p	MIMAT0000828	Tgfbr3	29610	NM_017256	1	1	1	1	4
rno-miR-124-3p	MIMAT0000828	Galnt12	313233	XM_232988	1	1	1	1	4
rno-miR-124-3p	MIMAT0000828	Fchsd2	308864	NM_001107539	1	1	1	1	4
rno-miR-124-3p	MIMAT0000828	Pvrl4	498281	NM_001109076	1	1	1	1	4
rno-miR-124-3p	MIMAT0000828	Sppl2a	311401	NM_001107770	1	1	1	1	4
rno-miR-124-3p	MIMAT0000828	Ankrd66	501105	XM_001068682	1	1	1	1	4
rno-miR-124-3p	MIMAT0000828	Cacul1	365493	NM_001014248	1	1	1	1	4
rno-miR-124-3p	MIMAT0000828	Pnpt1	360992	NM_001142371	1	1	1	1	4
rno-miR-124-3p	MIMAT0000828	Dsg2	307562	XM_001054396	1	1	1	1	4
rno-miR-124-3p	MIMAT0000828	Itsn2	313934	XM_002730132	1	1	1	1	4
rno-miR-124-3p	MIMAT0000828	LOC1003	100361585	XM_002729479	1	1	1	1	4
rno-miR-124-3p	MIMAT0000828	Cutc	361760	NM_001108525	1	1	1	1	4
rno-miR-124-3p	MIMAT0000828	Otud7b	310677	NM_001107697	1	1	1	1	4
rno-miR-124-3p	MIMAT0000828	Pgf	94203	NM_053595	1	1	1	1	4
rno-miR-124-3p	MIMAT0000828	Tshz1	307217	XM_001060440	1	1	1	1	4
rno-miR-124-3p	MIMAT0000828	Sfxn1	364678	NM_001012213	1	1	1	1	4

rno-miR-124-3p	MIMAT0000828	Nr3c1	24413	NM_012576	1	1	1	1	4
rno-miR-124-3p	MIMAT0000828	LOC1003	100360412	XM_002728514	1	1	1	1	4
rno-miR-124-3p	MIMAT0000828	Sgpp1	81536	XM_343081	1	1	1	1	4
rno-miR-124-3p	MIMAT0000828	Vamp3	29528	NM_057097	1	1	1	1	4
rno-miR-124-3p	MIMAT0000828	Chodl	288289	NM_001105894	1	1	1	1	4
rno-miR-124-3p	MIMAT0000828	Fa2h	307855	NM_001135583	1	1	1	1	4
rno-miR-124-3p	MIMAT0000828	Col4a1	290905	NM_001135009	1	1	1	1	4
rno-miR-124-3p	MIMAT0000828	Fam105b	100362554	XM_002725921	1	1	1	1	4
rno-miR-124-3p	MIMAT0000828	Man2a1	25478	NM_012979	1	1	1	1	4
rno-miR-124-3p	MIMAT0000828	Tfcp2l1	304741	NM_001107170	1	1	1	1	4
rno-miR-124-3p	MIMAT0000828	Mst4	317589	NM_001191736	1	1	1	1	4
rno-miR-124-3p	MIMAT0000828	Cd164	83689	NM_031812	1	1	1	1	4
rno-miR-124-3p	MIMAT0000828	Yme1l1	114217	NM_053682	1	1	1	1	4
rno-miR-124-3p	MIMAT0000828	Mob1b	360920	NM_001108357	1	1	1	1	4
rno-miR-124-3p	MIMAT0000828	Pik3c2a	361632	NM_001108500	1	1	1	1	4
rno-miR-124-3p	MIMAT0000828	Gria3	29628	NM_032990	1	1	1	1	4
rno-miR-124-3p	MIMAT0000828	Tfeb	316214	NM_001025707	1	1	1	1	4
rno-miR-124-3p	MIMAT0000828	Scn7a	64155	NM_031686	1	1	1	1	4
rno-miR-124-3p	MIMAT0000828	Enpp4	301261	NM_001106892	1	1	1	1	4
rno-miR-124-3p	MIMAT0000828	Kat7	303470	NM_181081	1	1	1	1	4
rno-miR-124-3p	MIMAT0000828	Trim39	309591	NM_213562	1	1	1	1	4
rno-miR-124-3p	MIMAT0000828	Fxr1	361927	NM_001012179	1	1	1	1	4
rno-miR-124-3p	MIMAT0000828	Art1	308873	NM_001107541	1	1	1	1	4
rno-miR-124-3p	MIMAT0000828	Cnep1r1	291914	NM_001106173	1	1	1	1	4
rno-miR-124-3p	MIMAT0000828	Ptprz1	25613	NM_013080	1	1	1	1	4
rno-miR-124-3p	MIMAT0000828	Prrg4	499847	NM_001109203	1	1	1	1	4
rno-miR-124-3p	MIMAT0000828	Slc35a4	257647	NM_147140	1	1	1	1	4
rno-miR-124-3p	MIMAT0000828	Gcom1	363091	NM_001014211	1	1	1	1	4
rno-miR-124-3p	MIMAT0000828	Dsg2	307562	XM_226112	1	1	1	1	4
rno-miR-124-3p	MIMAT0000828	Nudcd2	287199	NM_001009621	1	1	1	1	4
rno-miR-124-3p	MIMAT0000828	Tshz1	307217	XM_225688	1	1	1	1	4
rno-miR-124-3p	MIMAT0000828	Vps4b	360834	NM_001025716	1	1	1	1	4
rno-miR-124-3p	MIMAT0000828	Zbtb11	304010	NM_001107097	1	1	1	1	4
rno-miR-124-3p	MIMAT0000828	Grsf1	305256	NM_001100890	1	1	1	1	4
rno-miR-124-3p	MIMAT0000828	Rab34	360571	NM_001012140	1	1	1	1	4
rno-miR-124-3p	MIMAT0000828	Slc50a1	295245	NM_001106445	1	1	1	1	4
rno-miR-124-3p	MIMAT0000828	Ttc7a	362696	NM_001100756	1	1	1	1	4
rno-miR-124-3p	MIMAT0000828	Slc29a1	63997	NM_031684	1	1	1	1	4
rno-miR-124-3p	MIMAT0000828	Ovca2	497954	NM_001109036	1	1	1	1	4
rno-miR-124-3p	MIMAT0000828	Ddx3x	317335	NM_001108246	1	1	1	1	4
rno-miR-124-3p	MIMAT0000828	Vps35	25479	NM_001105718	1	1	1	1	4
rno-miR-124-3p	MIMAT0000828	Smarcad1	312398	NM_001107864	1	1	1	1	4
rno-miR-124-3p	MIMAT0000828	Pgrmc2	361940	NM_001008374	1	1	1	1	4
rno-miR-124-3p	MIMAT0000828	Slc15a4	246280	NM_144758	1	1	1	1	4
rno-miR-124-3p	MIMAT0000828	Traf6	311245	NM_001107754	1	1	1	1	4
rno-miR-124-3p	MIMAT0000828	Zfp36l1	29344	NM_017172	1	1	1	1	4
rno-miR-124-3p	MIMAT0000828	Stt3a	500972	NM_001134749	1	1	1	1	4
rno-miR-124-3p	MIMAT0000828	Ankrd66	501105	XM_576520	1	1	1	1	4
rno-miR-124-3p	MIMAT0000828	Rab3d	140665	NM_080580	1	1	1	1	4
rno-miR-124-3p	MIMAT0000828	Tmem134	361695	NM_001078647	1	1	1	1	4
rno-miR-124-3p	MIMAT0000828	Tor1b	311854	NM_001039197	1	1	1	1	4
rno-miR-124-3p	MIMAT0000828	Selrc1	298377	NM_001106674	1	1	1	1	4
rno-miR-124-3p	MIMAT0000828	Traf3	362788	NM_001108724	1	1	1	1	4
rno-miR-124-3p	MIMAT0000828	Usp1	313387	NM_001015015	1	1	1	1	4
rno-miR-124-3p	MIMAT0000828	Cd151	64315	NM_022523	1	1	1	1	4
rno-miR-124-3p	MIMAT0000828	Rhog	308875	NM_001037195	1	1	1	1	4
rno-miR-124-3p	MIMAT0000828	RGD1309	315891	NM_001134472	1	1	1	1	4
rno-miR-124-3p	MIMAT0000828	Kif26b	305012	NM_001109079	1	1	1	1	4
rno-miR-124-3p	MIMAT0000828	Slc17a5	363103	NM_001009713	1	1	1	1	4
rno-miR-124-3p	MIMAT0000828	Mtmr10	309255	NM_001100846	1	1	1	1	4
rno-miR-124-3p	MIMAT0000828	Nfatc1	100361818	NM_001244933	1	1	1	1	4
rno-miR-124-3p	MIMAT0000828	Fam57a	100360533	XM_002727796	1	1	1	1	4
rno-miR-124-3p	MIMAT0000828	Snap23	64630	NM_022689	1	1	1	1	4
rno-miR-124-3p	MIMAT0000828	Ptpn9	266611	NM_001013040	1	1	1	1	4
rno-miR-124-3p	MIMAT0000828	Nsun2	361191	NM_001108403	1	1	1	1	4
rno-miR-124-3p	MIMAT0000828	Cyb5a	64001	NM_022245	1	1	1	1	4
rno-miR-124-3p	MIMAT0000828	Kcnk2	170899	NM_172042	1	1	1	1	4
rno-miR-124-3p	MIMAT0000828	Polr1b	83582	NM_031773	1	1	1	1	4
rno-miR-124-3p	MIMAT0000828	Prpf38b	499691	NM_001024305	1	1	1	1	4

rno-miR-124-3p	MIMAT0000828	Ddx6	500988	NM_001109292	1	1	1	1	4
rno-miR-124-3p	MIMAT0000828	Wfikkn2	287631	XM_220855	1	1	1	1	4
rno-miR-124-3p	MIMAT0000828	Tmem134	361695	NM_001078648	1	1	1	1	4
rno-miR-124-3p	MIMAT0000828	Six4	299138	NM_001106739	1	1	1	1	4
rno-miR-124-3p	MIMAT0000828	Slc16a1	25027	NM_012716	1	1	1	1	4
rno-miR-124-3p	MIMAT0000828	Serp1	80881	NM_030835	1	1	1	1	4
rno-miR-124-3p	MIMAT0000828	Tmed1	315461	NM_001013432	1	1	1	1	4
rno-miR-124-3p	MIMAT0000828	Vamp4	364033	NM_001108856	1	1	1	1	4
rno-miR-124-3p	MIMAT0000828	Nrg1	112400	NM_031588	1	1	1	1	4
rno-miR-124-3p	MIMAT0000828	Tubb4a	29213	NM_080882	1	1	1	1	4
rno-miR-124-3p	MIMAT0000828	Eea1	314764	NM_001108086	1	1	1	1	4
rno-miR-124-3p	MIMAT0000828	Rad17	310034	NM_001024778	1	1	1	1	4
rno-miR-124-3p	MIMAT0000828	Ryr2	689560	NM_001191043	1	1	1	1	4
rno-miR-124-3p	MIMAT0000828	Arpc1b	54227	NM_019289	1	1	1	1	4
rno-miR-124-3p	MIMAT0000828	Kank1	309429	NM_001037197	1	1	1	1	4
rno-miR-124-3p	MIMAT0000828	Fam117a	497983	NM_001109039	1	1	1	1	4
rno-miR-124-3p	MIMAT0000828	Myo1e	25484	NM_173101	1	1	1	1	4
rno-miR-124-3p	MIMAT0000828	Myocd	246297	NM_182667	1	1	1	1	4
rno-miR-124-3p	MIMAT0000828	Cbx2	303730	NM_001107071	1	1	1	1	4
rno-miR-124-3p	MIMAT0000828	Prtg	315806	NM_001037651	1	1	1	1	4
rno-miR-124-3p	MIMAT0000828	Flot2	83764	NM_031830	1	1	1	1	4
rno-miR-124-3p	MIMAT0000828	Twist2	59327	NM_021691	1	1	1	1	4
rno-miR-124-3p	MIMAT0000828	Slit1	65047	NM_022953	1	1	1	1	4
rno-miR-124-3p	MIMAT0000828	LOC6837	683753	XM_001067334	1	1	1	1	4
rno-miR-124-3p	MIMAT0000828	Atp1a1	24211	NM_012504	1	1	1	1	4
rno-miR-124-3p	MIMAT0000828	Lamc1	117036	NM_053966	1	1	1	1	4
rno-miR-124-3p	MIMAT0000828	Fam129b	362115	NM_001109885	1	1	1	1	4
rno-miR-124-3p	MIMAT0000828	Itga7	81008	NM_030842	1	1	1	1	4
rno-miR-124-3p	MIMAT0000828	Wipf3	259242	NM_147211	1	1	1	1	4
rno-miR-124-3p	MIMAT0000828	Ppp1r3b	192280	NM_138912	1	1	1	1	4
rno-miR-124-3p	MIMAT0000828	Cys1	690489	NM_001109597	1	1	1	1	4
rno-miR-124-3p	MIMAT0000828	Acss1	296259	NM_001106524	1	1	1	1	4
rno-miR-124-3p	MIMAT0000828	Gramd3	307288	NM_001014011	1	1	1	1	4
rno-miR-124-3p	MIMAT0000828	Myo1d	25485	NM_012983	1	1	0	1	3
rno-miR-124-3p	MIMAT0000828	Rab11fip5	312502	XM_003749791	1	1	0	1	3
rno-miR-124-3p	MIMAT0000828	LOC1009	100911248	XM_003751095	1	1	0	1	3
rno-miR-124-3p	MIMAT0000828	Suv39h2	364785	NM_001108883	1	1	0	1	3
rno-miR-124-3p	MIMAT0000828	Vcan	114122	NM_001170558	1	1	0	1	3
rno-miR-124-3p	MIMAT0000828	Slitrk5	306152	NM_001107284	1	1	0	1	3
rno-miR-124-3p	MIMAT0000828	Fbxo38	307390	XM_003751794	1	1	0	1	3
rno-miR-124-3p	MIMAT0000828	Siglech	361584	XM_001054672	1	1	0	1	3
rno-miR-124-3p	MIMAT0000828	Sult4a1	58953	NM_031641	1	1	0	1	3
rno-miR-124-3p	MIMAT0000828	Pdik1l	313609	NM_001107984	1	1	0	1	3
rno-miR-124-3p	MIMAT0000828	Abca3	302973	XM_003750787	1	1	0	1	3
rno-miR-124-3p	MIMAT0000828	Acss3	314800	NM_001108091	1	1	0	1	3
rno-miR-124-3p	MIMAT0000828	Tada2b	289717	NM_001170455	1	1	0	1	3
rno-miR-124-3p	MIMAT0000828	Il9r	24500	NM_017021	1	1	0	1	3
rno-miR-124-3p	MIMAT0000828	Pcdha1	393085	NM_199503	1	1	0	1	3
rno-miR-124-3p	MIMAT0000828	Ccl22	117551	NM_057203	1	1	0	1	3
rno-miR-124-3p	MIMAT0000828	Fanci	100360594	XM_003753295	1	1	0	1	3
rno-miR-124-3p	MIMAT0000828	Rnf39	171387	NM_134374	1	1	0	1	3
rno-miR-124-3p	MIMAT0000828	LOC1009	100909641	XM_003749755	1	1	0	1	3
rno-miR-124-3p	MIMAT0000828	Fam76a	362618	NM_001108686	1	1	0	1	3
rno-miR-124-3p	MIMAT0000828	Flot1	64665	NM_022701	1	1	0	1	3
rno-miR-124-3p	MIMAT0000828	Entpd3	316077	NM_178106	1	1	0	1	3
rno-miR-124-3p	MIMAT0000828	Gpatch8	685233	XM_003752448	1	1	0	1	3
rno-miR-124-3p	MIMAT0000828	Cep135	305288	XM_223341	1	1	0	1	3
rno-miR-124-3p	MIMAT0000828	Ankrd13b	360575	XM_340854	1	1	0	1	3
rno-miR-124-3p	MIMAT0000828	Hist2h2be	295274	XM_227459	1	1	0	1	3
rno-miR-124-3p	MIMAT0000828	Fitm2	311617	NM_001107799	1	1	0	1	3
rno-miR-124-3p	MIMAT0000828	Rpp25l	298002	NM_001106648	1	1	0	1	3
rno-miR-124-3p	MIMAT0000828	Vipr2	29555	NM_017238	1	1	0	1	3
rno-miR-124-3p	MIMAT0000828	Lzts1	266711	NM_153470	1	1	0	1	3
rno-miR-124-3p	MIMAT0000828	Etv1	362733	NM_001108709	1	1	0	1	3
rno-miR-124-3p	MIMAT0000828	Zfp12	288486	XM_003752555	1	1	0	1	3
rno-miR-124-3p	MIMAT0000828	Sptlc1	361213	NM_001108406	1	1	0	1	3
rno-miR-124-3p	MIMAT0000828	Cdkn2c	54238	NM_131902	1	1	0	1	3
rno-miR-124-3p	MIMAT0000828	Abca1	313210	NM_178095	1	1	0	1	3
rno-miR-124-3p	MIMAT0000828	Lrrc2	301033	NM_001012001	1	1	0	1	3

rno-miR-124-3p	MIMAT0000828	Sowahc	503306	NM_001109364	1	1	0	1	3
rno-miR-124-3p	MIMAT0000828	Atad5	303348	XM_003752347	1	1	0	1	3
rno-miR-124-3p	MIMAT0000828	Adcyap1r	24167	NM_133511	1	1	0	1	3
rno-miR-124-3p	MIMAT0000828	Rab22a	366265	NM_001108966	1	1	0	1	3
rno-miR-124-3p	MIMAT0000828	Dusp3	498003	NM_001173376	1	1	0	1	3
rno-miR-124-3p	MIMAT0000828	Epc1	100362678	XM_003751704	1	1	0	1	3
rno-miR-124-3p	MIMAT0000828	LOC1009	100910779	XM_003751732	1	1	0	1	3
rno-miR-124-3p	MIMAT0000828	Arf2	79119	NM_024150	1	1	0	1	3
rno-miR-124-3p	MIMAT0000828	RGD1563	315652	XM_236231	1	1	0	1	3
rno-miR-124-3p	MIMAT0000828	Ocln	83497	NM_031329	1	1	0	1	3
rno-miR-124-3p	MIMAT0000828	Ung	304577	NM_001013124	1	1	0	1	3
rno-miR-124-3p	MIMAT0000828	LOC6867	686774	XM_003753962	1	1	0	1	3
rno-miR-124-3p	MIMAT0000828	Sbk2	691411	NM_001127539	1	1	0	1	3
rno-miR-124-3p	MIMAT0000828	Pm20d1	498226	NM_001109068	1	1	0	1	3
rno-miR-124-3p	MIMAT0000828	Plekhf2	362484	NM_001108655	1	1	0	1	3
rno-miR-124-3p	MIMAT0000828	Myo5c	315820	XM_003750540	1	1	0	1	3
rno-miR-124-3p	MIMAT0000828	Ppp1r12b	304813	NM_001107178	1	1	0	1	3
rno-miR-124-3p	MIMAT0000828	LOC6919	691920	XM_003753762	1	1	0	1	3
rno-miR-124-3p	MIMAT0000828	Susd2	294335	NM_001106381	1	1	0	1	3
rno-miR-124-3p	MIMAT0000828	Tle4	25565	NM_019141	1	1	0	1	3
rno-miR-124-3p	MIMAT0000828	Stk10	29398	NM_019206	1	1	0	1	3
rno-miR-124-3p	MIMAT0000828	Lrig1	312574	XM_232237	1	1	0	1	3
rno-miR-124-3p	MIMAT0000828	LOC5010	501033	XM_001055454	1	1	0	1	3
rno-miR-124-3p	MIMAT0000828	Frmd4b	252858	XM_232212	1	1	0	1	3
rno-miR-124-3p	MIMAT0000828	Cep164	363055	XM_001064241	1	1	0	1	3
rno-miR-124-3p	MIMAT0000828	Crtc3	365297	XM_001066604	1	1	0	1	3
rno-miR-124-3p	MIMAT0000828	Mmrn2	306288	XM_003752862	1	1	0	1	3
rno-miR-124-3p	MIMAT0000828	Tbc1d1	360937	XM_341215	1	1	0	1	3
rno-miR-124-3p	MIMAT0000828	Srebf2	300095	NM_001033694	1	1	0	1	3
rno-miR-124-3p	MIMAT0000828	RGD1304	313776	NM_001108001	1	1	0	1	3
rno-miR-124-3p	MIMAT0000828	Ammecr1	501539	XM_001057408	1	1	0	1	3
rno-miR-124-3p	MIMAT0000828	Maml1	303101	NM_001106997	1	1	0	1	3
rno-miR-124-3p	MIMAT0000828	Tpk1	680668	NM_001134994	1	1	0	1	3
rno-miR-124-3p	MIMAT0000828	Ovol1	309164	NM_001107572	1	1	0	1	3
rno-miR-124-3p	MIMAT0000828	Pttg1ip	365548	NM_001013238	1	1	0	1	3
rno-miR-124-3p	MIMAT0000828	Gamt	25257	NM_012793	1	1	0	1	3
rno-miR-124-3p	MIMAT0000828	Baalc	140720	NM_144762	1	1	0	1	3
rno-miR-124-3p	MIMAT0000828	Kank2	100361376	XM_003754377	1	1	0	1	3
rno-miR-124-3p	MIMAT0000828	Rps6ka4	361715	NM_001108517	1	1	0	1	3
rno-miR-124-3p	MIMAT0000828	Cldn1	65129	NM_031699	1	1	0	1	3
rno-miR-124-3p	MIMAT0000828	Zbed4	315211	NM_001134800	1	1	0	1	3
rno-miR-124-3p	MIMAT0000828	Gnai2	81664	NM_031035	1	1	0	1	3
rno-miR-124-3p	MIMAT0000828	Fam189b	310640	NM_001107690	1	1	0	1	3
rno-miR-124-3p	MIMAT0000828	Kcnj6	25743	NM_013192	1	1	0	1	3
rno-miR-124-3p	MIMAT0000828	Clmp	286939	NM_173154	1	1	0	1	3
rno-miR-124-3p	MIMAT0000828	LOC1009	100912282	XM_003750418	1	1	0	1	3
rno-miR-124-3p	MIMAT0000828	Ncapg2	362798	XM_001061369	1	1	0	1	3
rno-miR-124-3p	MIMAT0000828	E2f5	116651	XM_001053974	1	1	0	1	3
rno-miR-124-3p	MIMAT0000828	Rreb1	306873	NM_001107348	1	1	0	1	3
rno-miR-124-3p	MIMAT0000828	Arc	54323	NM_019361	1	1	0	1	3
rno-miR-124-3p	MIMAT0000828	Ptprd	313278	XM_233065	1	1	0	1	3
rno-miR-124-3p	MIMAT0000828	Il18r1	301365	NM_001106905	1	1	0	1	3
rno-miR-124-3p	MIMAT0000828	Ubr7	314399	NM_001007705	1	1	0	1	3
rno-miR-124-3p	MIMAT0000828	Dsc2	291760	NM_001033688	1	1	0	1	3
rno-miR-124-3p	MIMAT0000828	Pip4k2a	116723	NM_053926	1	1	0	1	3
rno-miR-124-3p	MIMAT0000828	Zmat3	64394	NM_022548	1	1	0	1	3
rno-miR-124-3p	MIMAT0000828	Ap3d1	314633	NM_001100719	1	1	0	1	3
rno-miR-124-3p	MIMAT0000828	LOC6790	679038	NM_001100998	1	1	0	1	3
rno-miR-124-3p	MIMAT0000828	Ets1	24356	NM_012555	1	1	0	1	3
rno-miR-124-3p	MIMAT0000828	Mrvi1	308899	NM_001105211	1	1	0	1	3
rno-miR-124-3p	MIMAT0000828	RGD1562	292100	NM_001034919	1	1	0	1	3
rno-miR-124-3p	MIMAT0000828	Col6a3	367313	XM_003754548	1	1	0	1	3
rno-miR-124-3p	MIMAT0000828	Hormad2	498400	NM_001017501	1	1	0	1	3
rno-miR-124-3p	MIMAT0000828	Slc2a13	171147	NM_133611	1	1	0	1	3
rno-miR-124-3p	MIMAT0000828	LOC1003	100365752	XM_003753772	1	1	0	1	3
rno-miR-124-3p	MIMAT0000828	Nr6a1	362125	XM_342427	1	1	0	1	3
rno-miR-124-3p	MIMAT0000828	Inhbc	64549	NM_022614	1	1	0	1	3
rno-miR-124-3p	MIMAT0000828	Grb2	81504	NM_030846	1	1	0	1	3
rno-miR-124-3p	MIMAT0000828	Parp9	303905	NM_001103351	1	1	0	1	3

rno-miR-124-3p	MIMAT0000828	Parvg	689069	NM_001130583	1	1	0	1	3
rno-miR-124-3p	MIMAT0000828	Ptpn11	25622	NM_001177593	1	1	0	1	3
rno-miR-124-3p	MIMAT0000828	Kif16b	311478	NM_001107783	1	1	0	1	3
rno-miR-124-3p	MIMAT0000828	Mkrn2	297525	NM_001008314	1	1	0	1	3
rno-miR-124-3p	MIMAT0000828	Padi2	29511	NM_017226	1	1	0	1	3
rno-miR-124-3p	MIMAT0000828	Kcnip4	259243	NM_181365	1	1	0	1	3
rno-miR-124-3p	MIMAT0000828	LOC1009	100911874	XM_003750695	1	1	0	1	3
rno-miR-124-3p	MIMAT0000828	Cldnd1	288182	NM_001006955	1	1	0	1	3
rno-miR-124-3p	MIMAT0000828	Agpat5	306582	NM_001134744	1	1	0	1	3
rno-miR-124-3p	MIMAT0000828	Ints6	361057	XM_003751499	1	1	0	1	3
rno-miR-124-3p	MIMAT0000828	Gja5	50563	NM_019280	1	1	0	1	3
rno-miR-124-3p	MIMAT0000828	Fut4	60670	NM_022219	1	1	0	1	3
rno-miR-124-3p	MIMAT0000828	Sorl1	300652	NM_053519	1	1	0	1	3
rno-miR-124-3p	MIMAT0000828	RGD1559	502418	NM_001109337	1	1	0	1	3
rno-miR-124-3p	MIMAT0000828	Sdc4	24771	NM_012649	1	1	0	1	3
rno-miR-124-3p	MIMAT0000828	Hormad1	365868	NM_001108949	1	1	0	1	3
rno-miR-124-3p	MIMAT0000828	Acadvl	25363	NM_012891	1	1	0	1	3
rno-miR-124-3p	MIMAT0000828	Nudt16l1	497867	NM_001100782	1	1	0	1	3
rno-miR-124-3p	MIMAT0000828	Ryr3	170546	XM_001080527	1	1	0	1	3
rno-miR-124-3p	MIMAT0000828	Lemd2	361807	NM_001039032	1	1	0	1	3
rno-miR-124-3p	MIMAT0000828	LOC1009	100910536	XM_003750382	1	1	0	1	3
rno-miR-124-3p	MIMAT0000828	Jam3	315509	NM_001004269	1	1	0	1	3
rno-miR-124-3p	MIMAT0000828	Lama1	316758	NM_001108237	1	1	0	1	3
rno-miR-124-3p	MIMAT0000828	Sorbs1	686098	XM_003749112	1	1	0	1	3
rno-miR-124-3p	MIMAT0000828	Wdhd1	305827	NM_001107255	1	1	0	1	3
rno-miR-124-3p	MIMAT0000828	Fbxo3	690634	NM_001109606	1	1	0	1	3
rno-miR-124-3p	MIMAT0000828	Srxn1	296271	NM_001047858	1	1	0	1	3
rno-miR-124-3p	MIMAT0000828	H6pd	298655	NM_001106698	1	1	0	1	3
rno-miR-124-3p	MIMAT0000828	Dact1	500666	XM_002726752	1	1	0	1	3
rno-miR-124-3p	MIMAT0000828	LOC1009	100912585	XM_003752335	1	1	0	1	3
rno-miR-124-3p	MIMAT0000828	Shisa7	691429	NM_001145175	1	1	0	1	3
rno-miR-124-3p	MIMAT0000828	Nid1	25494	XM_001054079	1	1	0	1	3
rno-miR-124-3p	MIMAT0000828	Rab11fip5	312502	XM_003753914	1	1	0	1	3
rno-miR-124-3p	MIMAT0000828	Arhgap39	500901	NM_173122	1	1	0	1	3
rno-miR-124-3p	MIMAT0000828	LOC1009	100911261	XM_003751445	1	1	0	1	3
rno-miR-124-3p	MIMAT0000828	Itga8	364786	NM_001173972	1	1	0	1	3
rno-miR-124-3p	MIMAT0000828	Vcan	114122	NM_001170559	1	1	0	1	3
rno-miR-124-3p	MIMAT0000828	Fbxo38	307390	XM_003753052	1	1	0	1	3
rno-miR-124-3p	MIMAT0000828	Klf6	58954	NM_031642	1	1	0	1	3
rno-miR-124-3p	MIMAT0000828	Abca3	302973	XM_003752300	1	1	0	1	3
rno-miR-124-3p	MIMAT0000828	Nav3	314814	NM_001191782	1	1	0	1	3
rno-miR-124-3p	MIMAT0000828	Nat8l	289727	NM_001191681	1	1	0	1	3
rno-miR-124-3p	MIMAT0000828	Thumpd1	309041	NM_001009688	1	1	0	1	3
rno-miR-124-3p	MIMAT0000828	Pcdha2	393086	NM_199504	1	1	0	1	3
rno-miR-124-3p	MIMAT0000828	RGD1561	498580	NM_001134605	1	1	0	1	3
rno-miR-124-3p	MIMAT0000828	Iqgap2	100360623	XM_002729002	1	1	0	1	3
rno-miR-124-3p	MIMAT0000828	Micu2	171433	NM_134396	1	1	0	1	3
rno-miR-124-3p	MIMAT0000828	Dusp15	362238	NM_001108598	1	1	0	1	3
rno-miR-124-3p	MIMAT0000828	Tfrc	64678	NM_022712	1	1	0	1	3
rno-miR-124-3p	MIMAT0000828	Eny2	685258	NM_001130580	1	1	0	1	3
rno-miR-124-3p	MIMAT0000828	Nxn	360577	NM_001108285	1	1	0	1	3
rno-miR-124-3p	MIMAT0000828	Nrip2	689619	XM_001071392	1	1	0	1	3
rno-miR-124-3p	MIMAT0000828	Acp6	295305	NM_001031645	1	1	0	1	3
rno-miR-124-3p	MIMAT0000828	Hmgcr	25675	NM_013134	1	1	0	1	3
rno-miR-124-3p	MIMAT0000828	Tomm34	311621	NM_001044244	1	1	0	1	3
rno-miR-124-3p	MIMAT0000828	RGD1562	498764	XM_001056659	1	1	0	1	3
rno-miR-124-3p	MIMAT0000828	Tex10	298065	NM_001106653	1	1	0	1	3
rno-miR-124-3p	MIMAT0000828	Etv1	362733	NM_001163156	1	1	0	1	3
rno-miR-124-3p	MIMAT0000828	Zfp12	288486	XM_221917	1	1	0	1	3
rno-miR-124-3p	MIMAT0000828	Zfp367	306695	NM_001012051	1	1	0	1	3
rno-miR-124-3p	MIMAT0000828	Gata4	54254	NM_144730	1	1	0	1	3
rno-miR-124-3p	MIMAT0000828	Atad5	303348	XM_220750	1	1	0	1	3
rno-miR-124-3p	MIMAT0000828	Vhl	24874	NM_052801	1	1	0	1	3
rno-miR-124-3p	MIMAT0000828	Ptk6	366275	NM_001108968	1	1	0	1	3
rno-miR-124-3p	MIMAT0000828	Anapc11	498030	NM_001126082	1	1	0	1	3
rno-miR-124-3p	MIMAT0000828	Epc1	100362678	XM_003752989	1	1	0	1	3
rno-miR-124-3p	MIMAT0000828	LOC1009	100910779	XM_003751733	1	1	0	1	3
rno-miR-124-3p	MIMAT0000828	Npat	315666	NM_001108147	1	1	0	1	3
rno-miR-124-3p	MIMAT0000828	Ppp1r13l	686781	XM_001075705	1	1	0	1	3

rno-miR-124-3p	MIMAT0000828	Anks1a	309639	NM_001107613	1	1	0	1	3
rno-miR-124-3p	MIMAT0000828	Ap1m2	367038	NM_001108996	1	1	0	1	3
rno-miR-124-3p	MIMAT0000828	Ormdl1	100188936	NM_001134704	1	1	0	1	3
rno-miR-124-3p	MIMAT0000828	Trak2	171086	NM_133560	1	1	0	1	3
rno-miR-124-3p	MIMAT0000828	Cd7	303747	NM_001107074	1	1	0	1	3
rno-miR-124-3p	MIMAT0000828	Myo5c	315820	XM_003754468	1	1	0	1	3
rno-miR-124-3p	MIMAT0000828	Tor1aip2	304881	NM_001165896	1	1	0	1	3
rno-miR-124-3p	MIMAT0000828	Ccdc137	688298	NM_001143896	1	1	0	1	3
rno-miR-124-3p	MIMAT0000828	LOC6919	691952	NM_001109652	1	1	0	1	3
rno-miR-124-3p	MIMAT0000828	RGD1564	499758	NM_001126295	1	1	0	1	3
rno-miR-124-3p	MIMAT0000828	LOC5010	501033	XM_001066296	1	1	0	1	3
rno-miR-124-3p	MIMAT0000828	Cep164	363055	XM_343383	1	1	0	1	3
rno-miR-124-3p	MIMAT0000828	Mapk7	114509	NM_001191547	1	1	0	1	3
rno-miR-124-3p	MIMAT0000828	P2rx7	29665	NM_019256	1	1	0	1	3
rno-miR-124-3p	MIMAT0000828	Map3k15	501558	XM_001054480	1	1	0	1	3
rno-miR-124-3p	MIMAT0000828	Aff4	303132	NM_001107001	1	1	0	1	3
rno-miR-124-3p	MIMAT0000828	Cldn10	290485	NM_001106058	1	1	0	1	3
rno-miR-124-3p	MIMAT0000828	Myh11	24582	NM_001170600	1	1	0	1	3
rno-miR-124-3p	MIMAT0000828	Rtdr1	365552	NM_001127557	1	1	0	1	3
rno-miR-124-3p	MIMAT0000828	Htatip2	292935	NM_001106263	1	1	0	1	3
rno-miR-124-3p	MIMAT0000828	Nnt	310378	NM_001013157	1	1	0	1	3
rno-miR-124-3p	MIMAT0000828	Caskin1	140722	NM_080690	1	1	0	1	3
rno-miR-124-3p	MIMAT0000828	RGD1308	361719	NM_001134575	1	1	0	1	3
rno-miR-124-3p	MIMAT0000828	Elmo2	362271	NM_001134955	1	1	0	1	3
rno-miR-124-3p	MIMAT0000828	Mlc1	315215	NM_001108105	1	1	0	1	3
rno-miR-124-3p	MIMAT0000828	Itpr2	81678	NM_031046	1	1	0	1	3
rno-miR-124-3p	MIMAT0000828	Znf740	685834	XM_001065442	1	1	0	1	3
rno-miR-124-3p	MIMAT0000828	Cgn	310655	XM_001059265	1	1	0	1	3
rno-miR-124-3p	MIMAT0000828	Frzb	295691	NM_001100527	1	1	0	1	3
rno-miR-124-3p	MIMAT0000828	Il2rb	25746	NM_013195	1	1	0	1	3
rno-miR-124-3p	MIMAT0000828	Ldlrap1	500564	NM_001109271	1	1	0	1	3
rno-miR-124-3p	MIMAT0000828	Ugt2b1	286954	NM_173295	1	1	0	1	3
rno-miR-124-3p	MIMAT0000828	Ncapg2	362798	XM_343124	1	1	0	1	3
rno-miR-124-3p	MIMAT0000828	Vegp2	94106	NM_053574	1	1	0	1	3
rno-miR-124-3p	MIMAT0000828	E2f5	116651	XM_574892	1	1	0	1	3
rno-miR-124-3p	MIMAT0000828	Ripk1	306886	NM_001107350	1	1	0	1	3
rno-miR-124-3p	MIMAT0000828	Stk39	54348	NM_019362	1	1	0	1	3
rno-miR-124-3p	MIMAT0000828	RGD1562	291773	XM_001073245	1	1	0	1	3
rno-miR-124-3p	MIMAT0000828	Dock5	305987	XM_003751494	1	1	0	1	3
rno-miR-124-3p	MIMAT0000828	Pcdha4	116741	NM_053933	1	1	0	1	3
rno-miR-124-3p	MIMAT0000828	Emc8	361425	NM_001012165	1	1	0	1	3
rno-miR-124-3p	MIMAT0000828	Arel1	299197	NM_001106744	1	1	0	1	3
rno-miR-124-3p	MIMAT0000828	Tmem8b	313490	XM_001070176	1	1	0	1	3
rno-miR-124-3p	MIMAT0000828	St3gal2	64442	NM_031695	1	1	0	1	3
rno-miR-124-3p	MIMAT0000828	Zfr2	314639	XM_001070148	1	1	0	1	3
rno-miR-124-3p	MIMAT0000828	Garem	679154	XM_002725336	1	1	0	1	3
rno-miR-124-3p	MIMAT0000828	Tmem63a	289318	NM_001134496	1	1	0	1	3
rno-miR-124-3p	MIMAT0000828	Fancc	24361	NM_012557	1	1	0	1	3
rno-miR-124-3p	MIMAT0000828	Habp2	292126	NM_001001505	1	1	0	1	3
rno-miR-124-3p	MIMAT0000828	RGD1310	309809	XM_001063192	1	1	0	1	3
rno-miR-124-3p	MIMAT0000828	LOC1003	100360087	XM_002726637	1	1	0	1	3
rno-miR-124-3p	MIMAT0000828	Cdk7	171150	XM_001071127	1	1	0	1	3
rno-miR-124-3p	MIMAT0000828	LOC1003	100366044	XM_002727475	1	1	0	1	3
rno-miR-124-3p	MIMAT0000828	Lnp	362151	NM_001077429	1	1	0	1	3
rno-miR-124-3p	MIMAT0000828	Akap6	64553	NM_022618	1	1	0	1	3
rno-miR-124-3p	MIMAT0000828	Ildr1	303914	NM_001127536	1	1	0	1	3
rno-miR-124-3p	MIMAT0000828	Msl2	315959	XM_001071576	1	1	0	1	3
rno-miR-124-3p	MIMAT0000828	Klhdc8a	305096	NM_001100683	1	1	0	1	3
rno-miR-124-3p	MIMAT0000828	Ptpn11	25622	NM_013088	1	1	0	1	3
rno-miR-124-3p	MIMAT0000828	Mrps35	297727	NM_001106628	1	1	0	1	3
rno-miR-124-3p	MIMAT0000828	Snx27	260323	NM_001110151	1	1	0	1	3
rno-miR-124-3p	MIMAT0000828	Crybg3	288204	XM_001057435	1	1	0	1	3
rno-miR-124-3p	MIMAT0000828	Atp1b4	84396	NM_053381	1	1	0	1	3
rno-miR-124-3p	MIMAT0000828	Usp38	307764	XM_003751862	1	1	0	1	3
rno-miR-124-3p	MIMAT0000828	Actn4	63836	NM_031675	1	1	0	1	3
rno-miR-124-3p	MIMAT0000828	Cxcl12	24772	NM_001033883	1	1	0	1	3
rno-miR-124-3p	MIMAT0000828	Hipk1	365895	NM_001100986	1	1	0	1	3
rno-miR-124-3p	MIMAT0000828	Adarb1	25367	NM_001111055	1	1	0	1	3
rno-miR-124-3p	MIMAT0000828	Ryr3	170546	XM_342491	1	1	0	1	3

rno-miR-124-3p	MIMAT0000828	Stk38	361813	NM_001015025	1	1	0	1	3
rno-miR-124-3p	MIMAT0000828	Zc3h14	192359	NM_001033951	1	1	0	1	3
rno-miR-124-3p	MIMAT0000828	LOC1009	100910536	XM_003750383	1	1	0	1	3
rno-miR-124-3p	MIMAT0000828	Sorbs1	686098	XM_003749113	1	1	0	1	3
rno-miR-124-3p	MIMAT0000828	Plagl2	296281	NM_001106528	1	1	0	1	3
rno-miR-124-3p	MIMAT0000828	Ctxn1	29145	NM_001109935	1	1	0	1	3
rno-miR-124-3p	MIMAT0000828	Raver1	298705	NM_001013939	1	1	0	1	3
rno-miR-124-3p	MIMAT0000828	Dact1	500666	XM_002729635	1	1	0	1	3
rno-miR-124-3p	MIMAT0000828	Rpl26	287417	NM_001105788	1	1	0	1	3
rno-miR-124-3p	MIMAT0000828	Mybpc1	362867	NM_001100758	1	1	0	1	3
rno-miR-124-3p	MIMAT0000828	Ppp1r13l	686781	XM_002728700	1	1	0	1	3
rno-miR-124-3p	MIMAT0000828	Nid1	25494	XM_213954	1	1	0	1	3
rno-miR-124-3p	MIMAT0000828	Asb6	296627	NM_001011963	1	1	0	1	3
rno-miR-124-3p	MIMAT0000828	Tnfrsf13c	500910	XM_001077542	1	1	0	1	3
rno-miR-124-3p	MIMAT0000828	LOC1009	100911319	XM_003750261	1	1	0	1	3
rno-miR-124-3p	MIMAT0000828	Sarm1	287545	NM_001105817	1	1	0	1	3
rno-miR-124-3p	MIMAT0000828	Dennd6b	362983	NM_001108749	1	1	0	1	3
rno-miR-124-3p	MIMAT0000828	Apba3	83611	NM_031781	1	1	0	1	3
rno-miR-124-3p	MIMAT0000828	Vcan	114122	NM_001170560	1	1	0	1	3
rno-miR-124-3p	MIMAT0000828	Arhgef37	307398	NM_001100974	1	1	0	1	3
rno-miR-124-3p	MIMAT0000828	Btg2	29619	NM_017259	1	1	0	1	3
rno-miR-124-3p	MIMAT0000828	Aldh1l2	299699	NM_001191778	1	1	0	1	3
rno-miR-124-3p	MIMAT0000828	Mfap2	313662	NM_001107989	1	1	0	1	3
rno-miR-124-3p	MIMAT0000828	Abca3	302973	XM_220219	1	1	0	1	3
rno-miR-124-3p	MIMAT0000828	E2f7	314818	NM_001108092	1	1	0	1	3
rno-miR-124-3p	MIMAT0000828	RGD1307	309053	NM_001134546	1	1	0	1	3
rno-miR-124-3p	MIMAT0000828	Ltbp4	292734	NM_001170336	1	1	0	1	3
rno-miR-124-3p	MIMAT0000828	Pcdha5	393087	NM_199505	1	1	0	1	3
rno-miR-124-3p	MIMAT0000828	Iqgap2	100360623	XM_003753523	1	1	0	1	3
rno-miR-124-3p	MIMAT0000828	Phldb1	171434	NM_001191578	1	1	0	1	3
rno-miR-124-3p	MIMAT0000828	Dusp15	362238	NM_001244784	1	1	0	1	3
rno-miR-124-3p	MIMAT0000828	Mvp	64681	NM_022715	1	1	0	1	3
rno-miR-124-3p	MIMAT0000828	Cldn8	304124	NM_001037774	1	1	0	1	3
rno-miR-124-3p	MIMAT0000828	Polr3g	685465	NM_001109468	1	1	0	1	3
rno-miR-124-3p	MIMAT0000828	Nrip2	689619	XM_003753948	1	1	0	1	3
rno-miR-124-3p	MIMAT0000828	Rap1a	295347	NM_001005765	1	1	0	1	3
rno-miR-124-3p	MIMAT0000828	Stk4	311622	NM_001107800	1	1	0	1	3
rno-miR-124-3p	MIMAT0000828	RGD1562	498764	XM_574050	1	1	0	1	3
rno-miR-124-3p	MIMAT0000828	Trmt10b	298081	NM_001013090	1	1	0	1	3
rno-miR-124-3p	MIMAT0000828	Fbxo36	363268	NM_001108804	1	1	0	1	3
rno-miR-124-3p	MIMAT0000828	Zfp189	313219	NM_001107930	1	1	0	1	3
rno-miR-124-3p	MIMAT0000828	Aldh9a1	64040	NM_022273	1	1	0	1	3
rno-miR-124-3p	MIMAT0000828	Cbll1	314028	NM_001108018	1	1	0	1	3
rno-miR-124-3p	MIMAT0000828	Nudt13	682978	NM_001127636	1	1	0	1	3
rno-miR-124-3p	MIMAT0000828	Zfp110	308362	NM_001024775	1	1	0	1	3
rno-miR-124-3p	MIMAT0000828	E2f3	291105	NM_001137626	1	1	0	1	3
rno-miR-124-3p	MIMAT0000828	Slc5a9	366441	NM_001108974	1	1	0	1	3
rno-miR-124-3p	MIMAT0000828	Eif1ad	293673	NM_001008305	1	1	0	1	3
rno-miR-124-3p	MIMAT0000828	LOC1003	100363177	XM_002729015	1	1	0	1	3
rno-miR-124-3p	MIMAT0000828	Zbed3	361881	NM_001025729	1	1	0	1	3
rno-miR-124-3p	MIMAT0000828	LOC1009	100910868	XM_003749788	1	1	0	1	3
rno-miR-124-3p	MIMAT0000828	RGD1562	291773	XM_002728524	1	1	0	1	3
rno-miR-124-3p	MIMAT0000828	Xrcc6	25019	NM_139080	1	1	0	1	3
rno-miR-124-3p	MIMAT0000828	Dock6	367039	NM_001108997	1	1	0	1	3
rno-miR-124-3p	MIMAT0000828	Fam163a	498257	NM_001109072	1	1	0	1	3
rno-miR-124-3p	MIMAT0000828	Pnkd	100188944	NM_001134750	1	1	0	1	3
rno-miR-124-3p	MIMAT0000828	LOC1003	100363915	XM_003752624	1	1	0	1	3
rno-miR-124-3p	MIMAT0000828	RGD1309	362020	NM_001134578	1	1	0	1	3
rno-miR-124-3p	MIMAT0000828	Fbxo10	362511	XM_001071167	1	1	0	1	3
rno-miR-124-3p	MIMAT0000828	Luzp1	79428	NM_030830	1	1	0	1	3
rno-miR-124-3p	MIMAT0000828	Tor1aip2	304881	NM_001165897	1	1	0	1	3
rno-miR-124-3p	MIMAT0000828	Flad1	751787	NM_001110138	1	1	0	1	3
rno-miR-124-3p	MIMAT0000828	Asf1a	294408	NM_001106389	1	1	0	1	3
rno-miR-124-3p	MIMAT0000828	Kdr	25589	NM_013062	1	1	0	1	3
rno-miR-124-3p	MIMAT0000828	Por	29441	NM_031576	1	1	0	1	3
rno-miR-124-3p	MIMAT0000828	Cmtm7	501065	NM_001109300	1	1	0	1	3
rno-miR-124-3p	MIMAT0000828	Tmem106	287722	NM_001024967	1	1	0	1	3
rno-miR-124-3p	MIMAT0000828	Jakmip3	365380	NM_001163277	1	1	0	1	3
rno-miR-124-3p	MIMAT0000828	Psd2	307500	NM_001107395	1	1	0	1	3

rno-miR-124-3p	MIMAT0000828	Slc8a1	29715	NM_019268	1	1	0	1	3
rno-miR-124-3p	MIMAT0000828	Trabd	300142	NM_001106788	1	1	0	1	3
rno-miR-124-3p	MIMAT0000828	Map3k15	501558	XM_576963	1	1	0	1	3
rno-miR-124-3p	MIMAT0000828	LOC3031	303140	XM_001073573	1	1	0	1	3
rno-miR-124-3p	MIMAT0000828	Gnal	24611	XM_001060758	1	1	0	1	3
rno-miR-124-3p	MIMAT0000828	Map3k11	309168	NM_001013150	1	1	0	1	3
rno-miR-124-3p	MIMAT0000828	Tysnd1	365571	NM_001108932	1	1	0	1	3
rno-miR-124-3p	MIMAT0000828	RGD1309	292947	XM_003753132	1	1	0	1	3
rno-miR-124-3p	MIMAT0000828	Ccrn4l	310395	NM_138526	1	1	0	1	3
rno-miR-124-3p	MIMAT0000828	Il22ra2	444986	NM_001003404	1	1	0	1	3
rno-miR-124-3p	MIMAT0000828	Cyb561a3	361729	NM_001014164	1	1	0	1	3
rno-miR-124-3p	MIMAT0000828	Hip1	192154	NM_001100475	1	1	0	1	3
rno-miR-124-3p	MIMAT0000828	LOC1009	100910054	XM_003754839	1	1	0	1	3
rno-miR-124-3p	MIMAT0000828	Znf740	685834	XM_003754358	1	1	0	1	3
rno-miR-124-3p	MIMAT0000828	Cgn	310655	XM_227472	1	1	0	1	3
rno-miR-124-3p	MIMAT0000828	Gapvd1	311880	XM_001078902	1	1	0	1	3
rno-miR-124-3p	MIMAT0000828	Dnali1	298524	NM_001031647	1	1	0	1	3
rno-miR-124-3p	MIMAT0000828	Stpg1	500566	NM_001025772	1	1	0	1	3
rno-miR-124-3p	MIMAT0000828	LOC1009	100912409	XM_003748999	1	1	0	1	3
rno-miR-124-3p	MIMAT0000828	Cwf19l2	362804	NM_001135003	1	1	0	1	3
rno-miR-124-3p	MIMAT0000828	Tom1	361370	NM_001008365	1	1	0	1	3
rno-miR-124-3p	MIMAT0000828	Prrx2	113931	NM_001105739	1	1	0	1	3
rno-miR-124-3p	MIMAT0000828	Dock5	305987	XM_003752822	1	1	0	1	3
rno-miR-124-3p	MIMAT0000828	Pcdha13	116742	NM_053934	1	1	0	1	3
rno-miR-124-3p	MIMAT0000828	Fam210a	307343	NM_001007688	1	1	0	1	3
rno-miR-124-3p	MIMAT0000828	Nek9	299204	NM_001106747	1	1	0	1	3
rno-miR-124-3p	MIMAT0000828	Tmem8b	313490	XM_233376	1	1	0	1	3
rno-miR-124-3p	MIMAT0000828	Zfr2	314639	XM_234923	1	1	0	1	3
rno-miR-124-3p	MIMAT0000828	Tsc22d4	684980	NM_001044284	1	1	0	1	3
rno-miR-124-3p	MIMAT0000828	Gfap	24387	NM_017009	1	1	0	1	3
rno-miR-124-3p	MIMAT0000828	Tmem219	308986	XM_001080074	1	1	0	1	3
rno-miR-124-3p	MIMAT0000828	Tdrd1	292129	NM_001106202	1	1	0	1	3
rno-miR-124-3p	MIMAT0000828	Ntrk2	25054	NM_001163168	1	1	0	1	3
rno-miR-124-3p	MIMAT0000828	RGD1310	309809	XM_228270	1	1	0	1	3
rno-miR-124-3p	MIMAT0000828	LOC1003	100360087	XM_002729553	1	1	0	1	3
rno-miR-124-3p	MIMAT0000828	Cdk7	171150	XM_215467	1	1	0	1	3
rno-miR-124-3p	MIMAT0000828	LOC1003	100366121	XM_002726871	1	1	0	1	3
rno-miR-124-3p	MIMAT0000828	Prr5l	362171	NM_001080150	1	1	0	1	3
rno-miR-124-3p	MIMAT0000828	Msl2	315959	XM_236567	1	1	0	1	3
rno-miR-124-3p	MIMAT0000828	Gpr133	689257	XM_001070157	1	1	0	1	3
rno-miR-124-3p	MIMAT0000828	Armc1	294948	NM_001106425	1	1	0	1	3
rno-miR-124-3p	MIMAT0000828	Zbtb8os	297885	XM_003750036	1	1	0	1	3
rno-miR-124-3p	MIMAT0000828	Snx27	260323	NM_152847	1	1	0	1	3
rno-miR-124-3p	MIMAT0000828	Crybg3	288204	XM_213639	1	1	0	1	3
rno-miR-124-3p	MIMAT0000828	Usp38	307764	XM_003753096	1	1	0	1	3
rno-miR-124-3p	MIMAT0000828	Rora	300807	NM_001106834	1	1	0	1	3
rno-miR-124-3p	MIMAT0000828	Hs1bp3	313950	XM_001071788	1	1	0	1	3
rno-miR-124-3p	MIMAT0000828	Ddx26b	681665	XM_002727691	1	1	0	1	3
rno-miR-124-3p	MIMAT0000828	RGD1564	290805	XM_001059940	1	1	0	1	3
rno-miR-124-3p	MIMAT0000828	Slc4a1	24779	NM_012651	1	1	0	1	3
rno-miR-124-3p	MIMAT0000828	Arrdc1	366001	NM_001100770	1	1	0	1	3
rno-miR-124-3p	MIMAT0000828	Ccdc69	497906	NM_001109031	1	1	0	1	3
rno-miR-124-3p	MIMAT0000828	Hadha	170670	NM_130826	1	1	0	1	3
rno-miR-124-3p	MIMAT0000828	Cyhr1	100362155	XM_002729828	1	1	0	1	3
rno-miR-124-3p	MIMAT0000828	Srsf3	361814	NM_001047907	1	1	0	1	3
rno-miR-124-3p	MIMAT0000828	LOC1009	100910623	XM_003750782	1	1	0	1	3
rno-miR-124-3p	MIMAT0000828	Slc13a2	65202	NM_031746	1	1	0	1	3
rno-miR-124-3p	MIMAT0000828	Ttc28	304558	XM_001080633	1	1	0	1	3
rno-miR-124-3p	MIMAT0000828	Ephb4	686310	XM_001069453	1	1	0	1	3
rno-miR-124-3p	MIMAT0000828	Zfp219	305848	NM_001007681	1	1	0	1	3
rno-miR-124-3p	MIMAT0000828	RGD1566	499244	XM_001077872	1	1	0	1	3
rno-miR-124-3p	MIMAT0000828	Zfp36l2	298765	NM_001036626	1	1	0	1	3
rno-miR-124-3p	MIMAT0000828	Dact1	500666	XM_576044	1	1	0	1	3
rno-miR-124-3p	MIMAT0000828	Elk3	362871	NM_001108743	1	1	0	1	3
rno-miR-124-3p	MIMAT0000828	Rnase9	364301	NM_001008561	1	1	0	1	3
rno-miR-124-3p	MIMAT0000828	Slc6a2	83511	NM_031343	1	1	0	1	3
rno-miR-124-3p	MIMAT0000828	Notch1	25496	NM_001105721	1	1	0	1	3
rno-miR-124-3p	MIMAT0000828	Phf19	296653	NM_001106570	1	1	0	1	3

rno-miR-124-3p	MIMAT0000828	Tyms	29261	NM_019179	1	1	0	1	3
rno-miR-124-3p	MIMAT0000828	Mcm2	312538	NM_001107873	1	1	0	1	3
rno-miR-124-3p	MIMAT0000828	Ccno	499528	NM_001109175	1	1	0	1	3
rno-miR-124-3p	MIMAT0000828	Tnfrsf13c	500910	XM_576316	1	1	0	1	3
rno-miR-124-3p	MIMAT0000828	LOC1009	100911356	XM_003751804	1	1	0	1	3
rno-miR-124-3p	MIMAT0000828	Cd82	83628	NM_031797	1	1	0	1	3
rno-miR-124-3p	MIMAT0000828	Vcan	114122	NM_053663	1	1	0	1	3
rno-miR-124-3p	MIMAT0000828	Ralgds	29622	NM_019250	1	1	0	1	3
rno-miR-124-3p	MIMAT0000828	Rnf151	302977	NM_001106987	1	1	0	1	3
rno-miR-124-3p	MIMAT0000828	Osbpl8	314824	XM_003750319	1	1	0	1	3
rno-miR-124-3p	MIMAT0000828	Upp1	289801	NM_001030025	1	1	0	1	3
rno-miR-124-3p	MIMAT0000828	Fam53b	309060	NM_001107556	1	1	0	1	3
rno-miR-124-3p	MIMAT0000828	Shkbp1	292735	XM_003753225	1	1	0	1	3
rno-miR-124-3p	MIMAT0000828	Pcdha6	393088	NM_199506	1	1	0	1	3
rno-miR-124-3p	MIMAT0000828	Atp6v1b2	117596	NM_057213	1	1	0	1	3
rno-miR-124-3p	MIMAT0000828	Abtb2	171440	NM_134403	1	1	0	1	3
rno-miR-124-3p	MIMAT0000828	Snta1	362242	NM_001100901	1	1	0	1	3
rno-miR-124-3p	MIMAT0000828	Anxa9	689830	XM_001072193	1	1	0	1	3
rno-miR-124-3p	MIMAT0000828	Usp53	295425	NM_001106468	1	1	0	1	3
rno-miR-124-3p	MIMAT0000828	Col10a1	25681	XM_001053056	1	1	0	1	3
rno-miR-124-3p	MIMAT0000828	Slc9a8	311651	NM_001025281	1	1	0	1	3
rno-miR-124-3p	MIMAT0000828	Ccdc3	498795	XM_001071191	1	1	0	1	3
rno-miR-124-3p	MIMAT0000828	Tgfbr1	29591	NM_012775	1	1	0	1	3
rno-miR-124-3p	MIMAT0000828	Wdr54	500226	NM_001109245	1	1	0	1	3
rno-miR-124-3p	MIMAT0000828	Rcan1	266766	NM_153724	1	1	0	1	3
rno-miR-124-3p	MIMAT0000828	Eif3b	288516	NM_001031640	1	1	0	1	3
rno-miR-124-3p	MIMAT0000828	Slc25a39	360636	NM_001024792	1	1	0	1	3
rno-miR-124-3p	MIMAT0000828	Snap29	116500	NM_053810	1	1	0	1	3
rno-miR-124-3p	MIMAT0000828	RGD1308	361253	NM_001014146	1	1	0	1	3
rno-miR-124-3p	MIMAT0000828	Prpf39	314171	XM_003750171	1	1	0	1	3
rno-miR-124-3p	MIMAT0000828	Dcdc2	291130	NM_001106110	1	1	0	1	3
rno-miR-124-3p	MIMAT0000828	Hectd2	309514	XM_003749094	1	1	0	1	3
rno-miR-124-3p	MIMAT0000828	Epha2	366492	NM_001108977	1	1	0	1	3
rno-miR-124-3p	MIMAT0000828	Lgals12	293710	NM_001106333	1	1	0	1	3
rno-miR-124-3p	MIMAT0000828	Nap1l3	170914	NM_133402	1	1	0	1	3
rno-miR-124-3p	MIMAT0000828	LOC1003	100363177	XM_003753538	1	1	0	1	3
rno-miR-124-3p	MIMAT0000828	Tpcn1	246215	NM_139332	1	1	0	1	3
rno-miR-124-3p	MIMAT0000828	LOC1009	100910881	XM_003751735	1	1	0	1	3
rno-miR-124-3p	MIMAT0000828	Slc25a26	362403	NM_001108638	1	1	0	1	3
rno-miR-124-3p	MIMAT0000828	Kif18b	303575	NM_001039019	1	1	0	1	3
rno-miR-124-3p	MIMAT0000828	Arid3b	367092	NM_001109001	1	1	0	1	3
rno-miR-124-3p	MIMAT0000828	Pnkd	100188944	NM_001134751	1	1	0	1	3
rno-miR-124-3p	MIMAT0000828	LOC1003	100364138	XM_002726633	1	1	0	1	3
rno-miR-124-3p	MIMAT0000828	Agl	362029	NM_001108564	1	1	0	1	3
rno-miR-124-3p	MIMAT0000828	Fbxo10	362511	XM_342829	1	1	0	1	3
rno-miR-124-3p	MIMAT0000828	Bcl6	303836	NM_001107084	1	1	0	1	3
rno-miR-124-3p	MIMAT0000828	LOC1001	100125362	NM_001103354	1	1	0	1	3
rno-miR-124-3p	MIMAT0000828	Emc3	312640	NM_001008355	1	1	0	1	3
rno-miR-124-3p	MIMAT0000828	Pdcd6ip	501083	NM_001029910	1	1	0	1	3
rno-miR-124-3p	MIMAT0000828	Clcf1	365395	NM_207615	1	1	0	1	3
rno-miR-124-3p	MIMAT0000828	Scd	83792	NM_031841	1	1	0	1	3
rno-miR-124-3p	MIMAT0000828	Pnpla2	361676	NM_001108509	1	1	0	1	3
rno-miR-124-3p	MIMAT0000828	LOC3031	303140	XM_220427	1	1	0	1	3
rno-miR-124-3p	MIMAT0000828	S100a4	24615	NM_012618	1	1	0	1	3
rno-miR-124-3p	MIMAT0000828	Dnajb6	362293	NM_001013209	1	1	0	1	3
rno-miR-124-3p	MIMAT0000828	Dhodh	65156	NM_001008553	1	1	0	1	3
rno-miR-124-3p	MIMAT0000828	Lmf2	315218	NM_001079939	1	1	0	1	3
rno-miR-124-3p	MIMAT0000828	Mvk	81727	NM_031063	1	1	0	1	3
rno-miR-124-3p	MIMAT0000828	Zscan21	304342	NM_001012021	1	1	0	1	3
rno-miR-124-3p	MIMAT0000828	Ccdc88a	305605	XM_001065246	1	1	0	1	3
rno-miR-124-3p	MIMAT0000828	Adarb1	25367	NM_001111056	1	1	0	1	3
rno-miR-124-3p	MIMAT0000828	Rfx5	310659	NM_001107694	1	1	0	1	3
rno-miR-124-3p	MIMAT0000828	Dnm2	25751	NM_013199	1	1	0	1	3
rno-miR-124-3p	MIMAT0000828	Gapvd1	311880	XM_231161	1	1	0	1	3
rno-miR-124-3p	MIMAT0000828	Tmco4	500573	NM_001034949	1	1	0	1	3
rno-miR-124-3p	MIMAT0000828	LOC1009	100912429	XM_003751448	1	1	0	1	3
rno-miR-124-3p	MIMAT0000828	Havcr2	363578	NM_001100762	1	1	0	1	3
rno-miR-124-3p	MIMAT0000828	Narf	360681	NM_001039207	1	1	0	1	3
rno-miR-124-3p	MIMAT0000828	Rbm15	684233	XM_001068152	1	1	0	1	3

rno-miR-124-3p	MIMAT0000828	Abhd4	364380	NM_001108866	1	1	0	1	3
rno-miR-124-3p	MIMAT0000828	Pecr	113956	NM_133299	1	1	0	1	3
rno-miR-124-3p	MIMAT0000828	Slc25a37	306000	NM_001013996	1	1	0	1	3
rno-miR-124-3p	MIMAT0000828	Pcdha10	116778	NM_053939	1	1	0	1	3
rno-miR-124-3p	MIMAT0000828	Spire1	307348	NM_001107381	1	1	0	1	3
rno-miR-124-3p	MIMAT0000828	Adam17	57027	NM_020306	1	1	0	1	3
rno-miR-124-3p	MIMAT0000828	Tln1	313494	NM_001039025	1	1	0	1	3
rno-miR-124-3p	MIMAT0000828	Thop1	64517	NM_172075	1	1	0	1	3
rno-miR-124-3p	MIMAT0000828	Tsc22d4	684980	XM_003751150	1	1	0	1	3
rno-miR-124-3p	MIMAT0000828	Gja1	24392	NM_012567	1	1	0	1	3
rno-miR-124-3p	MIMAT0000828	Tmem219	308986	XM_003748988	1	1	0	1	3
rno-miR-124-3p	MIMAT0000828	Eno4	292138	NM_001134505	1	1	0	1	3
rno-miR-124-3p	MIMAT0000828	LOC1003	100360205	XM_002728399	1	1	0	1	3
rno-miR-124-3p	MIMAT0000828	LOC1003	100366121	XM_003750276	1	1	0	1	3
rno-miR-124-3p	MIMAT0000828	Upk1b	303924	NM_001024253	1	1	0	1	3
rno-miR-124-3p	MIMAT0000828	Manf	315989	NM_001108183	1	1	0	1	3
rno-miR-124-3p	MIMAT0000828	P4ha2	360526	NM_001108275	1	1	0	1	3
rno-miR-124-3p	MIMAT0000828	Gpr133	689257	XM_003752585	1	1	0	1	3
rno-miR-124-3p	MIMAT0000828	Spry1	294981	NM_001106427	1	1	0	1	3
rno-miR-124-3p	MIMAT0000828	Rab11fip1	498650	NM_001191555	1	1	0	1	3
rno-miR-124-3p	MIMAT0000828	Zbtb8os	297885	XM_003754126	1	1	0	1	3
rno-miR-124-3p	MIMAT0000828	Gart	288259	NM_001011899	1	1	0	1	3
rno-miR-124-3p	MIMAT0000828	Rassf1	363140	NM_001007754	1	1	0	1	3
rno-miR-124-3p	MIMAT0000828	Cd93	84398	NM_053383	1	1	0	1	3
rno-miR-124-3p	MIMAT0000828	Mrps23	360594	NM_001108289	1	1	0	1	3
rno-miR-124-3p	MIMAT0000828	Vstm4	361112	NM_001029920	1	1	0	1	3
rno-miR-124-3p	MIMAT0000828	Nfat5	307820	NM_001107425	1	1	0	1	3
rno-miR-124-3p	MIMAT0000828	Usp45	313098	NM_001107918	1	1	0	1	3
rno-miR-124-3p	MIMAT0000828	Rapgef1	63881	XM_001079347	1	1	0	1	3
rno-miR-124-3p	MIMAT0000828	Hs1bp3	313950	XM_233975	1	1	0	1	3
rno-miR-124-3p	MIMAT0000828	Pipox	303272	NM_001012009	1	1	0	1	3
rno-miR-124-3p	MIMAT0000828	Ddx26b	681665	XM_003752162	1	1	0	1	3
rno-miR-124-3p	MIMAT0000828	Cnksr3	308113	NM_001012061	1	1	0	1	3
rno-miR-124-3p	MIMAT0000828	RGD1564	290805	XM_002728414	1	1	0	1	3
rno-miR-124-3p	MIMAT0000828	Coq4	366013	NM_001031662	1	1	0	1	3
rno-miR-124-3p	MIMAT0000828	Palm	170673	NM_130829	1	1	0	1	3
rno-miR-124-3p	MIMAT0000828	LOC1003	100362345	XM_003749977	1	1	0	1	3
rno-miR-124-3p	MIMAT0000828	Zfand3	361816	NM_001012175	1	1	0	1	3
rno-miR-124-3p	MIMAT0000828	LOC1009	100910623	XM_003752297	1	1	0	1	3
rno-miR-124-3p	MIMAT0000828	Cyp2j4	65210	NM_023025	1	1	0	1	3
rno-miR-124-3p	MIMAT0000828	Ttc28	304558	XM_222260	1	1	0	1	3
rno-miR-124-3p	MIMAT0000828	Brwd3	317213	XM_001054667	1	1	0	1	3
rno-miR-124-3p	MIMAT0000828	Ephb4	686310	XM_003751156	1	1	0	1	3
rno-miR-124-3p	MIMAT0000828	Sh3bp5l	690898	NM_001127581	1	1	0	1	3
rno-miR-124-3p	MIMAT0000828	Capn1	29153	NM_019152	1	1	0	1	3
rno-miR-124-3p	MIMAT0000828	Rfk	499328	NM_001014106	1	1	0	1	3
rno-miR-124-3p	MIMAT0000828	LOC2987	298795	NM_001013941	1	1	0	1	3
rno-miR-124-3p	MIMAT0000828	Slc16a13	287451	NM_001005530	1	1	0	1	3
rno-miR-124-3p	MIMAT0000828	Rgl1	289080	NM_001105957	1	1	0	1	3
rno-miR-124-3p	MIMAT0000828	Egfl6	317470	NM_001108254	1	1	0	1	3
rno-miR-124-3p	MIMAT0000828	Ctnnd1	311163	NM_001107740	1	1	0	1	3
rno-miR-124-3p	MIMAT0000828	Serhl2	500911	NM_001130579	1	1	0	1	3
rno-miR-124-3p	MIMAT0000828	LOC1009	100911507	XM_003753707	1	1	0	1	3
rno-miR-124-3p	MIMAT0000828	Supt4h1	287608	NM_001105828	1	1	0	1	3
rno-miR-124-3p	MIMAT0000828	Best2	364973	NM_001108895	1	1	0	1	3
rno-miR-124-3p	MIMAT0000828	Slc12a2	83629	NM_031798	1	1	0	1	3
rno-miR-124-3p	MIMAT0000828	Sertad4	360899	NM_001108351	1	1	0	1	3
rno-miR-124-3p	MIMAT0000828	Fahd1	302980	NM_001024991	1	1	0	1	3
rno-miR-124-3p	MIMAT0000828	Osbpl8	314824	XM_003754284	1	1	0	1	3
rno-miR-124-3p	MIMAT0000828	Ccdc85a	289855	NM_001191553	1	1	0	1	3
rno-miR-124-3p	MIMAT0000828	RGD1306	309069	NM_001107557	1	1	0	1	3
rno-miR-124-3p	MIMAT0000828	RGD1307	292739	NM_001109664	1	1	0	1	3
rno-miR-124-3p	MIMAT0000828	Pcdha7	393089	NM_199507	1	1	0	1	3
rno-miR-124-3p	MIMAT0000828	Slc12a9	171443	NM_134405	1	1	0	1	3
rno-miR-124-3p	MIMAT0000828	Tp53inp2	362246	XM_001063973	1	1	0	1	3
rno-miR-124-3p	MIMAT0000828	Foxq1	64826	NM_022858	1	1	0	1	3
rno-miR-124-3p	MIMAT0000828	Cers4	304208	NM_001107117	1	1	0	1	3
rno-miR-124-3p	MIMAT0000828	Anxa9	689830	XM_002726022	1	1	0	1	3
rno-miR-124-3p	MIMAT0000828	Slc39a8	295455	NM_001011952	1	1	0	1	3

rno-miR-124-3p	MIMAT0000828	Col10a1	25681	XM_002725875	1	1	0	1	3
rno-miR-124-3p	MIMAT0000828	Ccdc3	498795	XM_574081	1	1	0	1	3
rno-miR-124-3p	MIMAT0000828	Bcat1	29592	NM_017253	1	1	0	1	3
rno-miR-124-3p	MIMAT0000828	Slc13a5	266998	NM_170668	1	1	0	1	3
rno-miR-124-3p	MIMAT0000828	LOC1009	100912032	XM_003752767	1	1	0	1	3
rno-miR-124-3p	MIMAT0000828	Plod3	288583	NM_178101	1	1	0	1	3
rno-miR-124-3p	MIMAT0000828	RGD1311	363276	NM_001134587	1	1	0	1	3
rno-miR-124-3p	MIMAT0000828	Dcakd	360639	NM_001007724	1	1	0	1	3
rno-miR-124-3p	MIMAT0000828	Bicd2	306809	NM_001033674	1	1	0	1	3
rno-miR-124-3p	MIMAT0000828	Svil	361256	XM_003751703	1	1	0	1	3
rno-miR-124-3p	MIMAT0000828	Furin	54281	NM_019331	1	1	0	1	3
rno-miR-124-3p	MIMAT0000828	Lrat	64047	NM_022280	1	1	0	1	3
rno-miR-124-3p	MIMAT0000828	Prpf39	314171	XM_003754180	1	1	0	1	3
rno-miR-124-3p	MIMAT0000828	Jam2	619374	NM_001034004	1	1	0	1	3
rno-miR-124-3p	MIMAT0000828	Mkx	291228	XM_001063892	1	1	0	1	3
rno-miR-124-3p	MIMAT0000828	Hectd2	309514	XM_003753393	1	1	0	1	3
rno-miR-124-3p	MIMAT0000828	Abcc4	170924	NM_133411	1	1	0	1	3
rno-miR-124-3p	MIMAT0000828	LOC1003	100363253	XM_002728891	1	1	0	1	3
rno-miR-124-3p	MIMAT0000828	Fbxl7	361907	NM_001108545	1	1	0	1	3
rno-miR-124-3p	MIMAT0000828	Ngef	246217	NM_001136241	1	1	0	1	3
rno-miR-124-3p	MIMAT0000828	Plekhm1	303584	NM_001009677	1	1	0	1	3
rno-miR-124-3p	MIMAT0000828	Rbm15	684233	XM_003749380	1	1	0	1	3
rno-miR-124-3p	MIMAT0000828	RGD1564	498269	XM_001075923	1	1	0	1	3
rno-miR-124-3p	MIMAT0000828	Rasd2	171099	NM_133568	1	1	0	1	3
rno-miR-124-3p	MIMAT0000828	Nktr	100364165	XM_003750608	1	1	0	1	3
rno-miR-124-3p	MIMAT0000828	Tstd2	362514	NM_001108663	1	1	0	1	3
rno-miR-124-3p	MIMAT0000828	Myo6	315840	XM_001061392	1	1	0	1	3
rno-miR-124-3p	MIMAT0000828	Tfap4	360482	NM_001108267	1	1	0	1	3
rno-miR-124-3p	MIMAT0000828	Fbxl13	100125363	NM_001103355	1	1	0	1	3
rno-miR-124-3p	MIMAT0000828	Nodal	294503	NM_001106394	1	1	0	1	3
rno-miR-124-3p	MIMAT0000828	RGD1560	499797	XM_001066238	1	1	0	1	3
rno-miR-124-3p	MIMAT0000828	Ssh3	365396	NM_001012217	1	1	0	1	3
rno-miR-124-3p	MIMAT0000828	Ache	83817	NM_172009	1	1	0	1	3
rno-miR-124-3p	MIMAT0000828	Ammecr1	307526	NM_001107399	1	1	0	1	3
rno-miR-124-3p	MIMAT0000828	Slc22a5	29726	NM_019269	1	1	0	1	3
rno-miR-124-3p	MIMAT0000828	Nxf7	501621	NM_001037216	1	1	0	1	3
rno-miR-124-3p	MIMAT0000828	Jrk	315073	NM_001104612	1	1	0	1	3
rno-miR-124-3p	MIMAT0000828	Serpine1	24617	NM_012620	1	1	0	1	3
rno-miR-124-3p	MIMAT0000828	Hnrnpul2	309197	XM_001073417	1	1	0	1	3
rno-miR-124-3p	MIMAT0000828	Cand2	192226	NM_181362	1	1	0	1	3
rno-miR-124-3p	MIMAT0000828	LOC1009	100910163	XM_003751622	1	1	0	1	3
rno-miR-124-3p	MIMAT0000828	Neo1	81735	XM_001074913	1	1	0	1	3
rno-miR-124-3p	MIMAT0000828	RGD1559	304447	XM_002727969	1	1	0	1	3
rno-miR-124-3p	MIMAT0000828	Antxr2	305633	XM_001069109	1	1	0	1	3
rno-miR-124-3p	MIMAT0000828	Adarb1	25367	NM_001111057	1	1	0	1	3
rno-miR-124-3p	MIMAT0000828	Cers2	310667	NM_001033700	1	1	0	1	3
rno-miR-124-3p	MIMAT0000828	Cpt1a	25757	NM_031559	1	1	0	1	3
rno-miR-124-3p	MIMAT0000828	Qk	499022	NM_001115021	1	1	0	1	3
rno-miR-124-3p	MIMAT0000828	Zfp213	287094	NM_001105764	1	1	0	1	3
rno-miR-124-3p	MIMAT0000828	LOC1009	100912447	XM_003751476	1	1	0	1	3
rno-miR-124-3p	MIMAT0000828	Dot1l	362831	NM_001108733	1	1	0	1	3
rno-miR-124-3p	MIMAT0000828	Gtlf3b	363614	NM_001170541	1	1	0	1	3
rno-miR-124-3p	MIMAT0000828	Ptprr	94202	NM_001113390	1	1	0	1	3
rno-miR-124-3p	MIMAT0000828	F2rl1	116677	NM_053897	1	1	0	1	3
rno-miR-124-3p	MIMAT0000828	Cbfb	361391	NM_001013191	1	1	0	1	3
rno-miR-124-3p	MIMAT0000828	Ankrd44	301415	NM_001191807	1	1	0	1	3
rno-miR-124-3p	MIMAT0000828	Blk	364403	NM_001025751	1	1	0	1	3
rno-miR-124-3p	MIMAT0000828	Pcdha12	116779	NM_053940	1	1	0	1	3
rno-miR-124-3p	MIMAT0000828	Car9	313495	NM_001107956	1	1	0	1	3
rno-miR-124-3p	MIMAT0000828	Entpd1	64519	XM_003749121	1	1	0	1	3
rno-miR-124-3p	MIMAT0000828	RGD1311	289568	XM_002724955	1	1	0	1	3
rno-miR-124-3p	MIMAT0000828	Tmem219	308986	XM_003753336	1	1	0	1	3
rno-miR-124-3p	MIMAT0000828	Wdr92	498418	NM_001127559	1	1	0	1	3
rno-miR-124-3p	MIMAT0000828	LOC1003	100360205	XM_003752899	1	1	0	1	3
rno-miR-124-3p	MIMAT0000828	Myo9a	171296	NM_134335	1	1	0	1	3
rno-miR-124-3p	MIMAT0000828	Fzd4	64558	NM_022623	1	1	0	1	3
rno-miR-124-3p	MIMAT0000828	Mapkapk3	315994	NM_001012127	1	1	0	1	3
rno-miR-124-3p	MIMAT0000828	Wdfy3	305164	NM_001170551	1	1	0	1	3
rno-miR-124-3p	MIMAT0000828	Trim11	360534	NM_001108276	1	1	0	1	3

rno-miR-124-3p	MIMAT0000828	Spg20	295053	NM_001106433	1	1	0	1	3
rno-miR-124-3p	MIMAT0000828	Rab11fip1	498650	NM_001197241	1	1	0	1	3
rno-miR-124-3p	MIMAT0000828	Gdap1	312890	NM_001107897	1	1	0	1	3
rno-miR-124-3p	MIMAT0000828	Carhsp1	260416	NM_152790	1	1	0	1	3
rno-miR-124-3p	MIMAT0000828	LOC1009	100911911	XM_003748820	1	1	0	1	3
rno-miR-124-3p	MIMAT0000828	Camta1	362665	NM_001195559	1	1	0	1	3
rno-miR-124-3p	MIMAT0000828	Itga3	360606	XM_003750907	1	1	0	1	3
rno-miR-124-3p	MIMAT0000828	Rfc2	116468	NM_053786	1	1	0	1	3
rno-miR-124-3p	MIMAT0000828	Tmco3	306607	XM_003751630	1	1	0	1	3
rno-miR-124-3p	MIMAT0000828	Rab27a	50645	NM_017317	1	1	0	1	3
rno-miR-124-3p	MIMAT0000828	Rapgef1	63881	XM_216018	1	1	0	1	3
rno-miR-124-3p	MIMAT0000828	Smc6	313961	NM_001108014	1	1	0	1	3
rno-miR-124-3p	MIMAT0000828	Eya1	502935	XM_001059758	1	1	0	1	3
rno-miR-124-3p	MIMAT0000828	RGD1562	290818	NM_001100944	1	1	0	1	3
rno-miR-124-3p	MIMAT0000828	Sfrp5	309377	NM_001107591	1	1	0	1	3
rno-miR-124-3p	MIMAT0000828	LOC1003	100362345	XM_003754058	1	1	0	1	3
rno-miR-124-3p	MIMAT0000828	Muc13	207126	XM_003751061	1	1	0	1	3
rno-miR-124-3p	MIMAT0000828	LOC1009	100910638	XM_003751414	1	1	0	1	3
rno-miR-124-3p	MIMAT0000828	Nlrx1	315599	NM_001025010	1	1	0	1	3
rno-miR-124-3p	MIMAT0000828	Pard3	81918	NM_031235	1	1	0	1	3
rno-miR-124-3p	MIMAT0000828	Galnt9	304571	NM_001107151	1	1	0	1	3
rno-miR-124-3p	MIMAT0000828	Brwd3	317213	XM_228518	1	1	0	1	3
rno-miR-124-3p	MIMAT0000828	Ephb4	686310	XM_003751157	1	1	0	1	3
rno-miR-124-3p	MIMAT0000828	Luc7l2	312251	NM_001107853	1	1	0	1	3
rno-miR-124-3p	MIMAT0000828	Slc35f6	298851	NM_001017451	1	1	0	1	3
rno-miR-124-3p	MIMAT0000828	Cdca4	500727	NM_001037214	1	1	0	1	3
rno-miR-124-3p	MIMAT0000828	LOC1009	100910929	XM_003749059	1	1	0	1	3
rno-miR-124-3p	MIMAT0000828	Alox12	287454	NM_001105798	1	1	0	1	3
rno-miR-124-3p	MIMAT0000828	Aplnr	83518	NM_031349	1	1	0	1	3
rno-miR-124-3p	MIMAT0000828	Fpgs	687266	NM_001146125	1	1	0	1	3
rno-miR-124-3p	MIMAT0000828	Fam211a	691777	XM_001079602	1	1	0	1	3
rno-miR-124-3p	MIMAT0000828	RT1-DMb	294273	NM_198740	1	1	0	1	3
rno-miR-124-3p	MIMAT0000828	Pam	25508	NM_013000	1	1	0	1	3
rno-miR-124-3p	MIMAT0000828	Stom	296655	NM_001011965	1	1	0	1	3
rno-miR-124-3p	MIMAT0000828	Ftl	29292	NM_022500	1	1	0	1	3
rno-miR-124-3p	MIMAT0000828	Zfyve20	312562	NM_001107875	1	1	0	1	3
rno-miR-124-3p	MIMAT0000828	Rfxap	499617	NM_001044263	1	1	0	1	3
rno-miR-124-3p	MIMAT0000828	LOC1009	100911546	XM_003752180	1	1	0	1	3
rno-miR-124-3p	MIMAT0000828	Vezf1	287615	XM_001081191	1	1	0	1	3
rno-miR-124-3p	MIMAT0000828	Prr13	363004	NM_001008379	1	1	0	1	3
rno-miR-124-3p	MIMAT0000828	Gcdh	364975	NM_001108896	1	1	0	1	3
rno-miR-124-3p	MIMAT0000828	Dennd6a	306229	NM_001134467	1	1	0	1	3
rno-miR-124-3p	MIMAT0000828	Relt	361615	NM_001108495	1	1	0	1	3
rno-miR-124-3p	MIMAT0000828	Unkl	302987	XM_001059160	1	1	0	1	3
rno-miR-124-3p	MIMAT0000828	Osbpl8	314824	XM_003754285	1	1	0	1	3
rno-miR-124-3p	MIMAT0000828	Oraov1	309136	NM_001107565	1	1	0	1	3
rno-miR-124-3p	MIMAT0000828	Snx18	310097	NM_001107652	1	1	0	1	3
rno-miR-124-3p	MIMAT0000828	Pcdha9	393090	NM_199508	1	1	0	1	3
rno-miR-124-3p	MIMAT0000828	Ipmk	171458	NM_134417	1	1	0	1	3
rno-miR-124-3p	MIMAT0000828	LOC1009	100909758	XM_003751175	1	1	0	1	3
rno-miR-124-3p	MIMAT0000828	Tp53inp2	362246	XM_003749598	1	1	0	1	3
rno-miR-124-3p	MIMAT0000828	Emr1	316137	NM_001007557	1	1	0	1	3
rno-miR-124-3p	MIMAT0000828	Cdca7	311742	NM_001025693	1	1	0	1	3
rno-miR-124-3p	MIMAT0000828	Slc35d1	298280	NM_001106668	1	1	0	1	3
rno-miR-124-3p	MIMAT0000828	Pax9	362741	NM_001039539	1	1	0	1	3
rno-miR-124-3p	MIMAT0000828	Ajuba	85265	NM_053503	1	1	0	1	3
rno-miR-124-3p	MIMAT0000828	Limd2	360646	NM_001025715	1	1	0	1	3
rno-miR-124-3p	MIMAT0000828	Bicd2	306809	NM_198765	1	1	0	1	3
rno-miR-124-3p	MIMAT0000828	Svil	361256	XM_003752990	1	1	0	1	3
rno-miR-124-3p	MIMAT0000828	Fyco1	301085	NM_001106870	1	1	0	1	3
rno-miR-124-3p	MIMAT0000828	Agt	24179	NM_134432	1	1	0	1	3
rno-miR-124-3p	MIMAT0000828	Pvrl2	308417	NM_001012064	1	1	0	1	3
rno-miR-124-3p	MIMAT0000828	Sorcs1	309533	XM_001070775	1	1	0	1	3
rno-miR-124-3p	MIMAT0000828	Sptlc2	366697	NM_001037097	1	1	0	1	3
rno-miR-124-3p	MIMAT0000828	Yeats2	498112	NM_001109057	1	1	0	1	3
rno-miR-124-3p	MIMAT0000828	Slc25a10	170943	NM_133418	1	1	0	1	3
rno-miR-124-3p	MIMAT0000828	LOC1003	100363253	XM_003753419	1	1	0	1	3
rno-miR-124-3p	MIMAT0000828	Arhgap4	246249	NM_144740	1	1	0	1	3
rno-miR-124-3p	MIMAT0000828	Sumf1	362409	NM_001108639	1	1	0	1	3

rno-miR-124-3p	MIMAT0000828	Ptdss1	314553	NM_001012113	1	1	0	1	3
rno-miR-124-3p	MIMAT0000828	LOC6787	678766	XM_001053087	1	1	0	1	3
rno-miR-124-3p	MIMAT0000828	Psg19	24256	NM_019126	1	1	0	1	3
rno-miR-124-3p	MIMAT0000828	Tsku	308843	NM_001009965	1	1	0	1	3
rno-miR-124-3p	MIMAT0000828	RGD1564	498269	XM_002728030	1	1	0	1	3
rno-miR-124-3p	MIMAT0000828	Zfp420	100359464	XM_002728737	1	1	0	1	3
rno-miR-124-3p	MIMAT0000828	Nktr	100364165	XM_003754480	1	1	0	1	3
rno-miR-124-3p	MIMAT0000828	Myo6	315840	XM_236444	1	1	0	1	3
rno-miR-124-3p	MIMAT0000828	Fam20b	304885	NM_001107187	1	1	0	1	3
rno-miR-124-3p	MIMAT0000828	Rundc3b	688590	NM_001047116	1	1	0	1	3
rno-miR-124-3p	MIMAT0000828	LOC1001	100125367	NM_001103357	1	1	0	1	3
rno-miR-124-3p	MIMAT0000828	Rasgef1a	312664	XM_232315	1	1	0	1	3
rno-miR-124-3p	MIMAT0000828	RGD1560	499797	XM_575134	1	1	0	1	3
rno-miR-124-3p	MIMAT0000828	Glce	363073	XM_001073932	1	1	0	1	3
rno-miR-124-3p	MIMAT0000828	Osbp	365410	NM_001108927	1	1	0	1	3
rno-miR-124-3p	MIMAT0000828	Spock3	306404	NM_001107310	1	1	0	1	3
rno-miR-124-3p	MIMAT0000828	Eci1	29740	NM_017306	1	1	0	1	3
rno-miR-124-3p	MIMAT0000828	Fam122b	501647	NM_001166586	1	1	0	1	3
rno-miR-124-3p	MIMAT0000828	Cbx6	315136	NM_001012119	1	1	0	1	3
rno-miR-124-3p	MIMAT0000828	Hnrnpul2	309197	XM_219560	1	1	0	1	3
rno-miR-124-3p	MIMAT0000828	Trim55	365751	NM_001012218	1	1	0	1	3
rno-miR-124-3p	MIMAT0000828	Il33	361749	NM_001014166	1	1	0	1	3
rno-miR-124-3p	MIMAT0000828	LOC1009	100910212	XM_003748699	1	1	0	1	3
rno-miR-124-3p	MIMAT0000828	Scamp2	65168	NM_023955	1	1	0	1	3
rno-miR-124-3p	MIMAT0000828	Hoxc5	315341	NM_001108116	1	1	0	1	3
rno-miR-124-3p	MIMAT0000828	Neo1	81735	XM_003750526	1	1	0	1	3
rno-miR-124-3p	MIMAT0000828	Rilpl1	304469	NM_001191665	1	1	0	1	3
rno-miR-124-3p	MIMAT0000828	Tmbim1	316516	NM_001007713	1	1	0	1	3
rno-miR-124-3p	MIMAT0000828	Antxr2	305633	XM_223745	1	1	0	1	3
rno-miR-124-3p	MIMAT0000828	Prima1	690195	NM_001108721	1	1	0	1	3
rno-miR-124-3p	MIMAT0000828	Adarb1	25367	NM_012894	1	1	0	1	3
rno-miR-124-3p	MIMAT0000828	Mul1	298576	NM_001106695	1	1	0	1	3
rno-miR-124-3p	MIMAT0000828	Tmem51	500578	NM_001109273	1	1	0	1	3
rno-miR-124-3p	MIMAT0000828	Kctd5	287109	NM_001105768	1	1	0	1	3
rno-miR-124-3p	MIMAT0000828	LOC1009	100912447	XM_003752809	1	1	0	1	3
rno-miR-124-3p	MIMAT0000828	Rfx1	288906	NM_001105944	1	1	0	1	3
rno-miR-124-3p	MIMAT0000828	Ptprr	94202	NM_053594	1	1	0	1	3
rno-miR-124-3p	MIMAT0000828	Wdr37	307075	NM_001107362	1	1	0	1	3
rno-miR-124-3p	MIMAT0000828	Ranbp10	361396	NM_001135875	1	1	0	1	3
rno-miR-124-3p	MIMAT0000828	Rassf3	362886	NM_001108747	1	1	0	1	3
rno-miR-124-3p	MIMAT0000828	Hmgn3	113990	NM_001007020	1	1	0	1	3
rno-miR-124-3p	MIMAT0000828	Pcdha3	116780	NM_053941	1	1	0	1	3
rno-miR-124-3p	MIMAT0000828	Nt5e	58813	NM_021576	1	1	0	1	3
rno-miR-124-3p	MIMAT0000828	Esyt2	299488	XM_002726849	1	1	0	1	3
rno-miR-124-3p	MIMAT0000828	Tal1	313507	NM_001107958	1	1	0	1	3
rno-miR-124-3p	MIMAT0000828	Ech1	64526	NM_022594	1	1	0	1	3
rno-miR-124-3p	MIMAT0000828	Ubn1	302935	NM_001106977	1	1	0	1	3
rno-miR-124-3p	MIMAT0000828	Chst11	314694	NM_001108079	1	1	0	1	3
rno-miR-124-3p	MIMAT0000828	Tspan15	679462	NM_001115032	1	1	0	1	3
rno-miR-124-3p	MIMAT0000828	RGD1311	289568	XM_002728094	1	1	0	1	3
rno-miR-124-3p	MIMAT0000828	Tmem219	308986	XM_219337	1	1	0	1	3
rno-miR-124-3p	MIMAT0000828	Rtn4ip1	309912	NM_001107644	1	1	0	1	3
rno-miR-124-3p	MIMAT0000828	Ankib1	368062	NM_001134781	1	1	0	1	3
rno-miR-124-3p	MIMAT0000828	Sema3f	315996	NM_001108185	1	1	0	1	3
rno-miR-124-3p	MIMAT0000828	Shroom3	305230	NM_001100889	1	1	0	1	3
rno-miR-124-3p	MIMAT0000828	Efnb3	360546	NM_001100980	1	1	0	1	3
rno-miR-124-3p	MIMAT0000828	Slc7a1	25648	NM_013111	1	1	0	1	3
rno-miR-124-3p	MIMAT0000828	Sdcbp2	311532	NM_001025692	1	1	0	1	3
rno-miR-124-3p	MIMAT0000828	Smim8	297971	NM_001106644	1	1	0	1	3
rno-miR-124-3p	MIMAT0000828	Rb1cc1	312927	NM_001107901	1	1	0	1	3
rno-miR-124-3p	MIMAT0000828	Aurka	261730	NM_153296	1	1	0	1	3
rno-miR-124-3p	MIMAT0000828	Wdr48	363164	NM_001135895	1	1	0	1	3
rno-miR-124-3p	MIMAT0000828	Qsox1	84491	NM_001109898	1	1	0	1	3
rno-miR-124-3p	MIMAT0000828	Itga3	360606	XM_003752369	1	1	0	1	3
rno-miR-124-3p	MIMAT0000828	Arhgap17	63994	NM_022244	1	1	0	1	3
rno-miR-124-3p	MIMAT0000828	Reep2	682105	NM_001048047	1	1	0	1	3
rno-miR-124-3p	MIMAT0000828	Golga7b	309378	XM_001055463	1	1	0	1	3
rno-miR-124-3p	MIMAT0000828	Slc40a1	170840	NM_133315	1	1	0	1	3
rno-miR-124-3p	MIMAT0000828	LOC1003	100362384	XM_002728041	1	1	0	1	3

rno-miR-124-3p	MIMAT0000828	Kmt2a	315606	XM_003750506	1	1	0	1	3
rno-miR-124-3p	MIMAT0000828	Fut1	81919	NM_031236	1	1	0	1	3
rno-miR-124-3p	MIMAT0000828	Galnt9	304571	NM_001122644	1	1	0	1	3
rno-miR-124-3p	MIMAT0000828	Ephb4	686310	XM_003752575	1	1	0	1	3
rno-miR-124-3p	MIMAT0000828	Gys1	690987	NM_001109615	1	1	0	1	3
rno-miR-124-3p	MIMAT0000828	Gss	25458	NM_012962	1	1	0	1	3
rno-miR-124-3p	MIMAT0000828	LOC3122	312273	NM_001107856	1	1	0	1	3
rno-miR-124-3p	MIMAT0000828	Fam178a	499360	NM_001134612	1	1	0	1	3
rno-miR-124-3p	MIMAT0000828	Pqlc3	298906	NM_001034952	1	1	0	1	3
rno-miR-124-3p	MIMAT0000828	Tmem198	500762	NM_001109281	1	1	0	1	3
rno-miR-124-3p	MIMAT0000828	LOC1009	100911029	XM_003751391	1	1	0	1	3
rno-miR-124-3p	MIMAT0000828	Smurf2	303614	NM_001107061	1	1	0	1	3
rno-miR-124-3p	MIMAT0000828	Cln6	315746	NM_001191794	1	1	0	1	3
rno-miR-124-3p	MIMAT0000828	Pafah1b1	83572	NM_031763	1	1	0	1	3
rno-miR-124-3p	MIMAT0000828	Epb4.1l5	304733	NM_001012023	1	1	0	1	3
rno-miR-124-3p	MIMAT0000828	Fam211a	691777	XM_002724616	1	1	0	1	3
rno-miR-124-3p	MIMAT0000828	Pemt	25511	NM_013003	1	1	0	1	3
rno-miR-124-3p	MIMAT0000828	Lhx2	296706	NM_001106571	1	1	0	1	3
rno-miR-124-3p	MIMAT0000828	Gata6	29300	NM_019185	0	1	1	1	3
rno-miR-124-3p	MIMAT0000828	Tmem117	500921	XM_576330	1	1	0	1	3
rno-miR-124-3p	MIMAT0000828	Frmd4b	252858	XM_001077268	1	1	0	1	3
rno-miR-124-3p	MIMAT0000828	LOC1009	100911597	XM_003750378	1	1	0	1	3
rno-miR-124-3p	MIMAT0000828	Vezf1	287615	XM_213421	1	1	0	1	3
rno-miR-124-3p	MIMAT0000828	Endod1	363015	XM_002726985	1	1	0	1	3
rno-miR-124-3p	MIMAT0000828	Capn6	83685	NM_031808	1	1	0	1	3
rno-miR-124-3p	MIMAT0000828	Nrip3	361625	NM_001108498	1	1	0	1	3
rno-miR-124-3p	MIMAT0000828	Unkl	302987	XM_220238	1	1	0	1	3
rno-miR-124-3p	MIMAT0000828	Frs2	314850	NM_001108097	1	1	0	1	3
rno-miR-124-3p	MIMAT0000828	Tet3	680576	XM_001057850	1	1	0	1	3
rno-miR-124-3p	MIMAT0000828	Tpcn2	309139	NM_001107566	1	1	0	1	3
rno-miR-124-3p	MIMAT0000828	Psmd8	292766	NM_001100831	1	1	0	1	3
rno-miR-124-3p	MIMAT0000828	Aqp1	25240	NM_012778	1	1	0	1	3
rno-miR-124-3p	MIMAT0000828	Pcdhac1	393091	NM_199509	1	1	0	1	3
rno-miR-124-3p	MIMAT0000828	Cd99l2	171485	NM_134459	1	1	0	1	3
rno-miR-124-3p	MIMAT0000828	LOC1009	100909758	XM_003752605	1	1	0	1	3
rno-miR-124-3p	MIMAT0000828	Tp53inp2	362246	XM_003753814	1	1	0	1	3
rno-miR-124-3p	MIMAT0000828	Usp49	316211	NM_001136470	1	1	0	1	3
rno-miR-124-3p	MIMAT0000828	LOC6857	685707	XM_003751240	1	1	0	1	3
rno-miR-124-3p	MIMAT0000828	Dok7	305448	NM_001130062	1	1	0	1	3
rno-miR-124-3p	MIMAT0000828	Htr5a	25689	NM_013148	1	1	0	1	3
rno-miR-124-3p	MIMAT0000828	RGD1561	298320	XM_001062216	1	1	0	1	3
rno-miR-124-3p	MIMAT0000828	Ppif	282819	NM_172243	1	1	0	1	3
rno-miR-124-3p	MIMAT0000828	Zfp609	363412	NM_001173371	1	1	0	1	3
rno-miR-124-3p	MIMAT0000828	Sos2	85384	NM_001135561	1	1	0	1	3
rno-miR-124-3p	MIMAT0000828	Mif4gd	360659	NM_001014122	1	1	0	1	3
rno-miR-124-3p	MIMAT0000828	Vwa5a	301097	NM_198755	1	1	0	1	3
rno-miR-124-3p	MIMAT0000828	Ncoa4	619385	NM_001034007	1	1	0	1	3
rno-miR-124-3p	MIMAT0000828	LOC3034	303448	NM_001037190	1	1	0	1	3
rno-miR-124-3p	MIMAT0000828	Uqcc	683512	NM_001109446	1	1	0	1	3
rno-miR-124-3p	MIMAT0000828	Ak2	24184	NM_001033967	1	1	0	1	3
rno-miR-124-3p	MIMAT0000828	Samd4b	308473	NM_001107498	1	1	0	1	3
rno-miR-124-3p	MIMAT0000828	Zadh2	291403	NM_001106129	1	1	0	1	3
rno-miR-124-3p	MIMAT0000828	Bcl2l1	24888	NM_001033672	1	1	0	1	3
rno-miR-124-3p	MIMAT0000828	RGD1565	366733	XM_001071789	1	1	0	1	3
rno-miR-124-3p	MIMAT0000828	Zfp275	293849	NM_001106343	1	1	0	1	3
rno-miR-124-3p	MIMAT0000828	Marf1	170946	NM_133421	1	1	0	1	3
rno-miR-124-3p	MIMAT0000828	LOC1003	100363275	XM_003751617	1	1	0	1	3
rno-miR-124-3p	MIMAT0000828	Trib3	246273	NM_144755	1	1	0	1	3
rno-miR-124-3p	MIMAT0000828	LOC6787	678766	XM_003751895	1	1	0	1	3
rno-miR-124-3p	MIMAT0000828	Chrnb1	24261	NM_012528	1	1	0	1	3
rno-miR-124-3p	MIMAT0000828	Foxl2	367152	XM_003750571	1	1	0	1	3
rno-miR-124-3p	MIMAT0000828	LOC1003	100359668	XM_002726740	1	1	0	1	3
rno-miR-124-3p	MIMAT0000828	LOC1003	100364699	XM_002725099	1	1	0	1	3
rno-miR-124-3p	MIMAT0000828	Lmo4	362051	NM_001009708	1	1	0	1	3
rno-miR-124-3p	MIMAT0000828	Smc2	362519	NM_001108666	1	1	0	1	3
rno-miR-124-3p	MIMAT0000828	Phip	315843	XM_003750551	1	1	0	1	3
rno-miR-124-3p	MIMAT0000828	LOC6886	688613	NM_001134845	1	1	0	1	3
rno-miR-124-3p	MIMAT0000828	Aes	29466	NM_019220	1	1	0	1	3
rno-miR-124-3p	MIMAT0000828	Frmd6	257646	XM_001080473	1	1	0	1	3

rno-miR-124-3p	MIMAT0000828	Ccdc40	287867	NM_001134688	1	1	0	1	3
rno-miR-124-3p	MIMAT0000828	Glce	363073	XM_343404	1	1	0	1	3
rno-miR-124-3p	MIMAT0000828	Sdcbp	83841	NM_031986	1	1	0	1	3
rno-miR-124-3p	MIMAT0000828	Slc25a1	29743	NM_017307	1	1	0	1	3
rno-miR-124-3p	MIMAT0000828	Amot	300289	XM_001056974	1	1	0	1	3
rno-miR-124-3p	MIMAT0000828	Tmem114	501675	NM_001134638	1	1	0	1	3
rno-miR-124-3p	MIMAT0000828	Top3a	303194	XM_001077625	1	1	0	1	3
rno-miR-124-3p	MIMAT0000828	Tab1	315139	NM_001109976	1	1	0	1	3
rno-miR-124-3p	MIMAT0000828	Gna14	309242	NM_001013151	1	1	0	1	3
rno-miR-124-3p	MIMAT0000828	Fstl5	365823	XM_003749307	1	1	0	1	3
rno-miR-124-3p	MIMAT0000828	Fam198b	310540	NM_199105	1	1	0	1	3
rno-miR-124-3p	MIMAT0000828	LOC1009	100910212	XM_003753178	1	1	0	1	3
rno-miR-124-3p	MIMAT0000828	Scamp4	65170	NM_031725	1	1	0	1	3
rno-miR-124-3p	MIMAT0000828	Copz1	315345	NM_001108117	1	1	0	1	3
rno-miR-124-3p	MIMAT0000828	Dlg5	305645	XM_003751423	1	1	0	1	3
rno-miR-124-3p	MIMAT0000828	Bcan	25393	NM_012916	1	1	0	1	3
rno-miR-124-3p	MIMAT0000828	Rcc2	298594	XM_001072061	1	1	0	1	3
rno-miR-124-3p	MIMAT0000828	Socs5	500616	NM_001109274	1	1	0	1	3
rno-miR-124-3p	MIMAT0000828	Rpusd1	287148	NM_001105774	1	1	0	1	3
rno-miR-124-3p	MIMAT0000828	LOC1009	100912493	XM_003750105	1	1	0	1	3
rno-miR-124-3p	MIMAT0000828	Farsa	288917	NM_001024237	1	1	0	1	3
rno-miR-124-3p	MIMAT0000828	Pan3	360760	XM_001068449	1	1	0	1	3
rno-miR-124-3p	MIMAT0000828	Edc4	361399	NM_001033068	1	1	0	1	3
rno-miR-124-3p	MIMAT0000828	Foxn1	287469	NM_001100648	1	1	0	1	3
rno-miR-124-3p	MIMAT0000828	Nipal2	362899	NM_001130559	1	1	0	1	3
rno-miR-124-3p	MIMAT0000828	Egr2	114090	NM_053633	1	1	0	1	3
rno-miR-124-3p	MIMAT0000828	Zranb2	58821	NM_031616	1	1	0	1	3
rno-miR-124-3p	MIMAT0000828	Esyt2	299488	XM_002729743	1	1	0	1	3
rno-miR-124-3p	MIMAT0000828	Elovl1	679532	NM_001044275	1	1	0	1	3
rno-miR-124-3p	MIMAT0000828	Zfp668	309002	NM_001107553	1	1	0	1	3
rno-miR-124-3p	MIMAT0000828	Thbs2	292406	NM_001169138	1	1	0	1	3
rno-miR-124-3p	MIMAT0000828	Stat3	25125	NM_012747	1	1	0	1	3
rno-miR-124-3p	MIMAT0000828	Chrd	117275	NM_057134	1	1	0	1	3
rno-miR-124-3p	MIMAT0000828	Foxc2	171356	NM_001101680	1	1	0	1	3
rno-miR-124-3p	MIMAT0000828	LOC1009	100909455	XM_003748889	1	1	0	1	3
rno-miR-124-3p	MIMAT0000828	Tubgcp4	362203	XM_003749561	1	1	0	1	3
rno-miR-124-3p	MIMAT0000828	Rarg	685072	NM_001135249	1	1	0	1	3
rno-miR-124-3p	MIMAT0000828	Art3	305235	NM_001012034	1	1	0	1	3
rno-miR-124-3p	MIMAT0000828	Plscr3	360549	NM_001012139	1	1	0	1	3
rno-miR-124-3p	MIMAT0000828	Ptgfr	25652	NM_013115	1	1	0	1	3
rno-miR-124-3p	MIMAT0000828	Asxl1	311553	XM_002726270	1	1	0	1	3
rno-miR-124-3p	MIMAT0000828	Smim8	297971	NM_001201373	1	1	0	1	3
rno-miR-124-3p	MIMAT0000828	Tmem68	312946	NM_001107903	1	1	0	1	3
rno-miR-124-3p	MIMAT0000828	Clrn1	261738	NM_153299	1	1	0	1	3
rno-miR-124-3p	MIMAT0000828	Zfp167	363170	NM_001170577	1	1	0	1	3
rno-miR-124-3p	MIMAT0000828	Qsox1	84491	NM_053431	1	1	0	1	3
rno-miR-124-3p	MIMAT0000828	Wnk1	116477	NM_001002823	1	1	0	1	3
rno-miR-124-3p	MIMAT0000828	RGD1359	313155	NM_001007702	1	1	0	1	3
rno-miR-124-3p	MIMAT0000828	Dusp10	63995	NM_001105734	1	1	0	1	3
rno-miR-124-3p	MIMAT0000828	Tmem229	503035	NM_001109359	1	1	0	1	3
rno-miR-124-3p	MIMAT0000828	Hic1	303310	NM_001107021	1	1	0	1	3
rno-miR-124-3p	MIMAT0000828	Tmem234	682404	XM_001061367	1	1	0	1	3
rno-miR-124-3p	MIMAT0000828	Lats1	308265	XM_003748644	1	1	0	1	3
rno-miR-124-3p	MIMAT0000828	Slc9a4	24785	NM_173098	1	1	0	1	3
rno-miR-124-3p	MIMAT0000828	Golga7b	309378	XM_219889	1	1	0	1	3
rno-miR-124-3p	MIMAT0000828	Esf1	366203	NM_001100771	1	1	0	1	3
rno-miR-124-3p	MIMAT0000828	RGD1305	293500	NM_001013901	1	1	0	1	3
rno-miR-124-3p	MIMAT0000828	Tmem88	497936	NM_001128155	1	1	0	1	3
rno-miR-124-3p	MIMAT0000828	Tmem150	245966	NM_139107	1	1	0	1	3
rno-miR-124-3p	MIMAT0000828	Kmt2a	315606	XM_003754423	1	1	0	1	3
rno-miR-124-3p	MIMAT0000828	Tbl2	686610	XM_001071365	1	1	0	1	3
rno-miR-124-3p	MIMAT0000828	Ctsj	29174	NM_017121	1	1	0	1	3
rno-miR-124-3p	MIMAT0000828	RGD1560	500804	XM_001078366	1	1	0	1	3
rno-miR-124-3p	MIMAT0000828	Crebl2	362453	NM_001015027	1	1	0	1	3
rno-miR-124-3p	MIMAT0000828	Nr4a1	79240	NM_024388	1	1	0	1	3
rno-miR-124-3p	MIMAT0000828	Btbd17	303660	NM_001134534	1	1	0	1	3
rno-miR-124-3p	MIMAT0000828	Parp16	315760	NM_001014093	1	1	0	1	3
rno-miR-124-3p	MIMAT0000828	Il17d	691799	XM_001079675	1	1	0	1	3
rno-miR-124-3p	MIMAT0000828	Tp53i11	311209	NM_001107749	1	1	0	1	3

rno-miR-124-3p	MIMAT0000828	Adamts9	312566	NM_001107877	1	1	0	1	3
rno-miR-124-3p	MIMAT0000828	Frmd4b	252858	XM_003749804	1	1	0	1	3
rno-miR-124-3p	MIMAT0000828	LOC1009	100911615	XM_003749014	1	1	0	1	3
rno-miR-124-3p	MIMAT0000828	Tom1l1	287622	NM_001253859	1	1	0	1	3
rno-miR-124-3p	MIMAT0000828	Endod1	363015	XM_002729871	1	1	0	1	3
rno-miR-124-3p	MIMAT0000828	Eaf1	306261	NM_001107293	1	1	0	1	3
rno-miR-124-3p	MIMAT0000828	Negr1	59318	NM_021682	1	1	0	1	3
rno-miR-124-3p	MIMAT0000828	Sox8	302993	NM_001106989	1	1	0	1	3
rno-miR-124-3p	MIMAT0000828	Tet3	680576	XM_002726424	1	1	0	1	3
rno-miR-124-3p	MIMAT0000828	Dhrs1	290234	NM_001007621	1	1	0	1	3
rno-miR-124-3p	MIMAT0000828	Etfb	292845	NM_001004220	1	1	0	1	3
rno-miR-124-3p	MIMAT0000828	Arnt2	25243	NM_012781	1	1	0	1	3
rno-miR-124-3p	MIMAT0000828	Pcdhac2	393092	NM_201422	1	1	0	1	3
rno-miR-124-3p	MIMAT0000828	Prpf18	171552	NM_138523	1	1	0	1	3
rno-miR-124-3p	MIMAT0000828	Rnf216	304294	NM_001107122	1	1	0	1	3
rno-miR-124-3p	MIMAT0000828	LOC6857	685707	XM_003752641	1	1	0	1	3
rno-miR-124-3p	MIMAT0000828	Ide	25700	NM_013159	1	1	0	1	3
rno-miR-124-3p	MIMAT0000828	Crat	311849	NM_001004085	1	1	0	1	3
rno-miR-124-3p	MIMAT0000828	RGD1561	298320	XM_233277	1	1	0	1	3
rno-miR-124-3p	MIMAT0000828	Sspn	500364	NM_001109255	1	1	0	1	3
rno-miR-124-3p	MIMAT0000828	Tac4	282829	NM_172328	1	1	0	1	3
rno-miR-124-3p	MIMAT0000828	Dhx37	288647	NM_001105926	1	1	0	1	3
rno-miR-124-3p	MIMAT0000828	Shc1	85385	NM_001164060	1	1	0	1	3
rno-miR-124-3p	MIMAT0000828	Sap30bp	360662	NM_001108305	1	1	0	1	3
rno-miR-124-3p	MIMAT0000828	Hspa12a	307997	NM_001107445	1	1	0	1	3
rno-miR-124-3p	MIMAT0000828	Eif2ak2	54287	NM_019335	1	1	0	1	3
rno-miR-124-3p	MIMAT0000828	Trim14	313236	NM_001107934	1	1	0	1	3
rno-miR-124-3p	MIMAT0000828	Plekhh1	314262	NM_001108036	1	1	0	1	3
rno-miR-124-3p	MIMAT0000828	Ncoa4	619385	NM_001034008	1	1	0	1	3
rno-miR-124-3p	MIMAT0000828	LOC6836	683674	XM_001064018	1	1	0	1	3
rno-miR-124-3p	MIMAT0000828	Alpi	24197	NM_022665	1	1	0	1	3
rno-miR-124-3p	MIMAT0000828	Bcl2l1	24888	NM_031535	1	1	0	1	3
rno-miR-124-3p	MIMAT0000828	RGD1565	366733	XM_345725	1	1	0	1	3
rno-miR-124-3p	MIMAT0000828	Srpk3	293854	NM_184045	1	1	0	1	3
rno-miR-124-3p	MIMAT0000828	Gpr146	498153	NM_001109062	1	1	0	1	3
rno-miR-124-3p	MIMAT0000828	Marf1	170946	XM_003752289	1	1	0	1	3
rno-miR-124-3p	MIMAT0000828	LOC1003	100363275	XM_003752918	1	1	0	1	3
rno-miR-124-3p	MIMAT0000828	Dmrta1	313352	NM_001107945	1	1	0	1	3
rno-miR-124-3p	MIMAT0000828	Itm2c	301575	NM_001009674	1	1	0	1	3
rno-miR-124-3p	MIMAT0000828	Zfp763	314586	NM_001108063	1	1	0	1	3
rno-miR-124-3p	MIMAT0000828	LOC6788	678880	XM_001053139	1	1	0	1	3
rno-miR-124-3p	MIMAT0000828	LOC6848	684871	NM_001115043	1	1	0	1	3
rno-miR-124-3p	MIMAT0000828	Trappc10	309678	NM_001173528	1	1	0	1	3
rno-miR-124-3p	MIMAT0000828	Foxl2	367152	XM_003754448	1	1	0	1	3
rno-miR-124-3p	MIMAT0000828	Pcdha8	116781	NM_053942	1	1	0	1	3
rno-miR-124-3p	MIMAT0000828	LOC1003	100359668	XM_002729625	1	1	0	1	3
rno-miR-124-3p	MIMAT0000828	Ndrg2	171114	NM_133583	1	1	0	1	3
rno-miR-124-3p	MIMAT0000828	LOC1003	100365259	XM_002725580	1	1	0	1	3
rno-miR-124-3p	MIMAT0000828	Gng2	80850	NM_001257349	1	1	0	1	3
rno-miR-124-3p	MIMAT0000828	Ubxn7	303878	NM_001107086	1	1	0	1	3
rno-miR-124-3p	MIMAT0000828	Phip	315843	XM_003754471	1	1	0	1	3
rno-miR-124-3p	MIMAT0000828	Tbx19	304935	NM_001107193	1	1	0	1	3
rno-miR-124-3p	MIMAT0000828	Itfg3	360502	NM_001009701	1	1	0	1	3
rno-miR-124-3p	MIMAT0000828	Tspan11	312727	NM_001024262	1	1	0	1	3
rno-miR-124-3p	MIMAT0000828	Ube2v2	287927	NM_183052	1	1	0	1	3
rno-miR-124-3p	MIMAT0000828	Crot	83842	NM_031987	1	1	0	1	3
rno-miR-124-3p	MIMAT0000828	Gnb3	60449	NM_021858	1	1	0	1	3
rno-miR-124-3p	MIMAT0000828	Amot	300289	XM_235733	1	1	0	1	3
rno-miR-124-3p	MIMAT0000828	Itsn2	313934	XM_003750126	1	1	0	1	3
rno-miR-124-3p	MIMAT0000828	Rab7b	501854	NM_001109328	1	1	0	1	3
rno-miR-124-3p	MIMAT0000828	Top3a	303194	XM_002742451	1	1	0	1	3
rno-miR-124-3p	MIMAT0000828	Pgm1	24645	NM_017033	1	1	0	1	3
rno-miR-124-3p	MIMAT0000828	Impact	497198	NM_001012235	1	1	0	1	3
rno-miR-124-3p	MIMAT0000828	Galnt6	100361647	NM_001172063	1	1	0	1	3
rno-miR-124-3p	MIMAT0000828	LOC1009	100910429	XM_003749272	1	1	0	1	3
rno-miR-124-3p	MIMAT0000828	Rbm33	362297	XM_001059091	1	1	0	1	3
rno-miR-124-3p	MIMAT0000828	Sorbs1	686098	XM_001066536	1	1	0	1	3
rno-miR-124-3p	MIMAT0000828	Dlg5	305645	XM_003751424	1	1	0	1	3
rno-miR-124-3p	MIMAT0000828	Tsen54	690308	NM_001109576	1	1	0	1	3

rno-miR-124-3p	MIMAT0000828	Cacna1a	25398	NM_012918	1	1	0	1	3
rno-miR-124-3p	MIMAT0000828	Tmx4	296182	NM_001100529	1	1	0	1	3
rno-miR-124-3p	MIMAT0000828	Lrp1b	311926	NM_001107843	1	1	0	1	3
rno-miR-124-3p	MIMAT0000828	Rcc2	298594	XM_216557	1	1	0	1	3
rno-miR-124-3p	MIMAT0000828	LOC1009	100912493	XM_003754144	1	1	0	1	3
rno-miR-124-3p	MIMAT0000828	Pan3	360760	XM_346914	1	1	0	1	3
rno-miR-124-3p	MIMAT0000828	LOC5676	56764	NM_020089	1	1	0	1	3
rno-miR-124-3p	MIMAT0000828	Shpk	287479	NM_001033682	1	1	0	1	3
rno-miR-124-3p	MIMAT0000828	Pcdh9	306091	NM_001191688	1	1	0	1	3
rno-miR-124-3p	MIMAT0000828	Sh2b3	58838	NM_031621	1	1	0	1	3
rno-miR-124-3p	MIMAT0000828	Hivep3	313557	NM_001107972	1	1	0	1	3
rno-miR-124-3p	MIMAT0000828	Srl	302948	XM_003750771	1	1	0	1	3
rno-miR-124-3p	MIMAT0000828	Fam150b	679566	NM_001109372	1	1	0	1	3
rno-miR-124-3p	MIMAT0000828	Grm1	24414	NM_001114330	1	1	0	1	3
rno-miR-124-3p	MIMAT0000828	Syk	25155	NM_012758	1	1	0	1	3
rno-miR-124-3p	MIMAT0000828	Myadm	369016	NM_183332	1	1	0	1	3
rno-miR-124-3p	MIMAT0000828	Tsc22d1	498545	NM_001109912	1	1	0	1	3
rno-miR-124-3p	MIMAT0000828	C9	117512	NM_057146	1	1	0	1	3
rno-miR-124-3p	MIMAT0000828	LOC1003	100360412	XM_003753013	1	1	0	1	3
rno-miR-124-3p	MIMAT0000828	LOC1009	100909504	XM_003752259	1	1	0	1	3
rno-miR-124-3p	MIMAT0000828	Tubgcp4	362203	XM_003753765	1	1	0	1	3
rno-miR-124-3p	MIMAT0000828	Rarg	685072	NM_001135250	1	1	0	1	3
rno-miR-124-3p	MIMAT0000828	Lin9	689523	XM_001067807	1	1	0	1	3
rno-miR-124-3p	MIMAT0000828	Cd38	25668	NM_013127	1	1	0	1	3
rno-miR-124-3p	MIMAT0000828	Asxl1	311553	XM_002729218	1	1	0	1	3
rno-miR-124-3p	MIMAT0000828	Smim8	297971	NM_001201374	1	1	0	1	3
rno-miR-124-3p	MIMAT0000828	Ighmbp2	29532	NM_031586	1	1	0	1	3
rno-miR-124-3p	MIMAT0000828	Impad1	312952	NM_001008772	1	1	0	1	3
rno-miR-124-3p	MIMAT0000828	Tmprss15	288291	NM_001105895	1	1	0	1	3
rno-miR-124-3p	MIMAT0000828	Wnk1	116477	NM_001199095	1	1	0	1	3
rno-miR-124-3p	MIMAT0000828	Rab10	50993	NM_017359	1	1	0	1	3
rno-miR-124-3p	MIMAT0000828	Nfx1	313166	NM_001024784	1	1	0	1	3
rno-miR-124-3p	MIMAT0000828	Wdr81	303312	NM_001134360	1	1	0	1	3
rno-miR-124-3p	MIMAT0000828	Tmem234	682404	XM_003750040	1	1	0	1	3
rno-miR-124-3p	MIMAT0000828	Sord	24788	NM_017052	1	1	0	1	3
rno-miR-124-3p	MIMAT0000828	Otor	366206	NM_001108960	1	1	0	1	3
rno-miR-124-3p	MIMAT0000828	Cd2bp2	293505	NM_001106297	1	1	0	1	3
rno-miR-124-3p	MIMAT0000828	Sphk1	170897	NM_133386	1	1	0	1	3
rno-miR-124-3p	MIMAT0000828	Fam105b	100362554	XM_002729016	1	1	0	1	3
rno-miR-124-3p	MIMAT0000828	Lmbrd1	246046	NM_139189	1	1	0	1	3
rno-miR-124-3p	MIMAT0000828	Ube4a	315608	NM_207610	1	1	0	1	3
rno-miR-124-3p	MIMAT0000828	Tbl2	686610	XM_003751162	1	1	0	1	3
rno-miR-124-3p	MIMAT0000828	Fmn1	296512	XM_002726209	1	1	0	1	3
rno-miR-124-3p	MIMAT0000828	Pdyn	29190	NM_019374	1	1	0	1	3
rno-miR-124-3p	MIMAT0000828	Cspp1	362472	NM_001191864	1	1	0	1	3
rno-miR-124-3p	MIMAT0000828	Hoxa5	79241	XM_003749749	1	1	0	1	3
rno-miR-124-3p	MIMAT0000828	Ube2o	303689	XM_001081723	1	1	0	1	3
rno-miR-124-3p	MIMAT0000828	Cilp	315761	NM_001108161	1	1	0	1	3
rno-miR-124-3p	MIMAT0000828	Bloc1s6	317630	NM_001025714	1	1	0	1	3
rno-miR-124-3p	MIMAT0000828	Morn3	687615	XM_001079404	1	1	0	1	3
rno-miR-124-3p	MIMAT0000828	Lhfpl5	294303	NM_001013906	1	1	0	1	3
rno-miR-124-3p	MIMAT0000828	Sepw1	25545	NM_013027	1	1	0	1	3
rno-miR-124-3p	MIMAT0000828	Aass	296925	NM_001100963	1	1	0	1	3
rno-miR-124-3p	MIMAT0000828	Lrig1	312574	XM_001076882	1	1	0	1	3
rno-miR-124-3p	MIMAT0000828	Frmd4b	252858	XM_003749805	1	1	0	1	3
rno-miR-124-3p	MIMAT0000828	LOC1009	100911615	XM_003749015	1	1	0	1	3
rno-miR-124-3p	MIMAT0000828	Smco4	363020	NM_001134584	1	1	0	1	3
rno-miR-124-3p	MIMAT0000828	Slfn3	114247	NM_053687	1	1	0	1	3
rno-miR-124-3p	MIMAT0000828	Ercc6	306274	NM_001107296	1	1	0	1	3
rno-miR-124-3p	MIMAT0000828	Gria4	29629	NM_001113184	1	1	0	1	3
rno-miR-124-3p	MIMAT0000828	Grhl2	299979	NM_001134527	1	1	0	1	3
rno-miR-124-3p	MIMAT0000828	Klhl21	313743	NM_001107996	1	1	0	1	3
rno-miR-124-3p	MIMAT0000828	Dyrk2	314862	NM_001108100	1	1	0	1	3
rno-miR-124-3p	MIMAT0000828	Xpo4	290280	NM_001106042	1	1	0	1	3
rno-miR-124-3p	MIMAT0000828	Ctu1	292847	NM_001106251	1	1	0	1	3
rno-miR-124-3p	MIMAT0000828	Bckdha	25244	NM_012782	1	1	0	1	3
rno-miR-124-3p	MIMAT0000828	Pcdha11	394223	NM_199486	1	1	0	1	3
rno-miR-124-3p	MIMAT0000828	Snhg11	362256	NM_001258011	1	1	0	1	3
rno-miR-124-3p	MIMAT0000828	Fam83f	315145	NM_001130502	1	1	0	1	3

rno-miR-124-3p	MIMAT0000828	RGD1560	304297	XM_003751132	1	1	0	1	3
rno-miR-124-3p	MIMAT0000828	LOC6857	685722	NM_001109479	1	1	0	1	3
rno-miR-124-3p	MIMAT0000828	Cd3d	25710	NM_013169	1	1	0	1	3
rno-miR-124-3p	MIMAT0000828	Rffl	282844	NM_001004068	1	1	0	1	3
rno-miR-124-3p	MIMAT0000828	Vps37b	288659	NM_001105928	1	1	0	1	3
rno-miR-124-3p	MIMAT0000828	Shc1	85385	NM_053517	1	1	0	1	3
rno-miR-124-3p	MIMAT0000828	Atg12	361321	NM_001038495	1	1	0	1	3
rno-miR-124-3p	MIMAT0000828	Ptprd	313278	XM_001067936	1	1	0	1	3
rno-miR-124-3p	MIMAT0000828	Dcaf5	314273	NM_001100718	1	1	0	1	3
rno-miR-124-3p	MIMAT0000828	Fam134b	619558	NM_001034912	1	1	0	1	3
rno-miR-124-3p	MIMAT0000828	Osbpl7	303497	NM_001107044	1	1	0	1	3
rno-miR-124-3p	MIMAT0000828	LOC6836	683674	XM_003751128	1	1	0	1	3
rno-miR-124-3p	MIMAT0000828	Dpy19l3	308519	NM_001135835	1	1	0	1	3
rno-miR-124-3p	MIMAT0000828	Maff	366960	NM_001130573	1	1	0	1	3
rno-miR-124-3p	MIMAT0000828	Aff2	293922	XM_001054673	1	1	0	1	3
rno-miR-124-3p	MIMAT0000828	LOC4981	498154	NM_001025033	1	1	0	1	3
rno-miR-124-3p	MIMAT0000828	LOC1003	100363361	XM_002729081	1	1	0	1	3
rno-miR-124-3p	MIMAT0000828	Ndufaf3	56769	NM_001033971	1	1	0	1	3
rno-miR-124-3p	MIMAT0000828	Eif2s3x	299027	NM_001100542	1	1	0	1	3
rno-miR-124-3p	MIMAT0000828	Fam174a	301634	NM_001012350	1	1	0	1	3
rno-miR-124-3p	MIMAT0000828	LOC6848	684892	XM_003752077	1	1	0	1	3
rno-miR-124-3p	MIMAT0000828	Lrrc52	289199	NM_001077434	1	1	0	1	3
rno-miR-124-3p	MIMAT0000828	Mgat4a	367252	NM_001012225	1	1	0	1	3
rno-miR-124-3p	MIMAT0000828	Phf6	100359714	XM_002727289	1	1	0	1	3
rno-miR-124-3p	MIMAT0000828	Rph3al	171123	NM_133591	1	1	0	1	3
rno-miR-124-3p	MIMAT0000828	Ctdsp2	100365370	XM_002726958	1	1	0	1	3
rno-miR-124-3p	MIMAT0000828	Gtf3c5	362095	NM_001079941	1	1	0	1	3
rno-miR-124-3p	MIMAT0000828	Gng2	80850	NM_031754	1	1	0	1	3
rno-miR-124-3p	MIMAT0000828	Osbpl11	303888	NM_001107090	1	1	0	1	3
rno-miR-124-3p	MIMAT0000828	Nprl3	360505	NM_001034936	1	1	0	1	3
rno-miR-124-3p	MIMAT0000828	Nap1l5	688843	NM_001044293	1	1	0	1	3
rno-miR-124-3p	MIMAT0000828	Hspa12b	311427	NM_001107778	1	1	0	1	3
rno-miR-124-3p	MIMAT0000828	Edem1	297504	XM_001075943	1	1	0	1	3
rno-miR-124-3p	MIMAT0000828	Fibin	499856	NM_001025042	1	1	0	1	3
rno-miR-124-3p	MIMAT0000828	Gpx6	259233	NM_147165	1	1	0	1	3
rno-miR-124-3p	MIMAT0000828	Ralbp1	84014	NM_032067	1	1	0	1	3
rno-miR-124-3p	MIMAT0000828	Ppp3r2	29749	NM_021701	1	1	0	1	3
rno-miR-124-3p	MIMAT0000828	Itsn2	313934	XM_003754159	1	1	0	1	3
rno-miR-124-3p	MIMAT0000828	Gatad2a	290669	NM_001013881	1	1	0	1	3
rno-miR-124-3p	MIMAT0000828	Zfp592	293038	NM_001106272	1	1	0	1	3
rno-miR-124-3p	MIMAT0000828	Lipe	25330	NM_012859	1	1	0	1	3
rno-miR-124-3p	MIMAT0000828	Plb1	192259	NM_138898	1	1	0	1	3
rno-miR-124-3p	MIMAT0000828	Fam133b	362320	NM_001024799	1	1	0	1	3
rno-miR-124-3p	MIMAT0000828	Aldh3a2	65183	NM_031731	1	1	0	1	3
rno-miR-124-3p	MIMAT0000828	Rbm19	304512	XM_003751194	1	1	0	1	3
rno-miR-124-3p	MIMAT0000828	Trip12	316575	NM_001031659	1	1	0	1	3
rno-miR-124-3p	MIMAT0000828	Sorbs1	686098	XM_002725799	1	1	0	1	3
rno-miR-124-3p	MIMAT0000828	Dlg5	305645	XM_003752781	1	1	0	1	3
rno-miR-124-3p	MIMAT0000828	Snx5	296199	NM_001106518	1	1	0	1	3
rno-miR-124-3p	MIMAT0000828	RGD1563	499114	NM_001110491	1	1	0	1	3
rno-miR-124-3p	MIMAT0000828	Ccdc71l	500640	XM_002726691	1	1	0	1	3
rno-miR-124-3p	MIMAT0000828	Timd2	287222	NM_001013855	1	1	0	1	3
rno-miR-124-3p	MIMAT0000828	Zdhhc23	363783	NM_213627	1	1	0	1	3
rno-miR-124-3p	MIMAT0000828	Smpd3	94338	NM_053605	1	1	0	1	3
rno-miR-124-3p	MIMAT0000828	Mtmr6	305935	NM_001107268	1	1	0	1	3
rno-miR-124-3p	MIMAT0000828	Ccz1	360768	NM_001014126	1	1	0	1	3
rno-miR-124-3p	MIMAT0000828	RGD1308	307249	NM_001107374	1	1	0	1	3
rno-miR-124-3p	MIMAT0000828	Nudt7	361413	NM_001108450	1	1	0	1	3
rno-miR-124-3p	MIMAT0000828	Calml5	364774	XM_001062982	1	1	0	1	3
rno-miR-124-3p	MIMAT0000828	Nkx2-5	114109	NM_053651	1	1	0	1	3
rno-miR-124-3p	MIMAT0000828	Cln5	306128	NM_001191689	1	1	0	1	3
rno-miR-124-3p	MIMAT0000828	Fut2	58924	NM_031635	1	1	0	1	3
rno-miR-124-3p	MIMAT0000828	Mbrl	299608	NM_001100886	1	1	0	1	3
rno-miR-124-3p	MIMAT0000828	Snip1	313588	NM_001014069	1	1	0	1	3
rno-miR-124-3p	MIMAT0000828	Srl	302948	XM_003752294	1	1	0	1	3
rno-miR-124-3p	MIMAT0000828	Tmem178	679651	NM_001195277	1	1	0	1	3
rno-miR-124-3p	MIMAT0000828	Grm1	24414	NM_017011	1	1	0	1	3
rno-miR-124-3p	MIMAT0000828	Stx11	292483	NM_001025638	1	1	0	1	3
rno-miR-124-3p	MIMAT0000828	Tsc22d1	498545	NM_013043	1	1	0	1	3

rno-miR-124-3p	MIMAT0000828	Keap1	117519	NM_057152	1	1	0	1	3
rno-miR-124-3p	MIMAT0000828	Slc12a5	171373	NM_134363	1	1	0	1	3
rno-miR-124-3p	MIMAT0000828	Cpox	304024	NM_001037095	1	1	0	1	3
rno-miR-124-3p	MIMAT0000828	Lin9	689523	XM_003752692	1	1	0	1	3
rno-miR-124-3p	MIMAT0000828	Cep250	311573	XM_002726274	0	1	1	1	3
rno-miR-124-3p	MIMAT0000828	Smim8	297971	NM_001201375	1	1	0	1	3
rno-miR-124-3p	MIMAT0000828	Sfn	313017	XM_001065560	1	1	0	1	3
rno-miR-124-3p	MIMAT0000828	Zfp12	288486	XM_001072483	1	1	0	1	3
rno-miR-124-3p	MIMAT0000828	Xpo5	363194	NM_001108789	1	1	0	1	3
rno-miR-124-3p	MIMAT0000828	Slc24a2	84550	NM_031743	1	1	0	1	3
rno-miR-124-3p	MIMAT0000828	Wnk1	116477	NM_053794	1	1	0	1	3
rno-miR-124-3p	MIMAT0000828	Snai3	307919	NM_001107439	1	1	0	1	3
rno-miR-124-3p	MIMAT0000828	Nol6	313167	XM_003749924	1	1	0	1	3
rno-miR-124-3p	MIMAT0000828	Shisa5	301013	NM_001006989	1	1	0	1	3
rno-miR-124-3p	MIMAT0000828	Atad5	303348	XM_001080963	1	1	0	1	3
rno-miR-124-3p	MIMAT0000828	Sp1	24790	NM_012655	1	1	0	1	3
rno-miR-124-3p	MIMAT0000828	Epc1	100362678	XM_002725273	1	1	0	1	3
rno-miR-124-3p	MIMAT0000828	Aifm2	361843	NM_001139483	1	1	0	1	3
rno-miR-124-3p	MIMAT0000828	Calcoco1	246047	NM_139190	1	1	0	1	3
rno-miR-124-3p	MIMAT0000828	Mepe	79110	NM_024142	1	1	0	1	3
rno-miR-124-3p	MIMAT0000828	Scn4b	315611	NM_001008880	1	1	0	1	3
rno-miR-124-3p	MIMAT0000828	Narg2	691379	NM_001191108	1	1	0	1	3
rno-miR-124-3p	MIMAT0000828	Fmn1	296512	XM_231006	1	1	0	1	3
rno-miR-124-3p	MIMAT0000828	Casq2	29209	NM_017131	1	1	0	1	3
rno-miR-124-3p	MIMAT0000828	LOC1003	100363361	XM_002742445	1	1	0	1	3
rno-miR-124-3p	MIMAT0000828	Them5	361993	NM_001108558	1	1	0	1	3
rno-miR-124-3p	MIMAT0000828	Hoxa5	79241	XM_003753897	1	1	0	1	3
rno-miR-124-3p	MIMAT0000828	Ube2o	303689	XM_221132	1	1	0	1	3
rno-miR-124-3p	MIMAT0000828	Fam81a	315789	NM_001108163	1	1	0	1	3
rno-miR-124-3p	MIMAT0000828	Elk4	304786	NM_001107173	1	1	0	1	3
rno-miR-124-3p	MIMAT0000828	Nsmaf	353233	NM_181389	1	1	0	1	3
rno-miR-124-3p	MIMAT0000828	Morn3	687615	XM_003751184	1	1	0	1	3
rno-miR-124-3p	MIMAT0000828	Lrig1	312574	XM_003749802	1	1	0	1	3
rno-miR-124-3p	MIMAT0000828	Frmd4b	252858	XM_003753925	1	1	0	1	3
rno-miR-124-3p	MIMAT0000828	LOC1009	100911730	XM_003749017	1	1	0	1	3
rno-miR-124-3p	MIMAT0000828	Slc44a2	363024	NM_001134715	1	1	0	1	3
rno-miR-124-3p	MIMAT0000828	Nanos2	365213	NM_001108908	1	1	0	1	3
rno-miR-124-3p	MIMAT0000828	Pde6h	114248	NM_053688	1	1	0	1	3
rno-miR-124-3p	MIMAT0000828	Syt15	306285	NM_181632	1	1	0	1	3
rno-miR-124-3p	MIMAT0000828	Tbc1d1	360937	XM_001071842	1	1	0	1	3
rno-miR-124-3p	MIMAT0000828	Gria4	29629	NM_017263	1	1	0	1	3
rno-miR-124-3p	MIMAT0000828	Rapgef3	59326	NM_021690	1	1	0	1	3
rno-miR-124-3p	MIMAT0000828	Ski	313757	XM_001077382	1	1	0	1	3
rno-miR-124-3p	MIMAT0000828	Efhd1	501181	NM_001109310	1	1	0	1	3
rno-miR-124-3p	MIMAT0000828	Adam19	303068	NM_001160228	1	1	0	1	3
rno-miR-124-3p	MIMAT0000828	Dok2	290361	NM_001106048	1	1	0	1	3
rno-miR-124-3p	MIMAT0000828	Emc10	292878	NM_001004221	1	1	0	1	3
rno-miR-124-3p	MIMAT0000828	Bdkrb2	25245	NM_173100	1	1	0	1	3
rno-miR-124-3p	MIMAT0000828	Sema5a	310207	NM_001107659	1	1	0	1	3
rno-miR-124-3p	MIMAT0000828	aicda	399679	NM_001100779	1	1	0	1	3
rno-miR-124-3p	MIMAT0000828	Scube1	315174	NM_001134884	1	1	0	1	3
rno-miR-124-3p	MIMAT0000828	Gmfb	81661	NM_031032	1	1	0	1	3
rno-miR-124-3p	MIMAT0000828	RGD1560	304297	XM_003752567	1	1	0	1	3
rno-miR-124-3p	MIMAT0000828	Creg2	316353	XM_003750685	1	1	0	1	3
rno-miR-124-3p	MIMAT0000828	Pik3ip1	305472	NM_001017453	1	1	0	1	3
rno-miR-124-3p	MIMAT0000828	Wdr4	690032	NM_001135667	1	1	0	1	3
rno-miR-124-3p	MIMAT0000828	Ntf4	25730	NM_013184	1	1	0	1	3
rno-miR-124-3p	MIMAT0000828	RGD1559	298384	NM_001034132	1	1	0	1	3
rno-miR-124-3p	MIMAT0000828	RGD1308	362790	XM_001072750	1	1	0	1	3
rno-miR-124-3p	MIMAT0000828	Unc119b	288702	NM_001105934	1	1	0	1	3
rno-miR-124-3p	MIMAT0000828	Rhoq	85428	NM_053522	1	1	0	1	3
rno-miR-124-3p	MIMAT0000828	Lgals8	116641	NM_053862	1	1	0	1	3
rno-miR-124-3p	MIMAT0000828	Sema6a	361324	XM_003751782	1	1	0	1	3
rno-miR-124-3p	MIMAT0000828	Syde2	308021	XM_003749414	1	1	0	1	3
rno-miR-124-3p	MIMAT0000828	Ptprd	313278	XM_003749965	1	1	0	1	3
rno-miR-124-3p	MIMAT0000828	Flvcr2	314323	NM_199109	1	1	0	1	3
rno-miR-124-3p	MIMAT0000828	Fam104a	619573	NM_001034958	1	1	0	1	3
rno-miR-124-3p	MIMAT0000828	Tns4	303517	NM_001024881	1	1	0	1	3
rno-miR-124-3p	MIMAT0000828	LOC6837	683722	NM_001101008	1	1	0	1	3

rno-miR-124-3p	MIMAT0000828	Asgr1	24210	NM_012503	1	1	0	1	3
rno-miR-124-3p	MIMAT0000828	Aff2	293922	XM_219832	1	1	0	1	3
rno-miR-124-3p	MIMAT0000828	Serpinb3a	498209	XM_001056687	1	1	0	1	3
rno-miR-124-3p	MIMAT0000828	Ndufaf3	56769	NM_020080	1	1	0	1	3
rno-miR-124-3p	MIMAT0000828	Dnajc6	313409	NM_001107949	1	1	0	1	3
rno-miR-124-3p	MIMAT0000828	Calu	64366	NM_001033898	1	1	0	1	3
rno-miR-124-3p	MIMAT0000828	Sbno2	314619	NM_001108068	1	1	0	1	3
rno-miR-124-3p	MIMAT0000828	LOC6788	678893	XM_001053842	1	1	0	1	3
rno-miR-124-3p	MIMAT0000828	LOC6848	684892	XM_003754613	1	1	0	1	3
rno-miR-124-3p	MIMAT0000828	Nhlh1	289230	NM_001105970	1	1	0	1	3
rno-miR-124-3p	MIMAT0000828	Ednra	24326	NM_012550	1	1	0	1	3
rno-miR-124-3p	MIMAT0000828	Cmtr2	292016	NM_001106186	1	1	0	1	3
rno-miR-124-3p	MIMAT0000828	Mgat4a	367252	NM_001160155	1	1	0	1	3
rno-miR-124-3p	MIMAT0000828	Phf6	100359714	XM_003752156	1	1	0	1	3
rno-miR-124-3p	MIMAT0000828	Ctdsp2	100365370	XM_003750330	1	1	0	1	3
rno-miR-124-3p	MIMAT0000828	Aif1l	362107	NM_001108578	1	1	0	1	3
rno-miR-124-3p	MIMAT0000828	Adamtsl1	362539	XM_002726547	1	1	0	1	3
rno-miR-124-3p	MIMAT0000828	Pim3	64534	NM_022602	1	1	0	1	3
rno-miR-124-3p	MIMAT0000828	Tmem206	305070	NM_001007679	1	1	0	1	3
rno-miR-124-3p	MIMAT0000828	Cmya5	688915	XM_001068814	1	1	0	1	3
rno-miR-124-3p	MIMAT0000828	Il7r	294797	NM_001106418	1	1	0	1	3
rno-miR-124-3p	MIMAT0000828	Crem	25620	NM_001110860	1	1	0	1	3
rno-miR-124-3p	MIMAT0000828	Rassf2	311437	NM_001037096	1	1	0	1	3
rno-miR-124-3p	MIMAT0000828	Edem1	297504	XM_238366	1	1	0	1	3
rno-miR-124-3p	MIMAT0000828	Ptbp1	29497	NM_022516	1	1	0	1	3
rno-miR-124-3p	MIMAT0000828	Atf7ip	312800	XM_003749858	1	1	0	1	3
rno-miR-124-3p	MIMAT0000828	Gabpb1	499883	NM_001039036	1	1	0	1	3
rno-miR-124-3p	MIMAT0000828	LOC1009	100911776	XM_003748922	1	1	0	1	3
rno-miR-124-3p	MIMAT0000828	Poglut1	288091	NM_001100652	1	1	0	1	3
rno-miR-124-3p	MIMAT0000828	Mrap2	363112	NM_001108774	1	1	0	1	3
rno-miR-124-3p	MIMAT0000828	Nek3	306576	XM_001065115	1	1	0	1	3
rno-miR-124-3p	MIMAT0000828	Tmem260	361030	NM_001170475	1	1	0	1	3
rno-miR-124-3p	MIMAT0000828	Gpr114	307645	NM_001107410	1	1	0	1	3
rno-miR-124-3p	MIMAT0000828	Med17	300367	NM_001106801	1	1	0	1	3
rno-miR-124-3p	MIMAT0000828	Itsn2	313934	XM_003754160	1	1	0	1	3
rno-miR-124-3p	MIMAT0000828	RGD1564	502093	XM_003751612	1	1	0	1	3
rno-miR-124-3p	MIMAT0000828	Zfp287	303212	NM_001107008	1	1	0	1	3
rno-miR-124-3p	MIMAT0000828	Trappc11	290746	NM_001169115	1	1	0	1	3
rno-miR-124-3p	MIMAT0000828	Plxna3	309280	NM_001107581	1	1	0	1	3
rno-miR-124-3p	MIMAT0000828	Mesp2	293046	NM_001106273	1	1	0	1	3
rno-miR-124-3p	MIMAT0000828	Prr3	361788	NM_212544	1	1	0	1	3
rno-miR-124-3p	MIMAT0000828	Foxe1	192274	NM_138909	1	1	0	1	3
rno-miR-124-3p	MIMAT0000828	LOC1009	100910454	XM_003750751	1	1	0	1	3
rno-miR-124-3p	MIMAT0000828	Rbm19	304512	XM_003752610	1	1	0	1	3
rno-miR-124-3p	MIMAT0000828	Sorbs1	686098	XM_003749110	1	1	0	1	3
rno-miR-124-3p	MIMAT0000828	Dlg5	305645	XM_003752782	1	1	0	1	3
rno-miR-124-3p	MIMAT0000828	Slc22a15	310732	NM_001107707	1	1	0	1	3
rno-miR-124-3p	MIMAT0000828	Ovol2	296201	NM_001106519	1	1	0	1	3
rno-miR-124-3p	MIMAT0000828	Ccdc71l	500640	XM_003750141	1	1	0	1	3
rno-miR-124-3p	MIMAT0000828	Zfand2a	360772	NM_001008363	1	1	0	1	3
rno-miR-124-3p	MIMAT0000828	Rnf165	307251	NM_001164505	1	1	0	1	3
rno-miR-124-3p	MIMAT0000828	Galnt4	500826	NM_001025053	1	1	0	1	3
rno-miR-124-3p	MIMAT0000828	LOC1009	100911154	XM_003749361	1	1	0	1	3
rno-miR-124-3p	MIMAT0000828	Serpinf2	287527	NM_001011892	1	1	0	1	3
rno-miR-124-3p	MIMAT0000828	Tmem184	362959	NM_001173370	1	1	0	1	3
rno-miR-124-3p	MIMAT0000828	Calml5	364774	XM_344627	1	1	0	1	3
rno-miR-124-3p	MIMAT0000828	Gng10	114119	NM_053660	1	1	0	1	3
rno-miR-124-3p	MIMAT0000828	Rras	361568	NM_001108481	1	1	0	1	3
rno-miR-124-3p	MIMAT0000828	Ptafr	58949	NM_053321	1	1	0	1	3
rno-miR-124-3p	MIMAT0000828	Ago1	313594	NM_001191765	1	1	0	1	3
rno-miR-124-3p	MIMAT0000828	Abca3	302973	XM_001054650	1	1	0	1	3
rno-miR-124-3p	MIMAT0000828	Dram1	679937	NM_001173427	1	1	0	1	3
rno-miR-124-3p	MIMAT0000828	Gusb	24434	NM_017015	1	1	0	1	3
rno-miR-124-3p	MIMAT0000828	Zfp418	292548	NM_001191620	1	1	0	1	3
rno-miR-124-3p	MIMAT0000828	Efnb1	25186	NM_017089	1	1	0	1	3
rno-miR-124-3p	MIMAT0000828	Prkag2	373545	NM_184051	1	1	0	1	3
rno-miR-124-3p	MIMAT0000828	Fam57a	100360533	XM_003752344	1	1	0	1	3
rno-miR-124-3p	MIMAT0000828	LOC1009	100909630	XM_003751925	1	1	0	1	3
rno-miR-124-3p	MIMAT0000828	Gpatch8	685233	XM_001062937	1	1	0	1	3

rno-miR-124-3p	MIMAT0000828	Cep135	305288	XM_001076228	1	1	0	1	3
rno-miR-124-3p	MIMAT0000828	Ankrd13b	360575	XM_001080794	1	1	0	1	3
rno-miR-124-3p	MIMAT0000828	Hist2h2be	295274	XM_001061909	1	1	0	1	3
rno-miR-124-3p	MIMAT0000828	Nr3c2	25672	NM_013131	1	1	0	1	3
rno-miR-124-3p	MIMAT0000828	Cep250	311573	XM_230784	0	1	1	1	3
rno-miR-124-3p	MIMAT0000828	Pdx1	29535	NM_022852	1	1	0	1	3
rno-miR-124-3p	MIMAT0000828	Sfn	313017	XM_003750048	1	1	0	1	3
rno-miR-124-3p	MIMAT0000828	Ttc26	500086	NM_001025045	1	1	0	1	3
rno-miR-124-3p	MIMAT0000828	Mipol1	100911970	XM_003750162	1	1	0	1	3
rno-miR-124-3p	MIMAT0000828	Zfp12	288486	XM_003751129	1	1	0	1	3
rno-miR-124-3p	MIMAT0000828	Raph1	363239	NM_001108798	1	1	0	1	3
rno-miR-124-3p	MIMAT0000828	Cln8	306619	NM_001007686	1	1	0	1	3
rno-miR-124-3p	MIMAT0000828	Cdc14b	361195	NM_001108404	1	1	0	1	3
rno-miR-124-3p	MIMAT0000828	Nol6	313167	XM_003754006	1	1	0	1	3
rno-miR-124-3p	MIMAT0000828	Tmem194	503257	NM_001134642	1	1	0	1	3
rno-miR-124-3p	MIMAT0000828	Atad5	303348	XM_003750868	1	1	0	1	3
rno-miR-124-3p	MIMAT0000828	Zim1	308322	NM_001107473	1	1	0	1	3
rno-miR-124-3p	MIMAT0000828	Tgfa	24827	NM_012671	1	1	0	1	3
rno-miR-124-3p	MIMAT0000828	Rbm38	366262	NM_001108965	1	1	0	1	3
rno-miR-124-3p	MIMAT0000828	Ate1	293526	NM_001106300	1	1	0	1	3
rno-miR-124-3p	MIMAT0000828	Epc1	100362678	XM_002728490	1	1	0	1	3
rno-miR-124-3p	MIMAT0000828	Scd1	246074	NM_139192	1	1	0	1	3
rno-miR-124-3p	MIMAT0000828	LOC1009	100910732	XM_003751066	1	1	0	1	3
rno-miR-124-3p	MIMAT0000828	RGD1563	315652	XM_001071358	1	1	0	1	3
rno-miR-124-3p	MIMAT0000828	Cyr61	83476	NM_031327	1	1	0	1	3
rno-miR-124-3p	MIMAT0000828	Chst14	691394	NM_001109639	1	1	0	1	3
rno-miR-124-3p	MIMAT0000828	Adh5	100145871	NM_001126120	1	1	0	1	3
rno-miR-124-3p	MIMAT0000828	Thap2	688019	XM_001080899	1	1	0	1	3
rno-miR-124-3p	MIMAT0000828	LOC6919	691920	XM_003749562	1	1	0	1	3
rno-miR-124-3p	MIMAT0000828	Abcc9	25560	NM_013040	1	1	0	1	3
rno-miR-124-3p	MIMAT0000828	Dll4	311332	NM_001107760	1	1	0	1	3
rno-miR-124-3p	MIMAT0000828	Lrig1	312574	XM_003753923	1	1	0	1	3
rno-miR-124-3p	MIMAT0000828	RGD1560	499724	NM_001134616	1	1	0	1	3
rno-miR-124-3p	MIMAT0000828	Frmd4b	252858	XM_003753926	1	1	0	1	3
rno-miR-124-3p	MIMAT0000828	Dlx3	287638	NM_001105832	1	1	0	1	3
rno-miR-124-3p	MIMAT0000828	Msx3	114504	NM_053712	1	1	0	1	3
rno-miR-124-3p	MIMAT0000828	Mmrn2	306288	XM_003751550	1	1	0	1	3
rno-miR-124-3p	MIMAT0000828	Tbc1d1	360937	XM_003752723	1	1	0	1	3
rno-miR-124-3p	MIMAT0000828	Hsd3b6	29632	NM_017265	1	1	0	1	3
rno-miR-124-3p	MIMAT0000828	Ski	313757	XM_233731	1	1	0	1	3
rno-miR-124-3p	MIMAT0000828	Pkhd1l1	314917	NM_001034931	1	1	0	1	3
rno-miR-124-3p	MIMAT0000828	LOC6806	680656	XM_001058198	1	1	0	1	3
rno-miR-124-3p	MIMAT0000828	Akt1s1	292887	NM_001106259	1	1	0	1	3
rno-miR-124-3p	MIMAT0000828	Usp13	310306	NM_001107665	1	1	0	1	3
rno-miR-124-3p	MIMAT0000828	Mid1ip1	404280	NM_206950	1	1	0	1	3
rno-miR-124-3p	MIMAT0000828	Napa	140673	NM_080585	1	1	0	1	3
rno-miR-124-3p	MIMAT0000828	Kdm2a	361700	NM_001108515	1	1	0	1	3
rno-miR-124-3p	MIMAT0000828	Akr1c14	191574	NM_138547	1	1	0	1	3
rno-miR-124-3p	MIMAT0000828	Srsf6	362264	NM_001014185	1	1	0	1	3
rno-miR-124-3p	MIMAT0000828	Mmd2	304301	NM_001037217	1	1	0	1	3
rno-miR-124-3p	MIMAT0000828	Creg2	316353	XM_003754515	1	1	0	1	3
rno-miR-124-3p	MIMAT0000828	LOC6857	685787	XM_003751854	1	1	0	1	3
rno-miR-124-3p	MIMAT0000828	Rnf168	690043	NM_001127597	1	1	0	1	3
rno-miR-124-3p	MIMAT0000828	Lamc3	311862	NM_001107830	1	1	0	1	3
rno-miR-124-3p	MIMAT0000828	RGD1559	498967	NM_001109134	1	1	0	1	3
rno-miR-124-3p	MIMAT0000828	Cmpk1	298410	NM_001025655	1	1	0	1	3
rno-miR-124-3p	MIMAT0000828	RGD1308	362790	XM_003750239	1	1	0	1	3
rno-miR-124-3p	MIMAT0000828	Klf15	85497	NM_053536	1	1	0	1	3
rno-miR-124-3p	MIMAT0000828	Lgals8	116641	XM_003753009	1	1	0	1	3
rno-miR-124-3p	MIMAT0000828	Sema6a	361324	XM_003753046	1	1	0	1	3
rno-miR-124-3p	MIMAT0000828	Syde2	308021	XM_003753666	1	1	0	1	3
rno-miR-124-3p	MIMAT0000828	Ptprd	313278	XM_003754043	1	1	0	1	3
rno-miR-124-3p	MIMAT0000828	Cnnm3	301345	NM_001106901	1	1	0	1	3
rno-miR-124-3p	MIMAT0000828	RGD1309	314330	NM_001108044	1	1	0	1	3
rno-miR-124-3p	MIMAT0000828	LOC6195	619574	NM_001034959	1	1	0	1	3
rno-miR-124-3p	MIMAT0000828	Lrrc37a	303556	XM_001081536	1	1	0	1	3
rno-miR-124-3p	MIMAT0000828	Serpinb3a	498209	XM_573426	1	1	0	1	3
rno-miR-124-3p	MIMAT0000828	Upf3b	313449	NM_001135873	1	1	0	1	3
rno-miR-124-3p	MIMAT0000828	Calu	64366	NM_022535	1	1	0	1	3

rno-miR-124-3p	MIMAT0000828	LOC6788	678893	XM_003750190	1	1	0	1	3
rno-miR-124-3p	MIMAT0000828	Pex19	289233	NM_001107375	1	1	0	1	3
rno-miR-124-3p	MIMAT0000828	Mrvi1	308899	NM_001105210	1	1	0	1	3
rno-miR-124-3p	MIMAT0000828	Urb2	292087	NM_001135708	1	1	0	1	3
rno-miR-124-3p	MIMAT0000828	Col6a3	367313	XM_003750732	1	1	0	1	3
rno-miR-124-3p	MIMAT0000828	Sec14l4	498399	NM_001109090	1	1	0	1	3
rno-miR-124-3p	MIMAT0000828	Kcne2	171138	NM_133603	1	1	0	1	3
rno-miR-124-3p	MIMAT0000828	LOC1003	100365752	XM_003749575	1	1	0	1	3
rno-miR-124-3p	MIMAT0000828	Parp14	303903	NM_001191659	1	1	0	1	3
rno-miR-124-3p	MIMAT0000828	Cmya5	688915	XM_002725910	1	1	0	1	3
rno-miR-124-3p	MIMAT0000828	Crem	25620	NM_017334	1	1	0	1	3
rno-miR-124-3p	MIMAT0000828	Trmt6	311441	NM_001107779	1	1	0	1	3
rno-miR-124-3p	MIMAT0000828	Atf7ip	312800	XM_003753954	1	1	0	1	3
rno-miR-124-3p	MIMAT0000828	LOC4998	499886	NM_001024312	1	1	0	1	3
rno-miR-124-3p	MIMAT0000828	Slc9a9	363115	XM_002729941	1	1	0	1	3
rno-miR-124-3p	MIMAT0000828	Sh3kbp1	84357	NM_053360	1	1	0	1	3
rno-miR-124-3p	MIMAT0000828	Nek3	306576	XM_224971	1	1	0	1	3
rno-miR-124-3p	MIMAT0000828	Adamts15	300474	NM_001106810	1	1	0	1	3
rno-miR-124-3p	MIMAT0000828	Itsn2	313934	XM_003754161	1	1	0	1	3
rno-miR-124-3p	MIMAT0000828	Trim16	303214	NM_001135033	1	1	0	1	3
rno-miR-124-3p	MIMAT0000828	Pip5k1a	365865	NM_001042621	1	1	0	1	3
rno-miR-124-3p	MIMAT0000828	Cpeb1	293056	NM_001106276	1	1	0	1	3
rno-miR-124-3p	MIMAT0000828	Bhlha15	25334	NM_012863	1	1	0	1	3
rno-miR-124-3p	MIMAT0000828	Nrcam	497815	NM_013150	1	1	0	1	3
rno-miR-124-3p	MIMAT0000828	Fam180a	362336	NM_001108621	1	1	0	1	3
rno-miR-124-3p	MIMAT0000828	Rps6ka1	81771	NM_031107	1	1	0	1	3
rno-miR-124-3p	MIMAT0000828	Sorbs1	686098	XM_003749111	1	1	0	1	3
rno-miR-124-3p	MIMAT0000828	Dclre1b	310745	NM_001025687	1	1	0	1	3
rno-miR-124-3p	MIMAT0000828	Dact1	500666	XM_001077448	1	1	0	1	3
rno-miR-124-3p	MIMAT0000828	LOC1009	100912585	XM_003750846	1	1	0	1	3
rno-miR-124-3p	MIMAT0000828	Hemgn	113882	NM_133294	1	1	0	1	3
rno-miR-124-3p	MIMAT0000828	Lrch4	360779	NM_001127551	1	1	0	1	3
